# Use of non-small cell lung cancer multicellular tumor spheroids to study the impact of chemotherapy

**DOI:** 10.1186/s12931-024-02791-5

**Published:** 2024-04-05

**Authors:** Pauline Hulo, Sophie Deshayes, Judith Fresquet, Anne-Laure Chéné, Stéphanie Blandin, Nicolas Boisgerault, Jean-François Fonteneau, Lucas Treps, Marc G Denis, Jaafar Bennouna, Delphine Fradin, Elvire Pons-Tostivint, Christophe Blanquart

**Affiliations:** 1 Nantes Université, Inserm UMR 1307, CNRS UMR 6075, Université d’Angers, Nantes, CRCI2NA, F- 44000 France; 2 Medical oncology, Nantes Université, CHU Nantes, Nantes, F-44000 France; 3 Service de pneumologie, L’institut du thorax, Hôpital Guillaume et René Laennec, CHU Nantes, Nantes, France; 4 Nantes Université, CHU Nantes, CNRS, Inserm, BioCore, US16, SFR Bonamy, Nantes, F-44000 France; 5 Department of Biochemistry, Nantes Université, CHU Nantes, Nantes, F-44000 France

**Keywords:** Lung cancer, Chemotherapies, Spheroids, Co-culture and PD-L1

## Abstract

**Background:**

Lung cancers represent the main cause of cancer related-death worldwide. Recently, immunotherapy alone or in combination with chemotherapy has deeply impacted the therapeutic care leading to an improved overall survival. However, relapse will finally occur, with no efficient second line treatment so far. New therapies development based on the comprehension of resistance mechanisms is necessary. However, the difficulties to obtain tumor samples before and after first line treatment hamper to clearly understand the consequence of these molecules on tumor cells and also to identify adapted second line therapies.

**Methods:**

To overcome this difficulty, we developed multicellular tumor spheroids (MCTS) using characterized Non-Small Cell Lung Cancer (NSCLC) cell lines, monocytes from healthy donors and fibroblasts. MCTS were treated with carboplatin-paclitaxel or -gemcitabine combinations according to clinical administration schedules. The treatments impact was studied using cell viability assay, histological analyses, 3’RNA sequencing, real-time PCR, flow cytometry and confocal microscopy.

**Results:**

We showed that treatments induced a decrease in cell viability and strong modifications in the transcriptomic profile notably at the level of pathways involved in DNA damage repair and cell cycle. Interestingly, we also observed a modification of genes expression considered as hallmarks of response to immune check point inhibitors and immunogenicity, particularly an increase in *CD274* gene expression, coding for PD-L1. This result was validated at the protein level and shown to be restricted to tumor cells on MCTS containing fibroblasts and macrophages. This increase was also observed in an additional cell line, expressing low basal *CD274* level.

**Conclusions:**

This study shows that MCTS are interesting models to study the impact of first line therapies using conditions close to clinical practice and also to identify more adapted second line or concomitant therapies for lung cancer treatment.

**Supplementary Information:**

The online version contains supplementary material available at 10.1186/s12931-024-02791-5.

## Background

Lung cancer is the main cause of cancer-related deaths worldwide. Non-small cell lung cancer/carcinoma (NSCLC) is the main pathological subtype of lung cancers, accounting for about 80–85% of them [1], and most of the patients are diagnosed at an advanced stage. Immune checkpoint inhibitors (ICIs) are a revolutionary milestone in the field of thoracic oncology, mostly represented by anti-PD-1 (programmed death-1) and anti-PD-L1 (programmed death - ligand 1, also called B7-H1 or CD274) molecules. Over the last decade, ICIs alone or in combination with chemotherapy have been the new standard of care in advanced, unresectable NSCLC with no oncogene drivers, regardless histologic subtypes [1, 2]. Several ICIs such as **p**embrolizumab, cemiplimab, or atezolizumab improved overall survival (OS) compared to chemotherapy in untreated NSCLC patients. However, their efficacy was restricted to patients with high PD-L1 expression on tumor cells (tumor proportion score) ≥ 50% [2]. Afterwards, several trials showed that a combination of platinum-based chemotherapy plus ICIs was superior to chemotherapy alone, regardless of PD-L1 expression [3–6]. More recently, it has been demonstrated that ICIs in combination with platinum-based chemotherapy improve event-free-survival over chemotherapy alone (OS data were still immature but seemed in favor of the combination arm) [7, 8] in patients with localized NSCLC treated in the neoadjuvant setting. Thus, platinum-based chemotherapy remains the backbone of NSCLC treatment, in localized or advanced stage with no targetable oncogenic alterations.

Cytotoxic chemotherapies change tumors biological characteristics. Nevertheless, the way chemotherapy might affect tumor cells, microenvironment cells or both, and the way it might also sensitize them to ICIs remains unclear. A good understanding of therapies consequences on cancer cells should help to improve current first line treatment efficiency, and also to propose more adapted second line therapies. Indeed, at relapse, docetaxel or pemetrexed (for non-squamous histology) as single agents are preferred therapeutic options if not administered at frontline, regardless resistance mechanisms [2]. The reason is that the study of resistance mechanisms in patients is very challenging, because it would require a new biopsy from the recurrence site. To overcome these limitations, preclinical models that help to assess the molecular impact of systemic therapies on tumor cells are highly required. Indeed, the usual 2D cell culture lacks the structural architecture and tumor microenvironment (TME). Interestingly, cell culture in three dimensions (3D) notably reproduces biological barriers that greatly hinder drug delivery and cell to cell interactions that might affect the epithelial to mesenchymal transition state of cells. These models are nowadays mainly used to assess therapies efficacy and they represent a growing field of development, in particular those including TME cells [9].

In this work, we used multicellular tumor spheroid (MCTS) models constituted of NSCLC tumor cells, with or without microenvironment, fibroblasts and monocytes, so as to study the impact of platinum-based chemotherapies, carboplatin-paclitaxel or carboplatin-gemcitabine. We also assessed the impact of this treatment using 3’RNA sequencing, flow cytometry and real-time PCR.

## Methods

### Cells

Human monocytes were freshly isolated by magnetic sorting from Peripheral Blood Mononuclear Cell (PBMC) of healthy volunteers following the manufacturer’s protocol (classical monocyte isolation kit, Miltenyi Biotec). Lung cancer cell line ADCA117 was established from the pleural fluid of a NSCLC cancer patient [10]. ADCA117 cell line belongs to a biocollection of samples from patients at Nantes University Hospital, collected in accordance with the standards established by the Declaration of Helsinki. Recruited patients had received no prior anticancer therapies and provided signed informed consent. All the collected samples and the associated clinical information were registered in a database (DC-2017-2987) validated by the French Ministry of Research. This study was approved by a local ethical committee (CPP Ouest-IV-Nantes). The other NSCLC cell lines (H1975 and H1437) as well as Human Foreskin Fibroblast-2 (HFF-2) cell line were all purchased from ATCC (LGC Standards). The characteristics of lung cancer cell lines used are summarized in table S1. ADCA117 GFP and HFF-2 ruby were obtained by transduction using lentiviral particles and were selected using puromycin at 5 µg/ml (Sigma-Aldrich). These cell lines were cultured in complete RPMI-1640 or DMEM 4.5 g/l Glucose media (Gibco) supplemented with 2mM L-glutamine, 100IU/mL penicillin, 0.1 mg/mL streptomycin (Gibco) and 10% heat-inactivated fetal calf serum (FCS) (Gibco), respectively, at 37 °C and 5% CO2 atmosphere.

### Multicellular tumor spheroids (MCTS) formation

Tumor cells were mixed with or without monocytes from healthy donors and HFF-2 (MCTS complex) at a ratio of 2:0.5:0.5 (2 × 10^4^ total cells) in 96-well U bottom plates NUNCLON SPHERA (Thermo Fisher Scientific) and in a volume of 180 µL of complete culture medium. The plates were centrifuged 2 min at 800×g and incubated at 37 °C in a 5% CO2 atmosphere for 3 days.

### Drugs

Carboplatin, paclitaxel and gemcitabine were obtained from Nantes University Hospital’s drugstore.

### Viability measurement

After treatments, MCTS were collected and cell viability was measured using CellTiter-Glo® Luminescent Cell Viability Assay (Promega) according to the manufacturer’s recommendations using a luminometer (Mithras LB 940, Berthold Technologies).

### 3’RNASeq

MCTS were treated with both carboplatin 50µM and paclitaxel 150nM or carboplatin 50µM and gemcitabine 100nM at day 3, and after by paclitaxel 150nM or gemcitabine 100nM alone at days 6 and 8. At day 10, MCTS were collected and mRNA were extracted using Nucleospin RNA kit (Macherey-Nagel). The mRNA quality was analyzed using Agilent 20,100 Bioanalyzer (Agilent) in RNA Nanochips (Agilent).

Regarding gene analysis, 3′ sequencing RNA profiling was performed by the GenoBird plateform (IRS-UN, Nantes, France) using a NovaSeq 6000 (Illumina Inc.) and NovaSeq 6000 SP Reagent Kit 100 cycles (Illumina Inc.) according to the manufacturer’s protocol (NovaSeq 6000 Sequencing System Guide Document #1000000019358v11 Material #20,023,471, Illumina). The full procedure is described in Charpentier et al., Protocol exchange (10.21203/rs.3.pex-1336/v1). The raw sequence reads were filtered based on quality using FastQC. Adapted sequences were trimmed off the raw sequence reads using Cutadapt. Reads were then aligned to the reference genome using BWA. Moreover, differential expressions were detected with DESeq2 Bioconductor package.

### Flow cytometry

MCTS were treated with carboplatin 50µM and paclitaxel 150nM or gemcitabine 100nM at day 3 and with paclitaxel 150nM or gemcitabine 100nM at days 6 and 8. At day 10, MCTS were collected and treated with TrypLE express (Gibco) for 10 to 120 min at 37°c. Dissociated cells were incubated with an anti-PD-L1 coupled to phycoerythrin (PE) (557,924, BD), an anti-CD14 coupled to allophycocyanin (APC) (301,808, Biolegend), an anti-CD163 coupled to BV421 (333,611, Biolegend) or corresponding control isotypes. After two washes with Phosphate Buffered Saline (PBS), cells were analyzed using FACSymphony™ A5 flow cytometer (BD Biosciences) and DIVA 8 software (BD Biosciences).

### Real time PCR

mRNA were extracted using Nucleospin RNA kit (Macherey-Nagel). 0.5 µg of total RNA were reverse transcribed using MMLV reverse transcriptase (Invitrogen). PCR reactions were performed using QuantiTect Primer Assays (Qiagen) and RT^2^ Real-time SYBR-Green/ROX PCR mastermix (Qiagen) and carried out using QuantStudio™ Real-Time PCR system 3 (ThermoFisher). RPLP0 gene expression was used as internal standard.

### Confocal microscopy

MCTS made with fluorescent cells were collected, washed once in PBS and fixed in 4% paraformaldehyde (Electron Microscopy Sciences) for 48 h at RT. MCTS were washed once with PBS and permeabilized for 24 h with PBS containing 2% Triton X-100 at RT. This solution was removed and afterwards MCTS were incubated with a solution of PBS containing 1% BSA, 0.2% Triton X-100 and Hoescht 5 µg/mL (Sigma-Aldrich) for 48 h at 4 °C. Two washing steps were performed with PBS containing 3% NaCl and 0.2% Triton X-100 for 2 h at RT. Finally, MCTS were resuspended in a RapiClear solution (SunJin Lab) and observed with a confocal microscope (Nikon A1R Si).

### Statistical analysis

Control and treated groups were compared with Mann-Whitney nonparametric t-test. Error bars represent standard errors of mean (SEM). The symbols correspond to a *P*-value inferior to *0.05, **0.01, ***0.001, ****0.0001. All statistical analyses were performed using GraphPad Prism Software (version 8.0).

## Results

### Determination of chemotherapy optimal doses

**Fig. 1:**
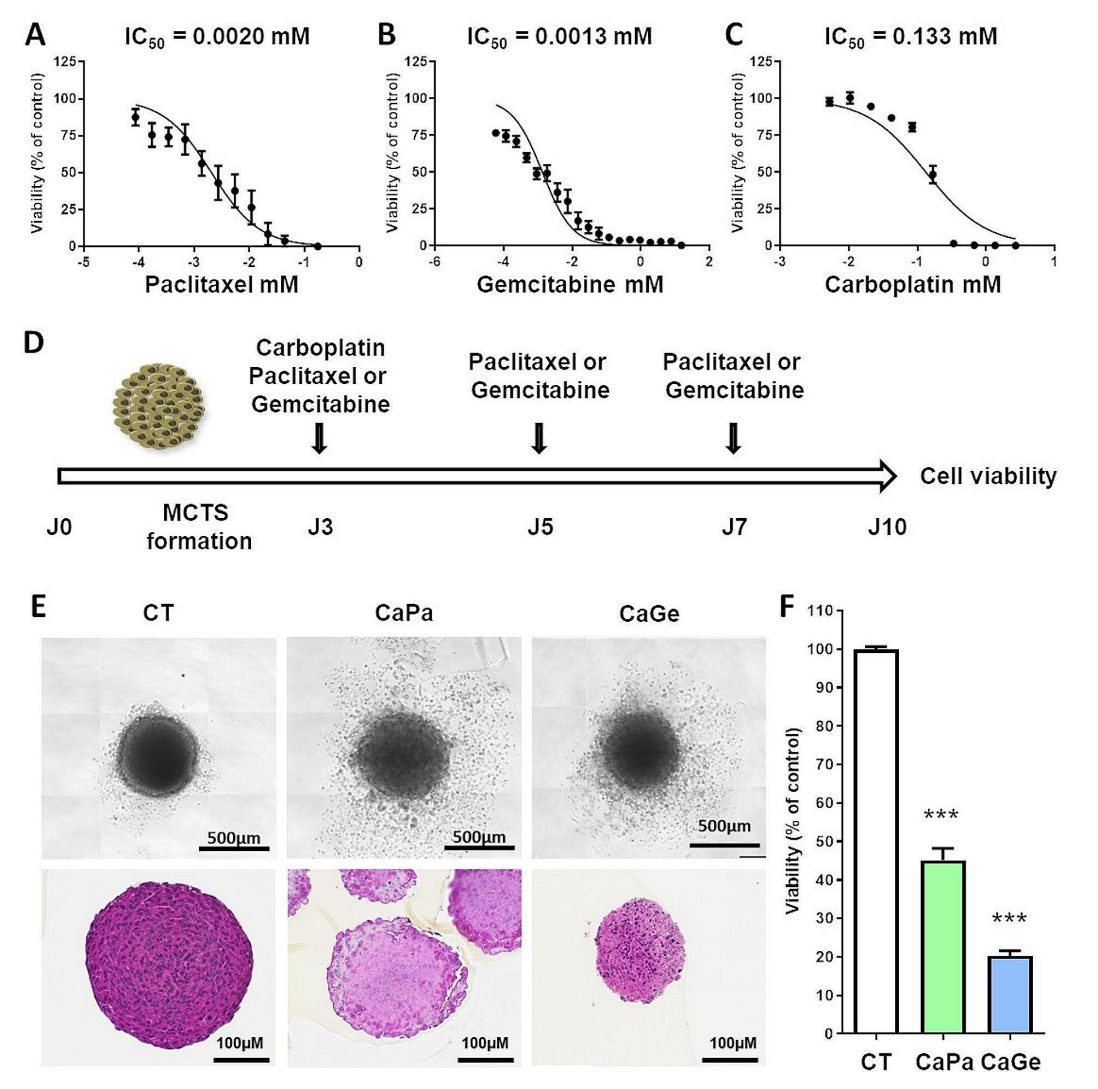
Determination of the optimal doses of carboplatin, paclitaxel and gemcitabine. Lung cancer cells, ADCA117, were grown as MCTS. MCTS were then incubated for 72 h with increasing concentrations of paclitaxel (**A**), gemcitabine (**B**) or carboplatin (**C**, **D**) Treatment sequence of MCTS. (**E**, **F**) MCTS were treated with a combination of carboplatin 50µM and paclitaxel 150nM (CaPa) or gemcitabine 100nM (CaGe) repeated 3 times. (**E**) MCTS were observed using an optical microscopy (upper panel) or histology using hematoxylin, phloxin, safran staining (lower panel) and (**F**) cell viability was measured. Results are expressed as the mean +/- SEM of three independent experiments. ***, *p* < 0.001 (Mann-Whitney t-test)

In order to identify treatment conditions inducing 20–30% of cell death in our experiments, we first determined the sensitivity of ADAC117 cells cultured as MCTS to carboplatin, paclitaxel and gemcitabine by performing dose response experiments using molecules as monotherapy and measuring cell viability after 72 h of incubation. ADCA117 MCTS were most sensitive to paclitaxel (IC_50_ = 0.0020 mM, 95% CI = 0.0014 to 0.0029) (Fig. 1A) and to gemcitabine (IC_50_ = 0.0013 mM, 95% CI = 0.0010 to 0.0018) (Fig. 1B) compared to carboplatin (IC_50_ = 0.133 mM, 95% CI = 0.094 to 0.188) (Fig. 1C). As observed in clinical practice, chemotherapy schedule consisted in an initial combination of carboplatin-paclitaxel (CaPa) or carboplatin-gemcitabine (CaGe) followed by repeated infusion of paclitaxel or gemcitabine as illustrated in Fig. 1D. Individual doses of molecules were chosen in order to induce a decrease of about 20–30% in cell viability after 72 h of treatment according to Fig. 1A, B and C. As observed in Fig. 1E and F, treatments affected MCTS structure and decreased cell viability of approximately 60% and 80% for CaPa and CaGe, respectively.

**Fig. 2:**
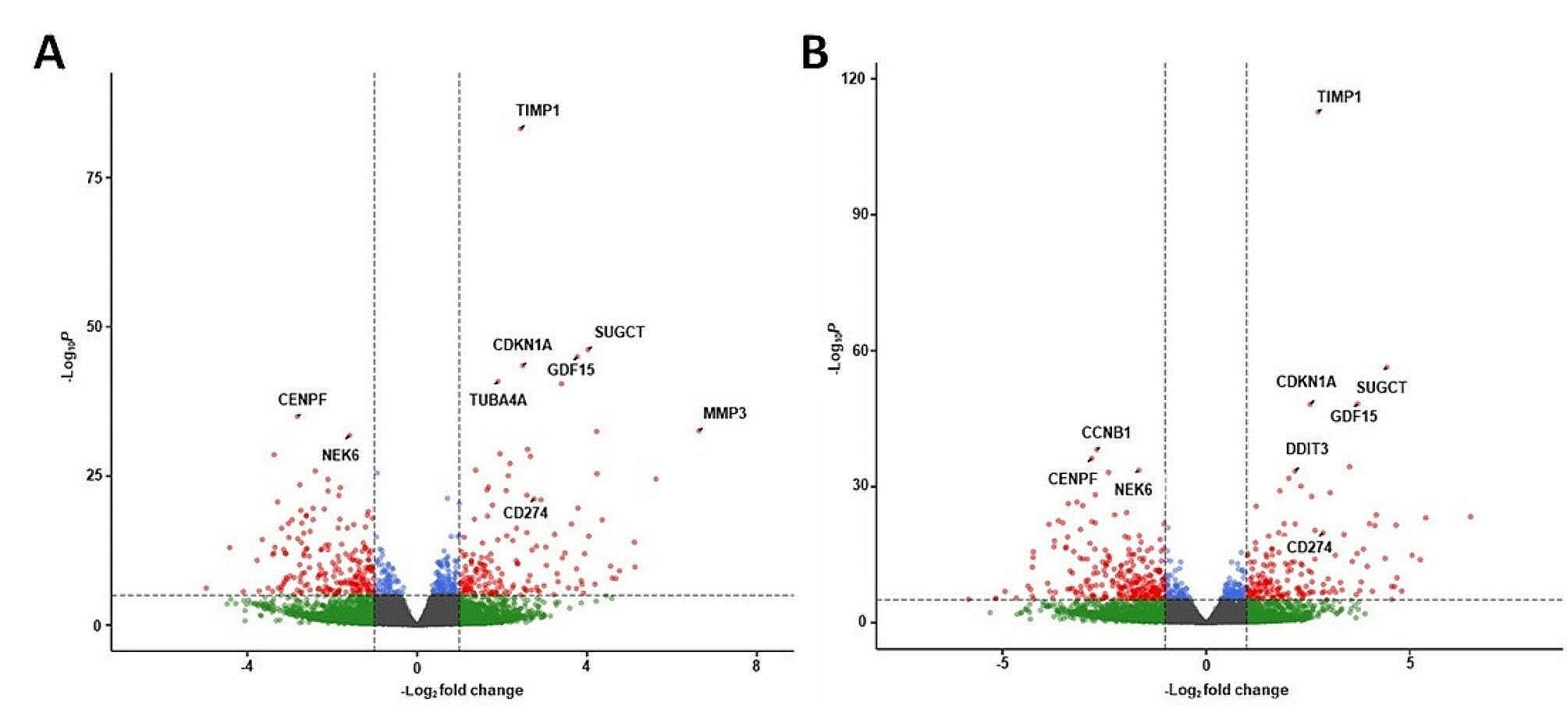
mRNA expression changes in ADCA117 MCTS after exposure to carboplatin-paclitaxel or carboplatin-gemcitabine combinations. ADCA117 MCTS were non-treated (*n* = 13) or treated with a combination of carboplatin (50µM)-paclitaxel (150nM) (CaPa) (*n* = 5) or gemcitabine (100 nM) (CaGe) (*n* = 5) repeated 3 times. mRNA were extracted and analyzed using 3’RNA sequencing. (**A**, **B**) Volcano-plot of the differentially expressed mRNAs after CaPa (**A**) or CaGe (**B**) exposure. Red: significant regulation, Green: non-significant regulation, Blue: significant regulation but under fold-change (FC) cutoff. FC cutoff ≥ 1 or ≤-1

With the objective to identify transcriptomic modifications following CaPa or CaGe in lung cancer cells, we performed a 3’RNA sequencing analysis. Two hundred and seventy one genes were up-regulated (padj < 0.01 and Log2 fold change > 1) and 364 genes were down-regulated (padj < 0.01 and Log2 fold change < -1) in cells exposed to CaPa compared to non-exposed ones (Fig. 2A and table S2). Regarding CaGe, 286 genes were up-regulated (padj < 0.01 and Log2 fold change > 1) and 417 genes were down-regulated (padj < 0.01 and Log2 fold change < -1) in MCTS cells (Fig. 2B and Table S3).

**Fig. 3:**
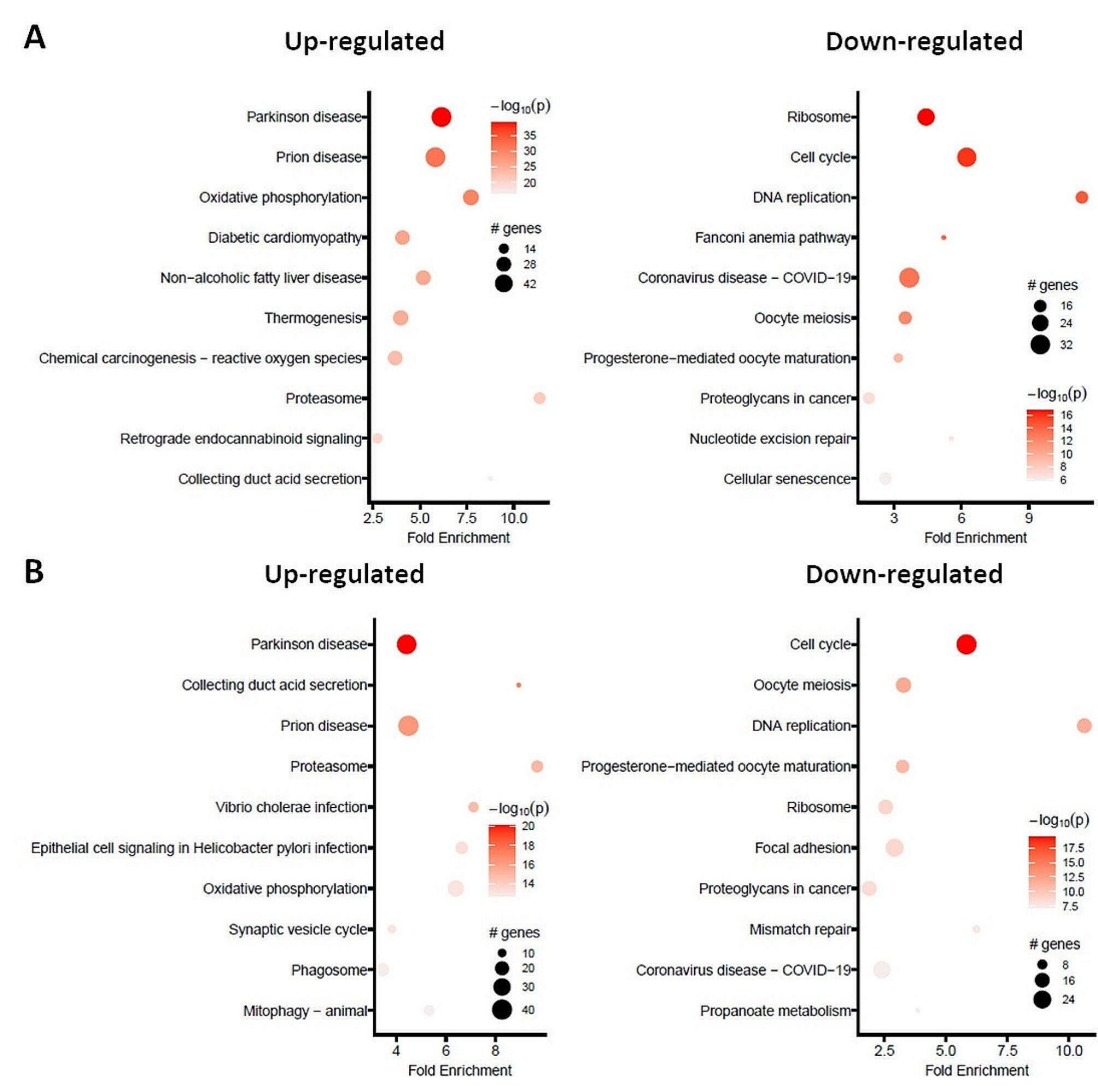
Pathway analysis on the effect of carboplatin-paclitaxel or carboplatin-gemcitabine combinations exposure in ADCA117 MCTS. ADCA117 MCTS were treated with a combination of carboplatin (50µM)-paclitaxel (150nM) (CaPa) or gemcitabine (100 nM) (CaGe) repeated 3 times. mRNA were extracted and analyzed using 3’RNA sequencing. (**A**, **B**) KEGG pathways enrichment of CaPa (**A**) or CaGe (**B**)-dysregulated mRNAs

The lists containing the 10 most significantly regulated genes following CaPa or CaGe are presented in Tables 1 and 2, respectively. Pathway enrichment analyses showed that pathways involved in cell stress were up-regulated (Fig. 3, left panels) whereas those involved in cell functions such as cell cycle and DNA repair were down-regulated (Fig. 3, right panels), regardless the type of chemotherapy. Analysis of the genes affected by both CaPa and CaGe showed that 202 genes were co-upregulated and 285 genes co-downregulated (Figure S1).

**Table 1:** List of the 10 most significantly regulated genes following carboplatin-paclitaxel treatment

Gene	Log2 fold change	P value	Adjusted p value
*MMP3*	6.63	2.76e-33	3.68e-30
*LINC01588*	4.22	3.44e-33	4.08e-30
*SUGCT*	4.02	6.52e-47	3.47e-43
*GDF15*	3.77	1.05e-45	3.74e-42
*C1QTNF1-AS1*	3.4	3.52e-41	6.25e-38
*CDKN1A*	2.48	3.19e-44	8.51e-41
*TIMP1*	2.43	6.03e-84	6.43e-80
*TUBA4A*	1.9	1.52e-41	3.24e-38
*NEK6*	−1.58	1.71e-32	1.82e-29
*CENPF*	−2.81	1.08e-35	1.65e-32

**Table 2:** List of the 10 most significantly regulated genes following carboplatin-gemcitabine treatment

Gene	Log2 fold change	P value	Adjusted p value
*SUGCT*	4.43	5.16e-57	2.87e-53
*GDF15*	3.72	5.96e-49	1.88e-45
*C1QTNF1-AS1*	3.52	4.48e-35	7.12e-32
*TIMP1*	2.74	2.32e-113	2.59e-109
*CDKN1A*	2.55	6.77e-49	1.88e-45
*DDIT3*	2.18	3.84e-34	4.75e-31
*NEK6*	−1.65	2.68e-34	3.73e-31
*PLAC8*	−2.39	7.88e-34	8.77e-31
*CCNB1*	−2.67	6.85e-39	1.52e-35
*CENPF*	−2.8	6.26e-37	1.16e-33

### Impact of CaPa and CaGe on immune status of lung cancer cells

**Fig. 4:**
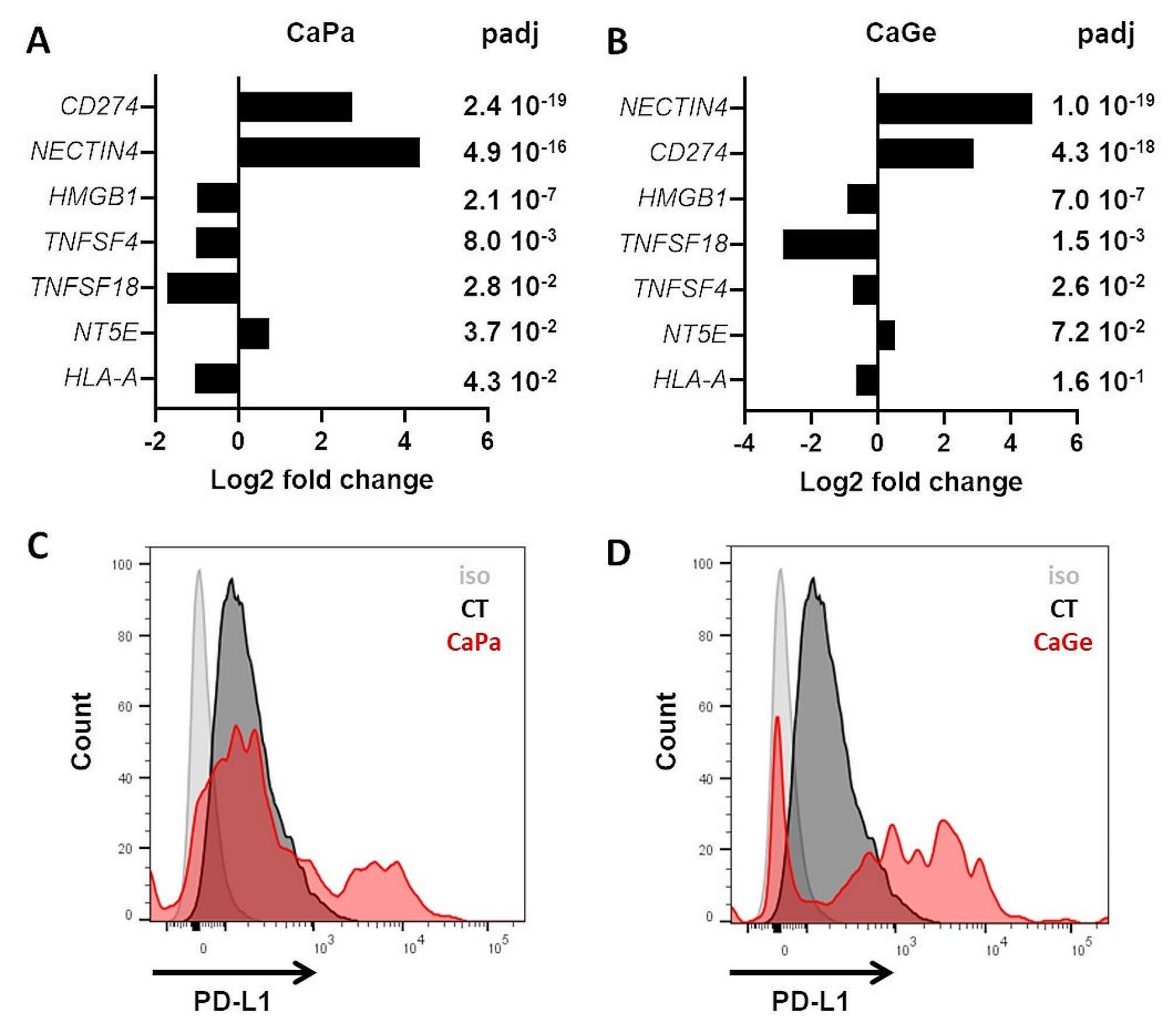
PD-L1 (*CD274*) expression is induced following carboplatin-paclitaxel and carboplatin-gemcitabine exposure. (**A**, **B**) Changes in the expression of genes involved in immunogenicity and check point inhibitor resistance in ADCA117 MCTS after CaPa (**A**) or CaGe (**B**) treatment using 3’RNASeq data. (**C**, **D**) PD-L1 expression was measured following CaPa (**C**) or CaGe (**D**) treatment using flow cytometry after MCTS dissociation

Immune check point inhibitors (ICI) have strongly modified therapeutic strategies in NSCLC patients, and are currently a standard of care, alone or in combination with chemotherapy. Thus, we have analyzed the effect of CaPa and CaGe treatments on the expression of a panel of genes considered as hallmarks of response/resistance to immune check point inhibitors [11] from data obtained through 3’RNA sequencing. Among this panel, mRNA expression of 3 genes were upregulated after CaPa, *CD274*, coding for PD-L1, *NECTIN4*, and *NT5E*, coding for 5’ Nucleotidase (Fig. 4A) (Table 3). Expression of four genes decreased after CaPa, *TNFSF18*, coding for GITRL, *HMGB1*, *TNFSF4*, coding for OX-40 L and *HLA-A*, all involved in the positive regulation of anti-tumor immune response (Fig. 4A)(Table 3). The genes most significantly deregulated with CaGe were common with CaPa and included *NECTIN4*, *CD274*, *HMGB1*, *TNFSF18* and *TNFSF4* (Fig. 4B; Table 4).

**Table 3:** Impact of CaPa treatment on the expression of hallmark genes of resistance/sensitivity to immune checkpoint inhibitors

Genes	Log2 fold change	P value	Adjusted p value
*CD274*	2.75	7.21e-22	2,40E-19
*NECTIN4*	4.35	2.38e-18	4,97E-16
*HMGB1*	−0.99	4.72e-09	2,13E-07
*TNFSF4*	−1.03	0.0008	0,008
*TNFSF18*	−1.72	0.003	0,028
*NT5E*	0.73	0.005	0,037
*HLA-A*	−1.04	0.006	0,043
*HLA-C*	−0.40	0.010	0,058
*B2M*	−0.28	0.01	0,086
*HLA-E*	−0.33	0.04	0,171
*HLA-B*	−0.24	0.13	0,356
*PVR*	0.69	0.13	0,366
*ICOSLG*	0.93	0.24	0,524
*NECTIN3*	0.37	0.34	0,621
*SELPLG*	−0.33	0.54	0,778
*NECTIN2*	−0.08	0.72	0,886
*CD276*	−0.073	0.86	0,944
*CD47*	0.003	0.99	0,997
*CEACAM1*	1.82	0.12	NA
*TNFRSF14*	1.09	0.28	NA

**Table 4:** Impact of CaGe treatment on the expression of hallmark genes of resistance/sensitivity to immune checkpoint inhibitors

Genes	Log2 fold change	P value	Adjusted p value
*NECTIN4*	4.66	3.35e-22	1.06E-19
*CD274*	2.87	1.60e-20	4.36E-18
*HMGB1*	−0.92	1.74e-08	7.04E-07
*TNFSF18*	−2.83	0.0001	0.0015
*B2M*	−0.36	0.0036	0.026
*TNFSF4*	−0.73	0.012	0.072
*HLA-E*	−0.35	0.039	0.16
*PVR*	0.86	0.060	0.21
*NT5E*	0.51	0.078	0.25
*HLA-A*	−0.64	0.10	0.31
*HLA-C*	−0.21	0.20	0.46
*SELPLG*	0.52	0.25	0.53
*CD47*	0.35	0.27	0.54
*NECTIN2*	−0.20	0.40	0.67
*CD276*	0.28	0.47	0.73
*ICOSLG*	0.58	0.50	0.75
*HLA-B*	−0.10	0.52	0.76
*NECTIN3*	0.15	0.68	0.86
*TNFSF14*	1.01	0.31	NA
*CEACAM1*	1.04	0.45	NA

PD-L1 is a major target in the current strategy of immunotherapy based on the use of check point inhibitors. We therefore focused our work on this protein expressed at the surface of cells and involved in the inhibition of immunologic anti-tumor response. Using flow cytometry, we confirmed the induction of *CD274* gene expression at the protein level in ADCA117 cells that do not express PD-L1 in control conditions (Fig. 4C and D). This induction was not observed in all cells. We observed that treatments strongly affect MCTS, after hematoxylin, phloxin, safran (HPS) staining, and also induce PD-L1 expression, but only at MCTS periphery where cells are in direct contact with chemotherapies (Figure S2A).

### Impact of CaPa and CaGe on PDL-1 expression in MCTS constituted of tumor cells, fibroblasts and macrophages

**Fig. 5:**
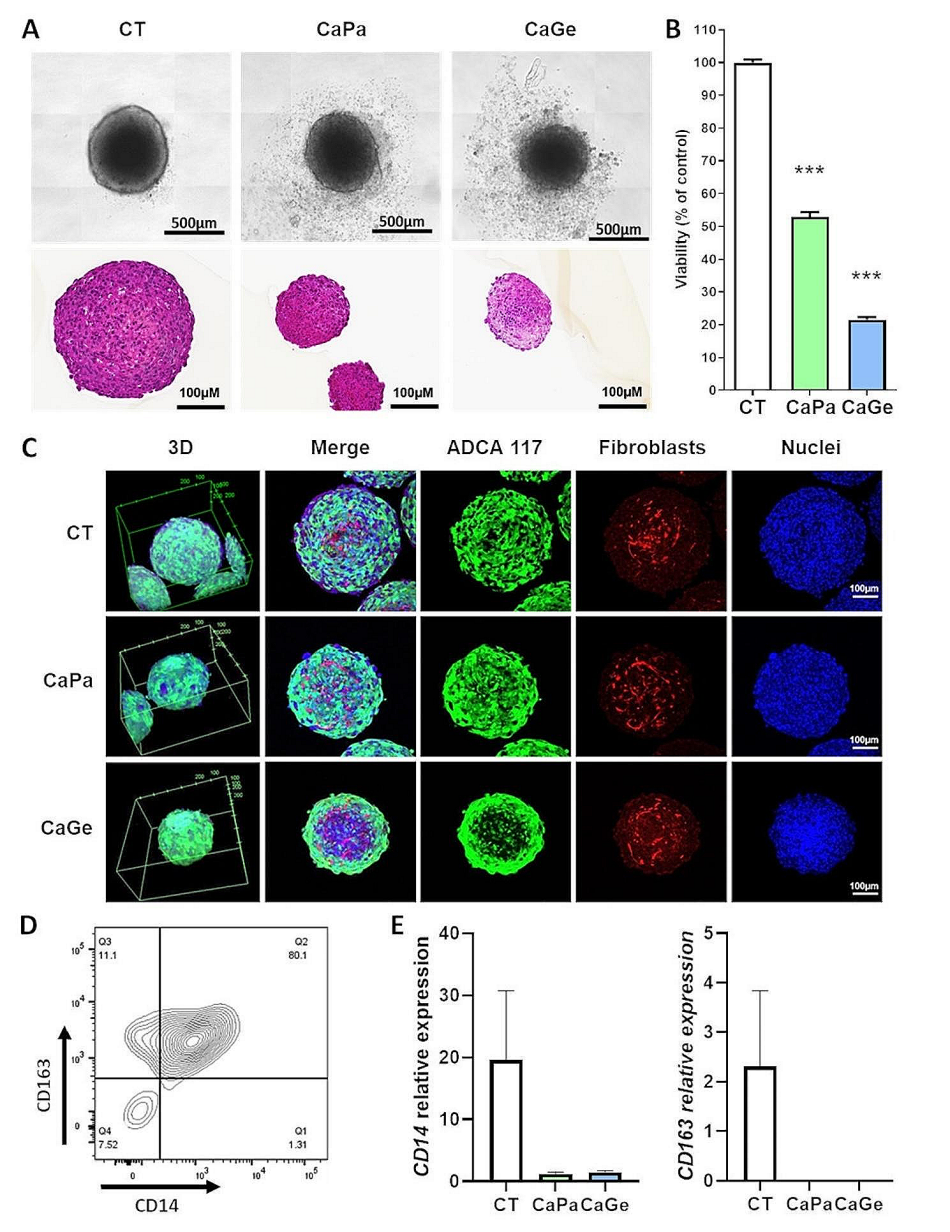
Impact of carboplatin-paclitaxel treatment on complex MCTS viability and structure. Lung cancer cells, ADCA117, fibroblasts (HFF-2) and monocytes were cultured as MCTS. MCTS were treated with a combination of carboplatin 50µM and paclitaxel 150nM (CaPa) or gemcitabine 100nM (CaGe) repeated 3 times. (**A**) MCTS were observed using optical microscopy (upper panel) or histology using hematoxylin, phloxin, safran staining (lower panel) and (**B**) cell viability was measured. Results are expressed as the mean +/- SEM of three independent experiments. ***, *p* < 0.001 (Mann-Whitney t-test). (**C**) MCTS were observed using confocal microscopy. Blue: nuclei, green: ADCA117-GFP and red: HFF-2 ruby. (**D**) MCTS were dissociated and analyzed using flow cytometry. Dot plot shows CD14 and CD163 expression on HLA-DR + and CD14 + cells. (**E**) mRNA were extracted and both *CD14* and *CD163* expressions were measured using real-time PCR. Results are expressed as the mean +/- SEM of the three independent experiments

In order to get closer to malignant tissues complexity, we have included fibroblasts and macrophages, two major components of TME, with ADCA 117 cells to build complex MCTS. CaPa or CaGe treatments altered MCTS structure (Fig. 5A) and significantly decreased global viability by approximately 50% and 80%, respectively (Fig. 5B). Using confocal microscopy, we observed a MCTS size reduction after treatment (Fig. 5C), whereas CaPa seemed not to affect tumor cells at MCTS core, CaGe particularly affected lung cancer cells at MCTS core as illustrated by a loss of GFP fluorescence and the presence of highly compact nuclei in this area (Fig. 5C). Fibroblasts did not appear to be affected by chemotherapy. The fact of adding monocytes into MCTS led to macrophages formation expressing CD14 and CD163 as showed using flow cytometry (Fig. 5D) and real-time PCR (Fig. 5E).

**Fig. 6:**
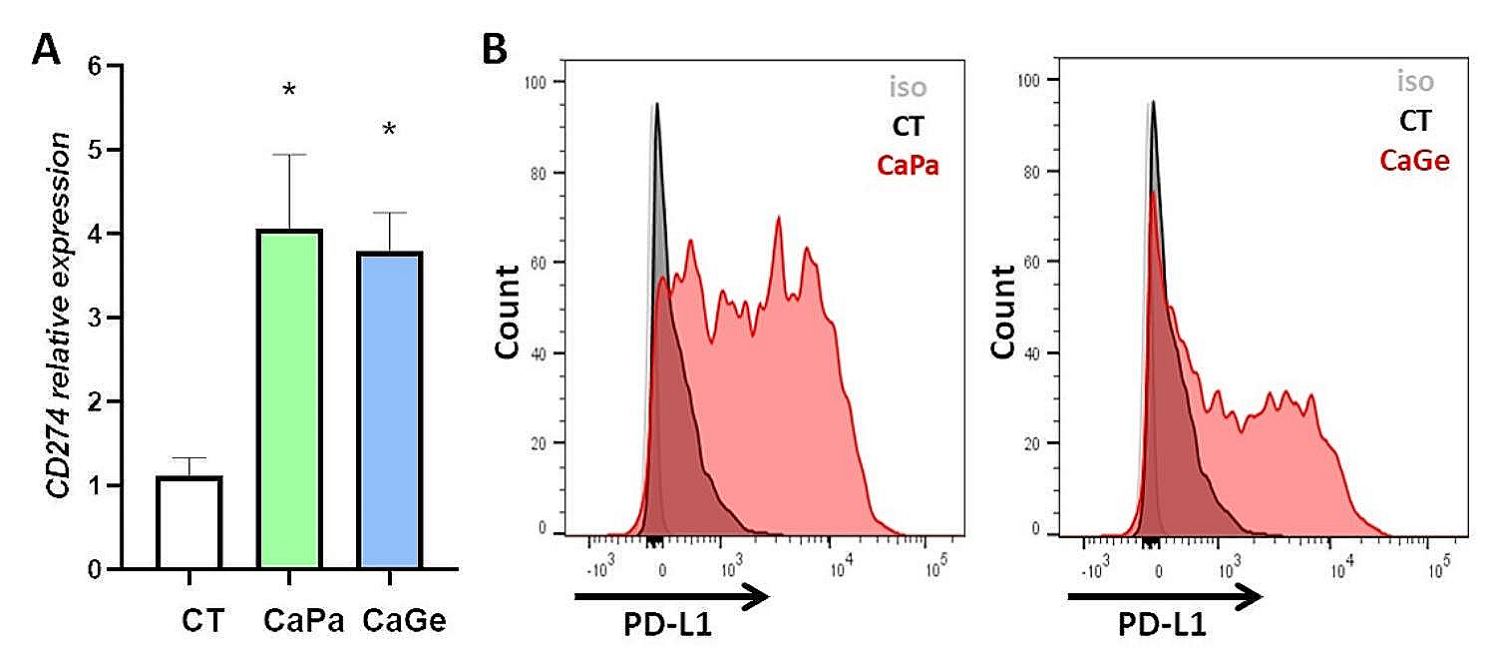
PD-L1 (CD274) expression is induced following CaPa or CaGe exposure in complex MCTS. Lung cancer cells, ADCA117, fibroblasts (HFF-2) and monocytes were grown as MCTS. MCTS were treated with a combination of carboplatin 50µM and paclitaxel 150nM (CaPa) or gemcitabine 100nM (CaGe) repeated 3 times. (**A**) mRNA were extracted and *CD274* expression was measured using RT-PCR. Results are expressed as the mean +/- SEM of at least three independent experiments. *, *p* < 0,05 (Mann-Whitney t-test). (**B**) PD-L1 expression was measured using flow cytometry after dissociation of MCTS. Left: CaPa; right: CaGe

After CaPa or CaGe, both *CD14* and *CD163* expressions were strongly reduced suggesting macrophages elimination by chemotherapy. MCTS treatment with CaPa or CaGe induced PD-L1 expression at both mRNA (Fig. 6A) and protein level (Fig. 6B). Using flow cytometry, we observed that PD-L1 expression was specifically induced at the surface of cancer cells (Fig. 6B). Indeed, PD-L1 expression was not detected at the surface of fibroblasts and macrophages after CaPa or CaGe (Figure S3). As previously observed using immunohistology, PD-L1 was only expressed at the periphery of treated MCTS (Figure S2B).

**Fig. 7:**
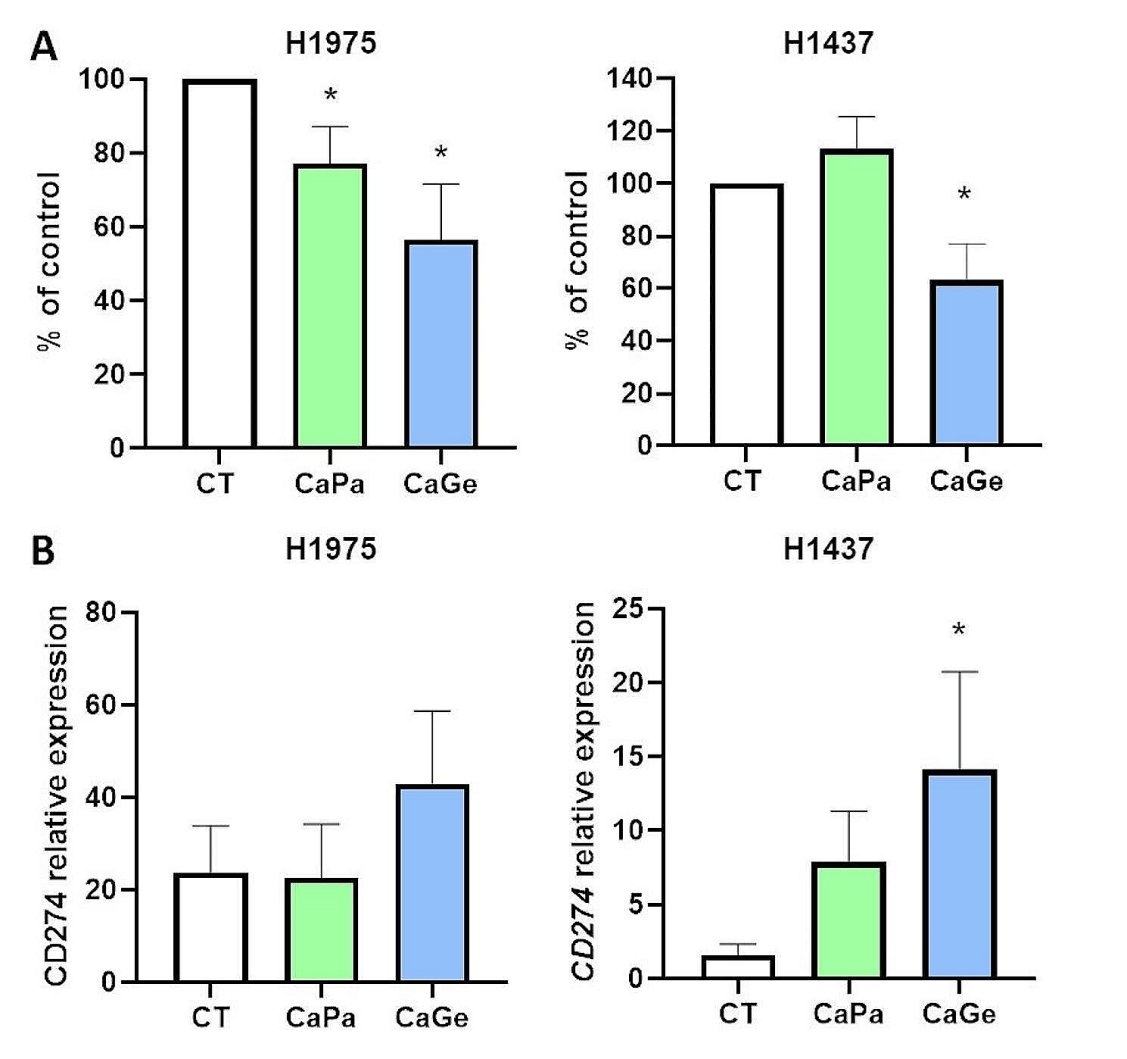
CaPa and CaGe treatment effect on complex MCTS. Lung cancer cells, H1975 or H1437, fibroblasts (HFF-2) and monocytes were grown as MCTS. MCTS were treated with a combination of carboplatin 50µM and paclitaxel 150nM (CaPa) or gemcitabine 100nM (CaGe) repeated 3 times. (**A**) Cells viability was measured. (**B**) mRNA from cells were extracted and CD274 expression was measured using real-time PCR. Results are expressed as the mean +/- SEM. *n* = 5. *, *p* < 0.05 (Mann-Whitney t-test)

To extend our work to additional models of lung cancer MCTS including fibroblasts and macrophages, we selected a cell line expressing a similar level of *CD274* than ADCA117, H1437, as well as a cell line expressing higher level of *CD274* compared to ADCA117, H1975 (Figure S4). We observed heterogeneity in lung cancer cells sensitivity cultured as MCTS to CaPa and to a lesser extend to CaGe. Indeed, ADCA117 MCTS were highly sensitive to CaPa (approximately 55% of cell death) (Fig. 5B) whereas H1975 MCTS were weakly sensitive (approximately 25% of cell death) and H1437 MCTS were resistant (Fig. 7A). Figure 7B shows that CaPa induces *CD274* expression in H1437 but did not modify *CD274* expression in H1975 MCTS already presenting high expression of this protein. Moreover, CaGe induced a significant cell viability decrease in ADCA117 (Fig. 5B, approximately 80% of cell death), H1975 (approximately 45% of cell death) and H1437 (approximately 55% of cell death) MCTS (Fig. 7A) and increased *CD274* expression in H1437 once again but not in H1975 MCTS (Fig. 7B).

**Fig. 8:**
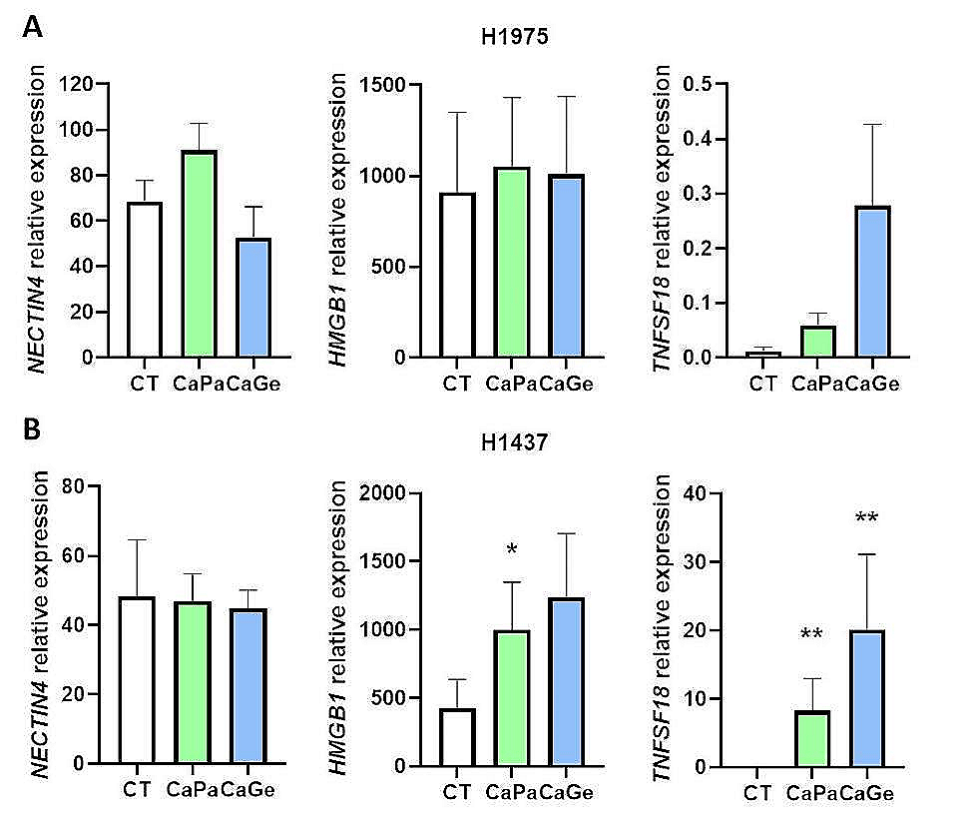
CaPa and CaGe treatment effect on *NECTIN4*, *HMGB1* and *TNFSF18* expressions in complex MCTS. Lung cancer cells, H1975 (**A**) or H1437 (**B**), fibroblasts (HFF-2) and monocytes were grown as MCTS. MCTS were treated with a combination of carboplatin 50µM and paclitaxel 150nM (CaPa) or gemcitabine 100nM (CaGe) repeated 3 times. mRNA from cells were extracted and genes expression was measured using real-time PCR. Results are expressed as the mean +/- SEM. *n* = 5. *, *p* < 0.05; **, *p* < 0.01

In ADCA117 MCTS, we previously observed that *NECTIN4*, *HMGB1* and *TNFSF18* expression was modified after CaPa or CaGe. We studied the impact of treatments on these genes expression (Fig. 8) using H1437 and H1975 complex MCTS. In H1975 complex MCTS, no modifications were observed for *NECTIN4* and *HMGB1* expression (Fig. 8A). A non-significant increase in *TNFSF18* was observed. However, as for *NECTIN4* in ADCA117 MCTS, basal expression was very low compared to ADCA117 (Figure S4). In H1437 complex MCTS, *NECTIN4* expression remained the same after treatments whereas both *HMGB1* (tendency with CaPa)) and *TNFSF18* expressions increased (Fig. 8B).

## Discussion

ICIs development has deeply changed NSCLC therapeutic care of patients over the last decade, by demonstrating survival improvement. Importantly, platinum-based chemotherapy remained the backbone of treatment, in combination with ICIs, regardless tumor stage and histology. However, most patients will finally relapse, and rebiopsies are often difficult to obtain. Chemotherapies impact tumor cells and their microenvironment. However, molecular modifications remain largely unknown due to the challenging access to tumor tissues before and after systemic therapies. In order to overcome this limitation, the objective of our study is to develop and characterize 3D models of lung cancer, including or not TME cells. Indeed, the number of models with TME cells is limited particularly regarding the presence of macrophages. Moreover, to evaluate the impact of chemotherapies, we used a protocol based on chemotherapy cycles administrated in the clinic and not only a treatment during 72 h as it was usually the case in in vitro studies. Thus, we have developed Multicellular Tumor Spheroids (MCTS) models containing NSCLC cells, with or without fibroblasts and monocytes. First, we treated MCTS constituted of tumor cells alone with a combination of chemotherapeutic agents, carboplatin-paclitaxel (CaPa) or carboplatin-gemcitabine (CaGe), according to the treatment schedule used in patients (Fig. 1D). These treatments decreased cell viability and induced strong transcriptomic modifications. In particular, several genes involved in tumor cell immunogenicity [11] were deregulated, such as *CD274* coding for PD-L1, which was induced. *CD274* induction was confirmed at the protein level using flow cytometry and immunohistology. These experiments showed that PD-L1 induction was restricted to a fraction of cells localized at MCTS periphery, those in direct contact with chemotherapies. Then, chemotherapies impact was studied using complex MCTS constituted of NSCLC tumor cells, fibroblasts and monocytes. These treatments reduced cell viability depending on the cancer cell line used to form MCTS. Some MCTS being resistant to treatments, reduced the number of macrophages but had no impact on fibroblasts. A cell immunogenicity modification was also observed. Furthermore, *CD274* gene expression increased, as previously observed, in NSCLC cells with low basal expression, but remained the same in NSCLC cells with a high basal *CD274* expression. Herein, this PD-L1 induction was not either related to the treatment used nor to the presence or not of fibroblasts and macrophages. However, other genes regulation involved in anti-tumor immune response and resistance to immunotherapies, *NECTIN4*, *HMGB1*, *TNFSF18*, by CaPa or CaGe seem to be dependent on the cell line used and thus to be more heterogeneous.

Paclitaxel and gemcitabine belong respectively to taxane, a family of drugs interfering with microtubule during cell division, and nucleoside, a family of metabolic inhibitors. In regards of their different action mode, numbers of genes are differentially regulated when using CaPa or CaGe combinations. CaPa induced specifically genes expression involved in inflammatory pathways such as TNFα signaling, inflammatory response and interferon-γ response, and specifically reduced pathways involved in cell division such as E2F targets, G2M checkpoint and mitotic spindle (Figure S1B and Table S4). CaGe specifically induced genes expression involved in pathways such as DNA damages with P53 and UV response, and reduced, therefore, pathways involved in inflammation such as interferon-γ and interferon-α responses (Figure S1C and Table S5). Nevertheless, most of deregulated genes are common to the two combinations and suggests a strong impact of carboplatin, a platin salt. Mainly, genes induced are involved in pathways regulating inflammation and DNA damages like TNFα signaling and inflammatory response, as well as P53 and apoptosis respectively (Figure S1D and Table S6). At the level of immunogenicity, CaGe and CaPa had a similar impact on the hallmark genes of resistance/sensitivity to immune checkpoint inhibitors in ADACA 117. However, in H1437 and H1975, the effect seemed to depend on cells sensitivity to treatments. Indeed, CaGe induced a stronger decrease in cell viability compared to CaPa as well as a higher regulation of *CD274* and *TNFSF18* expression. Therefore, depending on cells, CaGe and CaPa effect might be different on immunogenicity. This illustrates the complexity and heterogeneity of the response to treatments observed in clinical practice [12, 13].

PD-L1, expressed on various solid tumor and immune cells, plays a crucial role in developing cancer immunoresistance by binding PD-1 expressed at the surface of T-lymphocytes, resulting in the inhibition of T-lymphocytes migration, proliferation and secretion of cytotoxic mediators [14]. Thus, PD-L1 is a major target in immunotherapy strategy. We observed that CaPa and CaGe combinations increased PD-L1 expression suggesting a possible conversion of negative/low PD-L1 cells to positive/high PD-L1 cells after chemotherapy. This PD-L1 induction had already been observed in a heterogeneous way in NSCLC tumors using immunohistochemistry and this seemed to depend on the type of chemotherapy used [15–18]. Interestingly, this PD-L1 induction following chemotherapies could explain, at least partially, the superiority of chemotherapy/immunotherapy compared to chemotherapy alone even in patients with a PD-L1 score < 1% in tumor [3, 5, 12, 19, 20]. Likewise, in patients with resectable NSCLC, a neoadjuvant combination of chemotherapy (carboplatin/cisplatin with paclitaxel) plus ICI showed a better efficacy than chemotherapy alone regardless of PDL-1 status suggesting a possible sensitization of tumors to ICIs by inducing PD-L1 expression. In addition, several studies showed a trend to an increase in PD-L1 level at relapse, after a cytotoxic chemotherapy in adjuvant or advanced stage [12, 13]. The reproduction of this chemotherapy effect on PD-L1 expression in MCTS suggests that this 3D model could be an interesting alternative to tumor samples to study treatments impact on tumor cells.

TME cells, including fibroblasts and macrophages, play a major role in the tumors development. Thus, we added these cells in our MCTS to take into account the interactions with tumor-associated macrophages (TAMs) and cancer-associated fibroblasts (CAFs) as well as physical properties of the three-dimensional cell culture [21, 22]. Monocytes differentiated into TAMs expressing CD14 and CD163 markers, usually tend to associate with immunosuppressive M2-like phenotype [23, 24]. We also observed that macrophages were highly sensitive to treatments whereas fibroblasts were not. Several studies have shown a depleting effect of gemcitabine on myeloid derived suppressive cells in NSCLC [25]. This effect was also observed in other types of cancers with chemotherapeutic agents including platinum derivatives, anthracyclines and taxane [25]. This suggests that after chemotherapy, residual tumours could be infiltrated by new monocytes in a new environment, with different phenotypes. It is difficult to generate data in order to confirm TME modification in tumours due to the difficult access to samples before and after treatments. Furthermore, CAFs are a major TME component that contribute to tumor growth, supporting resistance to treatments, migration and invasion [26]. As for monocytes, the presence of fibroblasts did not modify the induction of *CD274* expression following treatments. As opposed to monocytes/macrophages, fibroblasts seemed less sensitive to chemotherapies suggesting a stability of fibroblast populations during tumors development and after treatments [27–29].

## Conclusions

In conclusion, our study suggests that the use of 3D models is of great interest to assess the impact of first line chemotherapy and thus to identify new and more efficient strategies for second line therapies. However, to get closer to physiopathological situation, 3D models improvement remains necessary such as the introduction of other immune cells beside monocytes as well as endothelial compartments among others.

### Electronic supplementary material

Below is the link to the electronic supplementary material.

**Supplementary Figure S1:** Number of genes regulated specifically and in common by CaPa and CaGe treatments. A) Left, vendiagramm showing the number of genes upregulated in common or specifically after CaPa or CaGe treatment. A) Right, vendiagramm showing the number of genes downregulated in common or specifically after CaPa or CaGe treatment. B-D) Hallmark gene sets pathways enrichment of genes specifically regulated by CaPa (B), by CaGe (C) and in common between CaPa and CaGe (D) (GSEA, Human MSigDB v2023.2.Hs updated October 2023)(Subramanian, Tamayo, et al. (2005, PNAS).

**Supplementary Figure S2:** PD-L1 (CD274) expression is induced following carboplatin-paclitaxel exposure in complex MCTS. Lung cancer cells, ADCA117, alone (A) or with fibroblasts (HFF-2) and monocytes (B) were grown as MCTS. MCTS were treated with a combination of carboplatin 50µM and paclitaxel 150nM (CaPa) repeated 3 times. Then, MCTS were analyzed using Immunohistology. Right panel: PD-L1 labelling in MCTS. MCTS were fixed with 4% paraformaldehyde (Electron Microscopy Sciences) for 24 h at room temperature (RT). After one PBS wash, MCTS were included in HistoGel (Microtech, Thermo Fisher Scientific). Then, an immunohistochemical analysis was performed using standard techniques with the Cellular and Tissue Imaging Core Facility of Nantes University (Micro-PICell). Left panel: hematoxylin, phloxin, safran (HPS) MCTS staining. Right panel: antigen retrieval was performed using Impath Retrieval Solution pH 6.0 (Impath, ref: 44,998) at 101 °C for 20 min. Slides were incubated with hydrogen peroxyde blocking solution (Thermo scientific, ref: TA-125-HP) for 10 min then, incubated with rabbit serum 1.5% (Vector Laboratories, ref: S-5000) for 20 min at room temperature. PD-L1 antibody (R&D systems, AF156) was used at 0.5 µg/ml for 1 h then, biotinylated rabbit anti-goat secondary antibody was used at 3.75 µg/ml (Vector Laboratories, ref BA-5000) for 30 min. Revelation was performed using Large Volume Streptavidin peroxidase (Thermo Scientific, ref: TS-125-HR) for 30 min and DAB (3,3′-Diaminobenzidine). Pictures were obtained using a NanoZoomer 2.0HT (Hamamatsu).

**Supplementary Figure S3:** PD-L1 (CD274) expression is not induced in fibroblasts and macrophages following treatment. Lung cancer cells, ADCA117 (GFP +), fibroblasts (HFF-2) and monocytes were grown as MCTS. MCTS were treated with a combination of carboplatin 50µM and carboplatine 150 nM (CaPa) or gemcitabine 100nM (CaGe) repeated 3 times. PD-L1 expression was measured using flow cytometry after MCTS dissociation. PD-L1 expression analysis was performed on GFP – cells.

**Supplementary Figure S4:***CD274*, *NECTIN4*, *HMGB1* and *TNFSF18* genes expression in ADCA117, H1437 and H1975 cells. mRNA of lung cancer cells, ADCA117, H1437 and H1975 were extracted and genes expression was measured using real-time PCR. Results are expressed as the mean +/− SEM of three independent experiments.

Supplementary Material 5

Supplementary Material 6

Supplementary Material 7

Supplementary Material 8

Supplementary Material 9

Supplementary Material 10

## Data Availability

The datasets used and/or analyzed during the current study are available from the corresponding authors on reasonable request. 3’RNAseq data are registered under GSE243934 reference in NCBI’s Gene Expression Omnibus (GEO) database.

## Abbreviations

2D: Two dimension
3D: Three dimension
APC: Allophycocyanin
ATCC: American type culture collection
CAFs: Cancer-associated fibroblasts
CaGe: Carboplatin-gemcitabine
CaPa: Carbolatin-paclitaxel
DNA: deoxyribonucleic acid
FCS: Fetal calf serum
GFP: Green fluorescent protein
HFF2: Human Foreskin Fibroblasts-2
HPS: Hematoxylin, phloxin, safran
ICIs: Immune checkpoint inhibitors
MCTS: Multicellular tumor spheroids
NSCLC: Non-Small Cell Lung Cancer
OS: Overall survival
PBMC: Peripheral Blood Mononuclear Cell
PBS: Phosphate buffered saline
PCR: Polymerase Chain Reaction
PD-1: Programmed death-1
PD-L1: Programmed death - ligand 1
SEM: Standard errors of mean
TAMs: Tumor-associated macrophages
