# Supplementary figures and images for: Use of non-small cell lung cancer multicellular tumor spheroids to study the impact of chemotherapy
Source: Respir Res. 2024 Apr 5;25:156. doi: 10.1186/s12931-024-02791-5 (PMC10998296; doi:10.1186/s12931-024-02791-5)

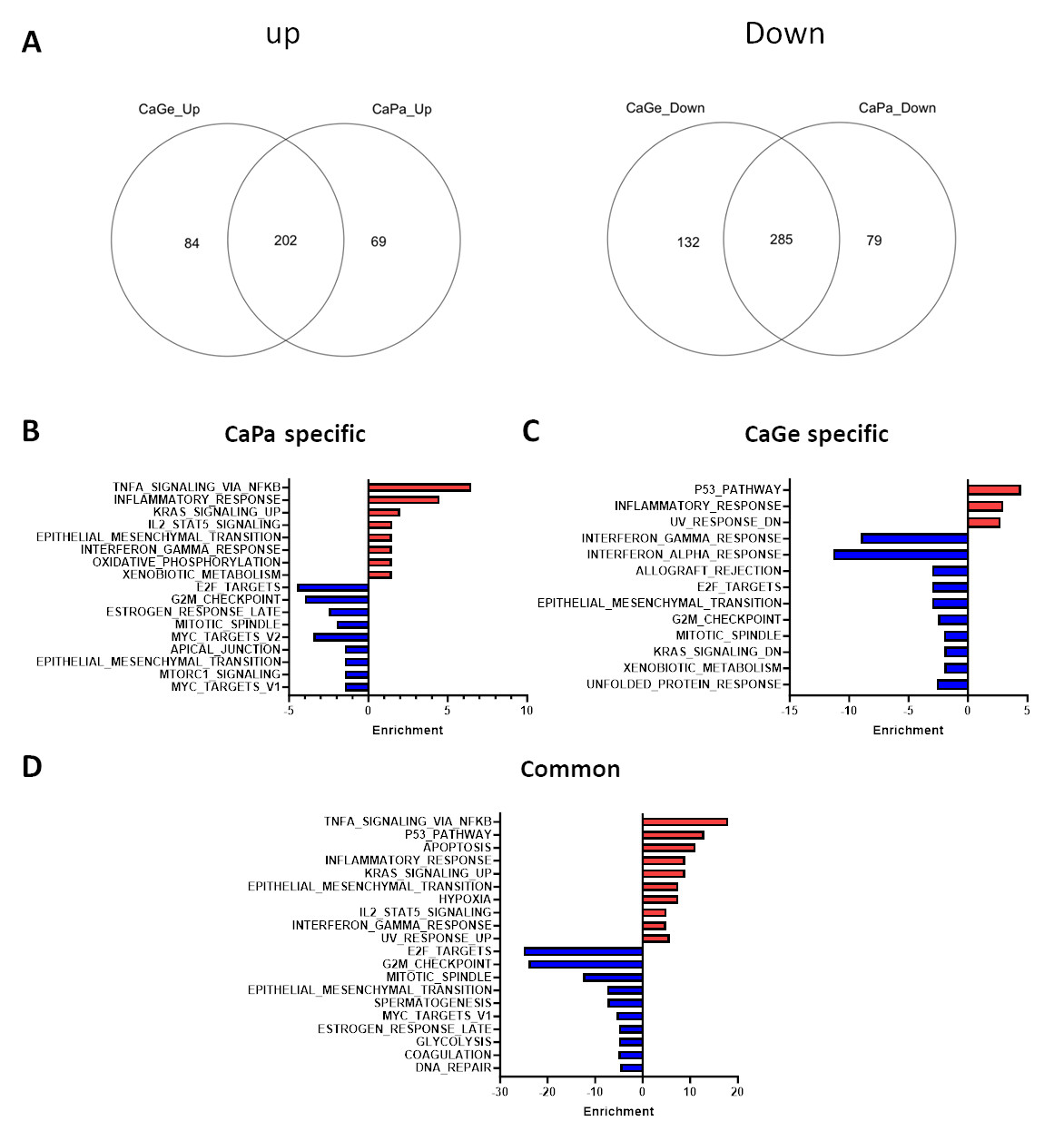

Supplement: Supplementary file 1 — Supplementary Figure S1: Number of genes regulated specifically and in common by CaPa and CaGe treatments. A) Left, vendiagramm showing the number of genes upregulated in common or specifically after CaPa or CaGe treatment. A) Right, vendiagramm showing the number of genes downregulated in common or specifically after CaPa or CaGe treatment. B-D) Hallmark gene sets pathways enrichment of genes specifically regulated by CaPa (B), by CaGe (C) and in common between CaPa and CaGe (D) (GSEA, Human MSigDB v2023.2.Hs updated October 2023)(Subramanian, Tamayo, et al. (2005, PNAS). [file 12931_2024_2791_MOESM1_ESM.jpg]

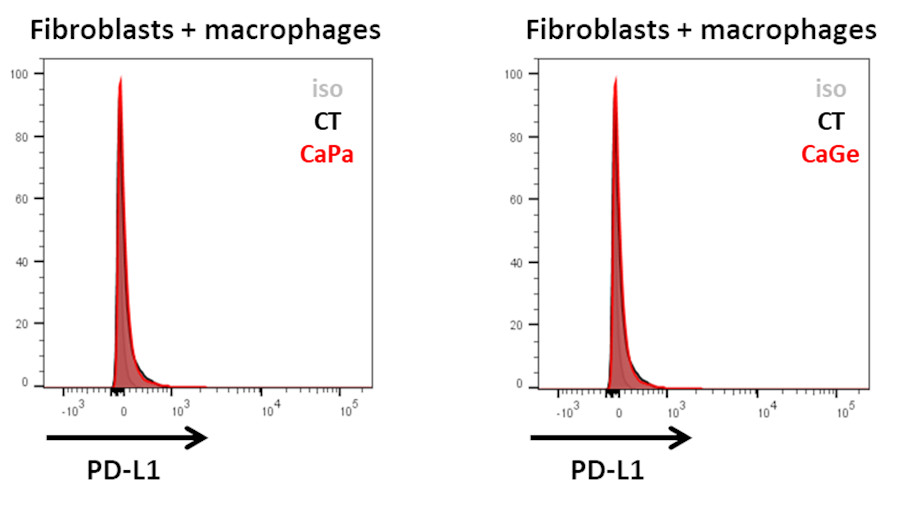

Supplement: Supplementary file 2 — Supplementary Figure S2: PD-L1 (CD274) expression is induced following carboplatin-paclitaxel exposure in complex MCTS. Lung cancer cells, ADCA117, alone (A) or with fibroblasts (HFF-2) and monocytes (B) were grown as MCTS. MCTS were treated with a combination of carboplatin 50µM and paclitaxel 150nM (CaPa) repeated 3 times. Then, MCTS were analyzed using Immunohistology. Right panel: PD-L1 labelling in MCTS. MCTS were fixed with 4% paraformaldehyde (Electron Microscopy Sciences) for 24 h at room temperature (RT). After one PBS wash, MCTS were included in HistoGel (Microtech, Thermo Fisher Scientific). Then, an immunohistochemical analysis was performed using standard techniques with the Cellular and Tissue Imaging Core Facility of Nantes University (Micro-PICell). Left panel: hematoxylin, phloxin, safran (HPS) MCTS staining. Right panel: antigen retrieval was performed using Impath Retrieval Solution pH 6.0 (Impath, ref: 44,998) at 101 °C for 20 min. Slides were incubated with hydrogen peroxyde blocking solution (Thermo scientific, ref: TA-125-HP) for 10 min then, incubated with rabbit serum 1.5% (Vector Laboratories, ref: S-5000) for 20 min at room temperature. PD-L1 antibody (R&D systems, AF156) was used at 0.5 µg/ml for 1 h then, biotinylated rabbit anti-goat secondary antibody was used at 3.75 µg/ml (Vector Laboratories, ref BA-5000) for 30 min. Revelation was performed using Large Volume Streptavidin peroxidase (Thermo Scientific, ref: TS-125-HR) for 30 min and DAB (3,3′-Diaminobenzidine). Pictures were obtained using a NanoZoomer 2.0HT (Hamamatsu). [file 12931_2024_2791_MOESM2_ESM.jpg]

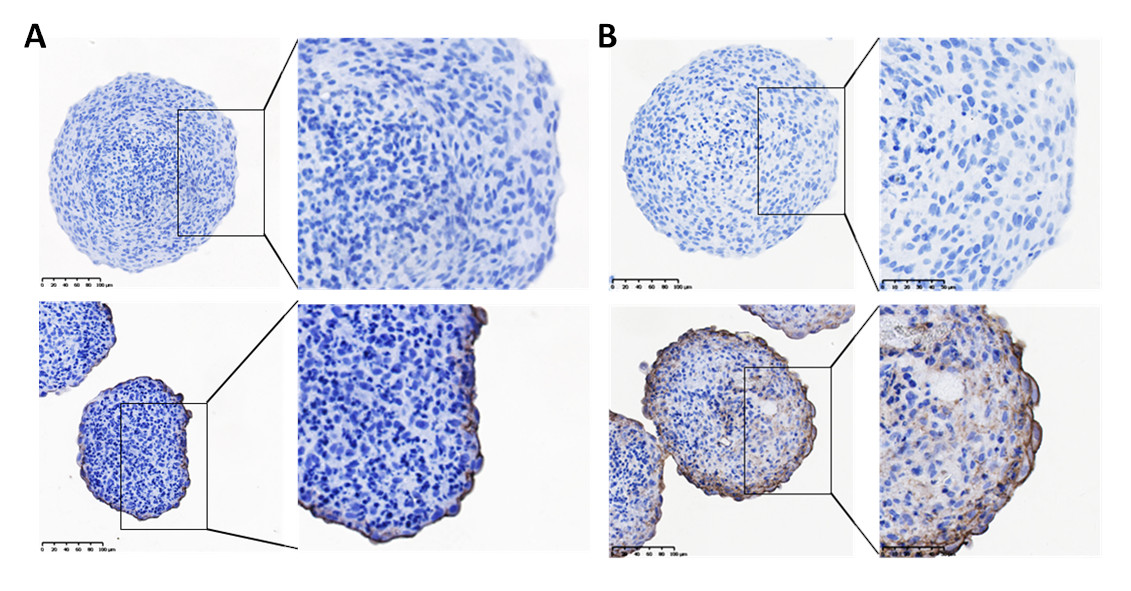

Supplement: Supplementary file 3 — Supplementary Figure S3: PD-L1 (CD274) expression is not induced in fibroblasts and macrophages following treatment. Lung cancer cells, ADCA117 (GFP +), fibroblasts (HFF-2) and monocytes were grown as MCTS. MCTS were treated with a combination of carboplatin 50µM and carboplatine 150 nM (CaPa) or gemcitabine 100nM (CaGe) repeated 3 times. PD-L1 expression was measured using flow cytometry after MCTS dissociation. PD-L1 expression analysis was performed on GFP – cells. [file 12931_2024_2791_MOESM3_ESM.jpg]

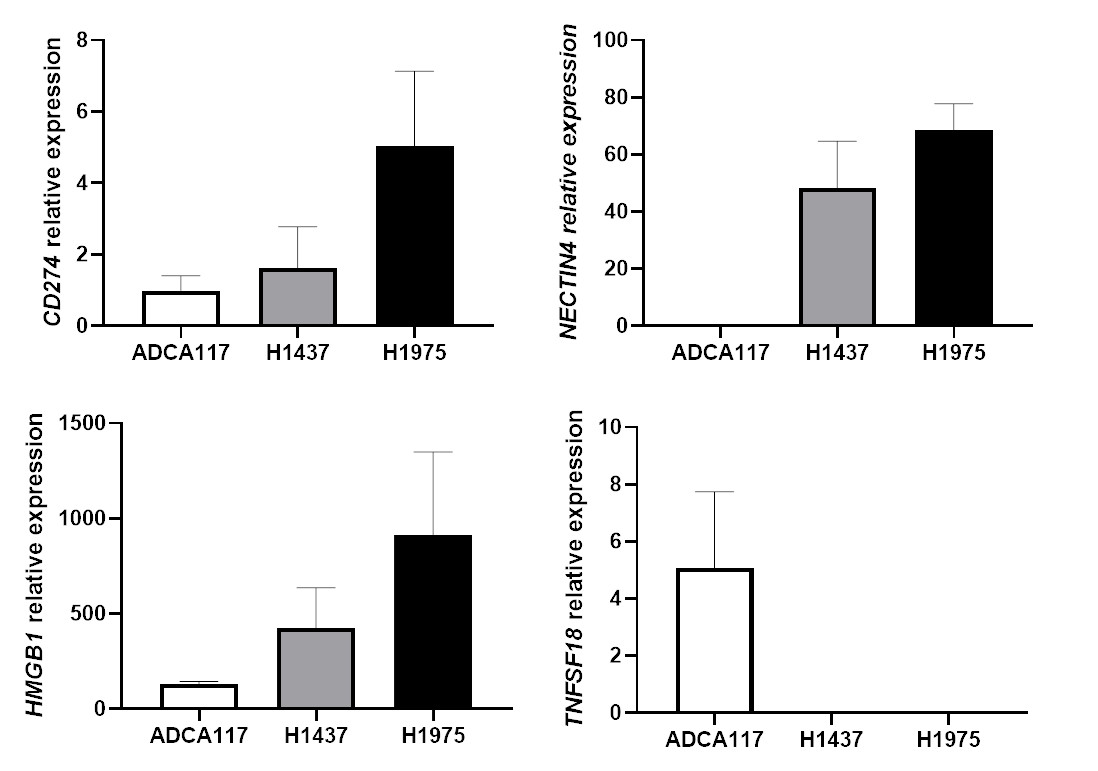

Supplement: Supplementary file 4 — Supplementary Figure S4:CD274, NECTIN4, HMGB1 and TNFSF18 genes expression in ADCA117, H1437 and H1975 cells. mRNA of lung cancer cells, ADCA117, H1437 and H1975 were extracted and genes expression was measured using real-time PCR. Results are expressed as the mean +/− SEM of three independent experiments. [file 12931_2024_2791_MOESM4_ESM.jpg]
