# Supplementary material for: Use of non-small cell lung cancer multicellular tumor spheroids to study the impact of chemotherapy
Source: Respir Res. 2024 Apr 5;25:156. doi: 10.1186/s12931-024-02791-5 (PMC10998296; doi:10.1186/s12931-024-02791-5)
Supplement: Supplementary file 5 — Supplementary Material 5 [file 12931_2024_2791_MOESM5_ESM.docx]

Table S1: Clinical information associated to cell lines used

| **Cell lines** | **Sex** | **Age** | **Diagnostic** | **Overall survival** | **Mutations** |
| --- | --- | --- | --- | --- | --- |
| ADCA 117 | M | 86 | NSCLC Adenocarcinoma  Pleural metastasis | 66 days | None |
| H1437 | M | 60 | NSCLC Adenocarcinoma  Pleural metastasis | / | MAP2K1 p.Q56P (p.Gln56Pro ; c.167A>C)  TP53 p.R267P (p.Arg267Pro ; c.800G>C) |
| H1975 | F | / | NSCLC Adenocarcinoma | / | EGFR p.T790M (p.Thr790Met ; c.2369C>T)  EGFR p.L858R (p.Leu858Arg ; c.2573T>G)  TP53 p.R273H (p.Arg273His ; c.818G>A) |

NSCLC, non-small cell lung cancer
