# Supplementary material for: Use of non-small cell lung cancer multicellular tumor spheroids to study the impact of chemotherapy
Source: Respir Res. 2024 Apr 5;25:156. doi: 10.1186/s12931-024-02791-5 (PMC10998296; doi:10.1186/s12931-024-02791-5)
Supplement: Supplementary file 6 — Supplementary Material 6 [file 12931_2024_2791_MOESM6_ESM.docx]

Table S4: List of the genes specifically regulated following CaPa treatment on ADCA117 MCTS.

| **Genes upregulated** | **Genes downregulated** |
| --- | --- |
| *TUBA4A*  *MRPL15*  *GABARAPL2*  *ARID5B*  *MT2A*  *HERC4*  *GABARAPL1*  *SLC11A2*  *AKR1B1*  *KPNA1*  *PRNP*  *SERPINB2*  *BTN2A1*  *PLPP3*  *TMCC3*  *INO80C*  *RTN1*  *TAF13*  *ISCU*  *STX1A*  *ASTN2*  *LINC01356*  *CCRL2*  *SERPINB4*  *LINC00632*  *INHBA*  *LAMB3*  *ATP1B1*  *TLCD5*  *TGM2*  *TMEM38B*  *CCL20*  *LURAP1L*  *FMN1*  *ADGRF4*  *CEBPB*  *LINC01128*  *RCAN1*  *LOC105378047*  *LINC02454*  *CDK17*  *VNN1*  *TRIM8*  *CAB39*  *NFKBIA*  *DRAM1*  *PTX3*  *MRGPRX3*  *ZNF79*  *MPP4*  *LINC02535*  *SLC7A7*  *ARHGEF2*  *UBA5*  *LOC101927151*  *DCBLD2*  *TRIM23*  *DCUN1D3*  *GDNF-AS1*  *NOP14-AS1*  *LACC1*  *LINC00342*  *MYO1B*  *EDN1*  *LOC105369201*  *YRDC*  *ATP6V0A1*  *EBI3*  *PTAFR* | *HCFC1R1*  *LGALS3*  *CUL4B*  *SULT1A1*  *HPRT1*  *MTHFD1*  *CEP250*  *HMGN2*  *THY1*  *S100A10*  *SMC1A*  *EMP2*  *EXOC4*  *IAH1*  *VRK1*  *NASP*  *ERLIN1*  *FARS2*  *TMEM237*  *DGLUCY*  *TCAF1*  *LOC100419583*  *PLAAT4*  *NFYB*  *MCM4*  *LAMB1*  *PSAT1*  *PLXDC2*  *CDKAL1*  *HHIPL2*  *UBR7*  *SLC39A11*  *CHEK1*  *NAV1*  *ATAD2*  *WDHD1*  *CDC25B*  *ZNF766*  *CCDC14*  *KIF11*  *ANKRD37*  *PLK4*  *JADE1*  *PCK2*  *CHAF1A*  *ALDH3B1*  *TRABD2A*  *CNTRL*  *POLE2*  *FOLR1*  *COL5A1*  *SBF2*  *NCAPG2*  *ZNF93*  *SYNE2*  *SCLT1*  *EFHC1*  *DCDC2*  *BLM*  *ID1*  *HIST1H4E*  *CCN3*  *PPP1R9A*  *CXCL14*  *PTPRM*  *MRC2*  *SLC27A5*  *POLA1*  *LINC01508*  *TNFSF4*  *USP1*  *ASB9*  *TRO*  *PHF19*  *LOXL1*  *CCDC152*  *CBR3*  *KIF14*  *SLC16A7* |
