# Supplementary material for: Use of non-small cell lung cancer multicellular tumor spheroids to study the impact of chemotherapy
Source: Respir Res. 2024 Apr 5;25:156. doi: 10.1186/s12931-024-02791-5 (PMC10998296; doi:10.1186/s12931-024-02791-5)
Supplement: Supplementary file 7 — Supplementary Material 7 [file 12931_2024_2791_MOESM7_ESM.docx]

Table S6: List of the genes commonly regulated following CaPa or CaGe treatment on ADCA117 MCTS.

| **Genes upregulated** | **Genes downregulated** |
| --- | --- |
| *TIMP1*  *SUGCT*  *CDKN1A*  *GDF15*  *C1QTNF1-AS1*  *DDIT3*  *ANKRD20A12P*  *MDM2*  *TIGAR*  *CDCP1*  *STC1*  *TFPI2*  *MMP3*  *IL24*  *CREM*  *PINCR*  *SP140*  *NECTIN4*  *PTGES*  *TMEM158*  *LOC102723769*  *CD274*  *SLC22A4*  *NAMPT*  *PTPRR*  *DHRS7*  *ABHD2*  *TM4SF1*  *AEN*  *HBEGF*  *RRM2B*  *STEAP1*  *CIDECP1*  *ST3GAL1*  *EHF*  *IL1A*  *SPHK1*  *LINC01588*  *CCND2*  *IL20*  *TNFSF15*  *ASB1*  *AK1*  *GADD45A*  *RPLP0P2*  *STOM*  *PPP1R14B-AS1*  *SMC2*  *TNIP3*  *SERPINB8*  *EMP1*  *YBX3*  *CSF3*  *FAS*  *DUSP6*  *LAMC2*  *LOC100288175*  *KYNU*  *TP53I3*  *AMPD3*  *SAT1*  *ANGPTL4*  *TSC22D1*  *IL1B*  *ZNF382*  *POU2F2*  *ITGA2*  *TM4SF19*  *PPP1R15A*  *AOX1*  *ATP2B1*  *TNFRSF10B*  *POPDC2*  *TMEM233*  *SERPIND1*  *PHLDA1*  *EIF5A2*  *ICAM1*  *VEPH1*  *PTPRN*  *NINJ1*  *PPP4R2*  *PPIF*  *LOC100505622*  *ZP3*  *LINC01704*  *NBEAP1*  *LINC01426*  *MPC2*  *HIST1H2BG*  *DLGAP1-AS2*  *TCERG1L-AS1*  *SLITRK4*  *PSG2*  *BEX2*  *ZC3H12A*  *RRAD*  *EDA2R*  *PI3*  *NKX3-1*  *GPX1*  *LOC100506178*  *NABP1*  *LINC01795*  *MAP3K7CL*  *LOC101927822*  *PAPPA*  *LIF*  *DPF3*  *UPP1*  *ALDH1A3*  *PNP*  *SLC43A3*  *IL6*  *DYRK3*  *FCMR*  *LETM2*  *PLAUR*  *ODC1*  *GDPD1*  *RBKS*  *BDKRB2*  *SESN2*  *TNIP1*  *CIDEC*  *LINC02015*  *SNHG1*  *GFPT2*  *SFTA1P*  *RSPO3*  *GDNF*  *IER3*  *C15orf48*  *INKA2*  *NDUFAF8*  *NRG1*  *TES*  *RND3*  *WFDC21P*  *SEC11C*  *EEF1AKMT4*  *SNHG15*  *PLAU*  *TPBG*  *CLDN1*  *TSPAN14*  *PVT1*  *OR8G2P*  *FMNL2*  *PMAIP1*  *SNX8*  *DYNC1H1*  *MAFF*  *BTG1*  *CXCL1*  *TNFRSF12A*  *VEGFA*  *CXCL8*  *LINC01173*  *HMGA2*  *CACNA2D4*  *CSF2*  *CCDC148*  *PCNX2*  *SOD2*  *USP53*  *GNG4*  *AFF1-AS1*  *C11orf91*  *ADIPOR1*  *ATP2C1*  *CXCL2*  *ETV4*  *BTG2*  *IL13RA2*  *RPL23AP7*  *RAB27B*  *PLK3*  *LOC110091776*  *ATP13A3*  *LOC541472*  *ATF3*  *INPP5D*  *LYPLAL1-DT*  *MTHFD2L*  *CXCL3*  *SLC25A37*  *PID1*  *MARCHF3*  *C3*  *SLCO4A1*  *LINC-PINT*  *G0S2*  *PSTPIP2*  *TMEM30A*  *FGF2*  *DEPDC7*  *LINC01537*  *ODAPH*  *GPR87*  *SLC16A6*  *RDH10* | *CCNB1*  *CENPF*  *NEK6*  *PLAC8*  *PRSS23*  *PRC1*  *BIRC5*  *CDCA3*  *SH2D4A*  *SLC12A8*  *TOP2A*  *CCNB2*  *CDCA8*  *CDKN3*  *LINC01638*  *KIF23*  *RPL22L1*  *MAD2L1*  *HJURP*  *EVI2A*  *HNRNPA1*  *SBF2-AS1*  *KRT8*  *PBK*  *DLGAP5*  *RARRES2*  *TMSB4X*  *HTR2B*  *KPNA2*  *GACAT2*  *PMP22*  *AURKA*  *AURKB*  *RHNO1*  *PTMA*  *TROAP*  *KIF20A*  *ZWINT*  *PRR11*  *RACGAP1*  *KIF4A*  *NCAPH*  *STMN1*  *CKAP2*  *HIST1H2AH*  *SPC25*  *HMMR*  *CENPN*  *SMYD3*  *UBE2C*  *AXL*  *ANLN*  *PCLAF*  *NUSAP1*  *HIST1H4C*  *CLSPN*  *NDC80*  *PLK1*  *CYB5A*  *HIST1H1B*  *BUB1*  *CDK1*  *ARL6IP1*  *ACTG1*  *SPDL1*  *PHYHD1*  *HMGB2*  *COL1A1*  *BTN3A2*  *C7orf69*  *EP400P1*  *NUPR1*  *H2AFZ*  *PDE1C*  *CDC20*  *MKI67*  *PRIM1*  *GINS2*  *THBS1*  *MATN2*  *PIR*  *SELENBP1*  *UTRN*  *S100A3*  *CENPW*  *RGS4*  *RAD51AP1*  *CFDP1*  *HSD17B11*  *BUB1B*  *CLU*  *CEP70*  *KIF2C*  *PARPBP*  *SPAG5*  *HIRIP3*  *SEPTIN6*  *OIP5-AS1*  *TK1*  *CAVIN2*  *COL1A2*  *GPNMB*  *TPX2*  *HIST1H1D*  *HMGB3*  *SGO2*  *CKAP2L*  *KNL1*  *FOXM1*  *RRM1*  *GTSE1*  *SERPINF1*  *LINC01224*  *MND1*  *BCHE*  *POC1A*  *NUF2*  *LINC01583*  *H2AFV*  *SLFN11*  *ESCO2*  *LMO7*  *HELLS*  *RFC4*  *FEN1*  *LOC107985911*  *PRKCA*  *HIST1H3B*  *HYLS1*  *TTK*  *GFRA1*  *UBA7*  *RGS5*  *MCM7*  *MCM3*  *MYH10*  *LRR1*  *PPP2R2B*  *PHGDH*  *KIF20B*  *CDCA7L*  *FHOD1*  *AOPEP*  *CKS1B*  *PLEKHA4*  *TMSB15B*  *CHRDL1*  *DLEU2*  *CLSTN2*  *ASPM*  *MBNL2*  *ANG*  *UBE2T*  *LOC102724927*  *ENO3*  *TMEM130*  *ANP32E*  *SKA3*  *CENPE*  *CCNA2*  *HMGN3*  *TRAIP*  *MELK*  *PLSCR4*  *TMEM14A*  *FANCI*  *FBXO32*  *PARP9*  *NCAPD2*  *SCAPER*  *COL8A1*  *SLC39A10*  *CENPM*  *CENPK*  *TMEM106C*  *SSBP2*  *RFC5*  *HIST1H1A*  *HLTF*  *WDR76*  *GALNT14*  *HADH*  *NTRK3*  *DIAPH3*  *FAM111B*  *COL12A1*  *SMC4*  *BRCA1*  *HMGN5*  *SGO1*  *C1orf21*  *CAPSL*  *KIFC1*  *CPQ*  *PIEZO2*  *TENM2*  *THBS3*  *TNS3*  *SYT1*  *CCDC80*  *CDC6*  *ANGPTL2*  *ZC4H2*  *DIAPH2*  *PTTG1*  *NREP*  *HAPLN1*  *MYL9*  *ALDH7A1*  *NEK2*  *ACTR3C*  *ADGRG6*  *IFITM1*  *TLN2*  *FANCD2*  *CPA4*  *NRM*  *RFC3*  *COL5A2*  *HIST1H1E*  *OXTR*  *PPL*  *ECSCR*  *GMNN*  *APOBEC3B*  *KLHL4*  *FLG*  *CDC45*  *CIT*  *RNASEH2A*  *MCM5*  *ID2*  *IFI44*  *C1R*  *RAD51B*  *COL4A2*  *HLA-F*  *FKBP7*  *CEP152*  *IMMP2L*  *TMSB15A*  *SOX4*  *LIPC*  *KRT80*  *HIST1H3C*  *ANXA3*  *MEST*  *GINS4*  *SHCBP1*  *SKA1*  *CDC25C*  *CHN1*  *KIF18A*  *ORC1*  *GINS1*  *MMP2*  *SKP2*  *MCM10*  *AIG1*  *ARHGAP11A*  *CIP2A*  *SPAG1*  *SLC37A4*  *PHKB*  *TMPO*  *CCNF*  *ARNT2*  *SNX7*  *GEMIN2*  *DEPDC1*  *AR*  *GALNT7*  *OXCT1*  *MAPRE2*  *TNNC1*  *LINC00592*  *RAD54B*  *MTFR2*  *NRXN3*  *UHRF1*  *ADIRF*  *DEPDC1B*  *KIF15*  *MPHOSPH9*  *RDM1* |
