# Supplementary material for: Use of non-small cell lung cancer multicellular tumor spheroids to study the impact of chemotherapy
Source: Respir Res. 2024 Apr 5;25:156. doi: 10.1186/s12931-024-02791-5 (PMC10998296; doi:10.1186/s12931-024-02791-5)
Supplement: Supplementary file 8 — Supplementary Material 8 [file 12931_2024_2791_MOESM8_ESM.docx]

Table S5: List of the genes specifically regulated following CaGe treatment on ADCA117 MCTS.

| **Genes upregulated** | **Genes downregulated** |
| --- | --- |
| *TRIAP1*  *SRA1*  *ZFAND2A*  *HIST1H2BD*  *BLOC1S2*  *BDNF*  *DDB2*  *SERPINE1*  *P4HA2*  *HIST1H2AE*  *NGRN*  *HDAC9*  *GABPB2*  *PDP1*  *NIBAN2*  *CASK*  *FHL2*  *LINC00941*  *RPL23AP82*  *POLH*  *TNFRSF10A*  *VSIG1*  *ARL14*  *MAFIP*  *LOC105371849*  *CLMP*  *BDKRB1*  *ZNF563*  *LOC105371267*  *RPL23AP53*  *DLC1*  *HIST1H2AC*  *FDXR*  *PLTP*  *TNFRSF10D*  *KRT18P55*  *LINC00513*  *PANX1*  *ANKRD36B*  *C1QTNF1*  *NCEH1*  *RRN3*  *HYI*  *SERPINB7*  *ZNF66*  *CES2*  *IGFN1*  *FBXW7*  *TMEM132A*  *LOC101929705*  *BCL2L1*  *KCNJ2*  *LPXN*  *ANKRA2*  *IKBIP*  *NFKBIZ*  *RGS16*  *LINC01186*  *ITGA1*  *TRIB3*  *ABAT*  *CMBL*  *CKMT2-AS1*  *FICD*  *HMGA1*  *LOC101928303*  *LNCOG*  *IFT20*  *CDC42EP2*  *GNA15*  *LOC652276*  *MRAS*  *RALY-AS1*  *RTTN*  *TNFRSF11B*  *PHC2*  *HRAT17*  *UBAC2-AS1*  *IRAK2*  *ULBP2*  *AP1S3*  *DESI1*  *LOC105370203*  *MAP4K3-DT* | *CALD1*  *CD74*  *ARHGDIB*  *STX8*  *DAAM1*  *KNSTRN*  *LITAF*  *GBP2*  *TMEM97*  *POLD3*  *PGRMC2*  *GBP1*  *EXOSC9*  *EBP*  *WFDC3*  *LINP1*  *AKR1C3*  *IFIT2*  *MPP1*  *CRIM1*  *AK5*  *CDK5RAP2*  *LOC100129034*  *CEP57*  *CDH4*  *SAMHD1*  *PXMP4*  *NT5C3A*  *LINC01297*  *PPBP*  *TRIM16L*  *METTL7B*  *IFIT1*  *TRAM2*  *C2*  *TRIM16*  *CEP55*  *COL4A1*  *IL7R*  *RTP4*  *AP1M2*  *THBS2*  *CENPU*  *IFIT3*  *MAGI3*  *GAS2L3*  *RGS7*  *SHTN1*  *HLA-DRA*  *CEP85*  *MMAB*  *ARHGEF9*  *CXXC5*  *HLA-DRB1*  *CDCA5*  *CCN5*  *SNCA*  *TNFSF18*  *CEP128*  *C4orf46*  *SLC7A5*  *NCAPG*  *DOCK2*  *ADAMTS2*  *BARD1*  *PAEP*  *HLA-DMA*  *MX2*  *MYBL2*  *SPC24*  *SASS6*  *STMN3*  *TRIM59*  *CASP8AP2*  *OASL*  *LOC100128885*  *TRIM47*  *LOC101929460*  *EDIL3*  *LOC101929427*  *CRISPLD1*  *PSD3*  *IFI44L*  *NPTX1*  *LIPA*  *CENPQ*  *DEPP1*  *EME1*  *MCM6*  *AEBP1*  *APOC1*  *LINC02057*  *EPSTI1*  *CLEC2B*  *DLG3*  *CCBE1*  *AKR1C2*  *RNASE1*  *SLC7A4*  *MNDA*  *GBP4*  *NEK3*  *EHMT1*  *DDO*  *ADAM12*  *MX1*  *TCN2*  *PTPN6*  *OAS3*  *TYROBP*  *CEMIP*  *CRABP2*  *TCF19*  *DOP1B*  *CD52*  *XRCC2*  *PARP1*  *TTC28-AS1*  *SBSN*  *FGGY*  *SCG2*  *IL18BP*  *OAS1*  *NDRG2*  *ITGB4*  *BTN3A1*  *LOC730101*  *VGLL3*  *MYO5B*  *MKS1*  *LAMA5*  *SLF1* |
