# Supplementary material for: Use of non-small cell lung cancer multicellular tumor spheroids to study the impact of chemotherapy
Source: Respir Res. 2024 Apr 5;25:156. doi: 10.1186/s12931-024-02791-5 (PMC10998296; doi:10.1186/s12931-024-02791-5)
Supplement: Supplementary file 9 — Supplementary Material 9 [file 12931_2024_2791_MOESM9_ESM.docx]

Table S3: List of the genes regulated following CaGe treatment on ADCA117 MCTS. -1 < log2FC > 1, padj < 0.01

| **Genes** | **log2FoldChange** | **pvalue** | **padj** |
| --- | --- | --- | --- |
| *TIMP1* | 2.747110071 | 2.33E-113 | 2.59E-109 |
| *SUGCT* | 4.434224101 | 5.17E-57 | 2.88E-53 |
| *CDKN1A* | 2.554616417 | 6.78E-49 | 1.89E-45 |
| *GDF15* | 3.721504039 | 5.96E-49 | 1.89E-45 |
| *CCNB1* | -2.679469683 | 6.86E-39 | 1.53E-35 |
| *CENPF* | -2.807682422 | 6.26E-37 | 1.16E-33 |
| *C1QTNF1-AS1* | 3.520806647 | 4.48E-35 | 7.12E-32 |
| *NEK6* | -1.651804901 | 2.69E-34 | 3.74E-31 |
| *DDIT3* | 2.183258849 | 3.84E-34 | 4.75E-31 |
| *PLAC8* | -2.395262516 | 7.89E-34 | 8.78E-31 |
| *ANKRD20A12P* | 2.032480381 | 1.48E-32 | 1.50E-29 |
| *MDM2* | 2.330922786 | 8.40E-31 | 7.79E-28 |
| *TIGAR* | 1.811434279 | 8.68E-30 | 7.43E-27 |
| *CDCP1* | 3.048675682 | 2.33E-29 | 1.85E-26 |
| *PRSS23* | -2.720286017 | 6.64E-29 | 4.93E-26 |
| *STC1* | 2.593388944 | 1.57E-28 | 1.09E-25 |
| *PRC1* | -3.169335 | 2.64E-27 | 1.73E-24 |
| *BIRC5* | -3.384377705 | 5.56E-27 | 3.44E-24 |
| *CDCA3* | -3.025858381 | 1.62E-26 | 9.50E-24 |
| *TRIAP1* | 1.228701194 | 2.31E-26 | 1.28E-23 |
| *SH2D4A* | -1.951923996 | 5.86E-25 | 3.10E-22 |
| *SLC12A8* | -2.243747214 | 1.66E-24 | 8.40E-22 |
| *TFPI2* | 4.176868896 | 1.90E-24 | 9.20E-22 |
| *MMP3* | 6.49520028 | 4.43E-24 | 2.06E-21 |
| *IL24* | 5.393849001 | 8.07E-24 | 3.59E-21 |
| *TOP2A* | -3.62455365 | 3.41E-23 | 1.46E-20 |
| *CCNB2* | -2.802033677 | 5.49E-23 | 2.26E-20 |
| *CDCA8* | -3.518290632 | 1.05E-22 | 4.03E-20 |
| *CDKN3* | -2.719198839 | 1.05E-22 | 4.03E-20 |
| *CREM* | 1.91339985 | 1.58E-22 | 5.74E-20 |
| *PINCR* | 4.011931394 | 1.60E-22 | 5.74E-20 |
| *CALD1* | -1.032223393 | 1.66E-22 | 5.78E-20 |
| *SP140* | 2.187825195 | 1.86E-22 | 6.28E-20 |
| *LINC01638* | -3.857596068 | 2.13E-22 | 6.97E-20 |
| *NECTIN4* | 4.663462707 | 3.35E-22 | 1.07E-19 |
| *PTGES* | 4.143659456 | 4.06E-22 | 1.26E-19 |
| *KIF23* | -3.083094036 | 2.92E-21 | 8.56E-19 |
| *TMEM158* | 2.676043991 | 4.64E-21 | 1.32E-18 |
| *LOC102723769* | 1.786819597 | 1.47E-20 | 4.09E-18 |
| *CD274* | 2.873494799 | 1.61E-20 | 4.36E-18 |
| *SLC22A4* | 3.387104892 | 3.98E-20 | 1.05E-17 |
| *NAMPT* | 1.92379985 | 6.04E-20 | 1.56E-17 |
| *RPL22L1* | -1.637982218 | 7.11E-20 | 1.80E-17 |
| *PTPRR* | 1.534364143 | 8.39E-20 | 2.08E-17 |
| *MAD2L1* | -1.990486638 | 9.86E-20 | 2.39E-17 |
| *HJURP* | -3.397382846 | 1.37E-19 | 3.24E-17 |
| *EVI2A* | -1.90172147 | 2.15E-19 | 4.98E-17 |
| *HNRNPA1* | -1.126687992 | 4.56E-19 | 1.04E-16 |
| *SBF2-AS1* | -3.720632424 | 9.85E-19 | 2.19E-16 |
| *KRT8* | -1.596710685 | 1.74E-18 | 3.80E-16 |
| *DHRS7* | 1.212221625 | 2.39E-18 | 5.12E-16 |
| *PBK* | -2.850740469 | 3.20E-18 | 6.73E-16 |
| *DLGAP5* | -3.734096342 | 1.85E-17 | 3.82E-15 |
| *RARRES2* | -3.43172497 | 2.03E-17 | 4.10E-15 |
| *TMSB4X* | -1.343097413 | 3.91E-17 | 7.77E-15 |
| *HTR2B* | -3.448943971 | 4.13E-17 | 8.06E-15 |
| *KPNA2* | -1.033313793 | 5.04E-17 | 9.68E-15 |
| *ABHD2* | 1.615408433 | 5.82E-17 | 1.10E-14 |
| *TM4SF1* | 1.219559468 | 5.91E-17 | 1.10E-14 |
| *GACAT2* | -2.729649026 | 6.06E-17 | 1.11E-14 |
| *AEN* | 1.623471676 | 6.36E-17 | 1.12E-14 |
| *HBEGF* | 3.863959947 | 6.34E-17 | 1.12E-14 |
| *PMP22* | -1.894985358 | 7.64E-17 | 1.33E-14 |
| *AURKA* | -1.638266947 | 8.83E-17 | 1.51E-14 |
| *AURKB* | -2.475434843 | 1.03E-16 | 1.74E-14 |
| *RHNO1* | -1.439751822 | 1.40E-16 | 2.33E-14 |
| *PTMA* | -1.095590956 | 1.61E-16 | 2.60E-14 |
| *TROAP* | -3.251534783 | 2.18E-16 | 3.47E-14 |
| *KIF20A* | -4.242029429 | 2.42E-16 | 3.80E-14 |
| *RRM2B* | 1.787671722 | 3.57E-16 | 5.45E-14 |
| *STEAP1* | 3.708123883 | 5.78E-16 | 8.69E-14 |
| *SRA1* | 1.025793909 | 6.33E-16 | 9.40E-14 |
| *CD74* | -1.815011237 | 8.66E-16 | 1.27E-13 |
| *ZWINT* | -1.767856314 | 9.51E-16 | 1.37E-13 |
| *CIDECP1* | 1.475720513 | 1.02E-15 | 1.46E-13 |
| *PRR11* | -1.891504718 | 1.06E-15 | 1.49E-13 |
| *ST3GAL1* | 1.626233325 | 1.33E-15 | 1.85E-13 |
| *EHF* | 5.058516493 | 1.42E-15 | 1.96E-13 |
| *IL1A* | 3.170671653 | 1.45E-15 | 1.96E-13 |
| *ARHGDIB* | -2.411943345 | 1.54E-15 | 2.07E-13 |
| *ZFAND2A* | 1.015474425 | 1.95E-15 | 2.58E-13 |
| *RACGAP1* | -2.369158378 | 3.17E-15 | 4.16E-13 |
| *SPHK1* | 1.493912037 | 3.61E-15 | 4.67E-13 |
| *KIF4A* | -4.247102499 | 4.27E-15 | 5.47E-13 |
| *NCAPH* | -3.095608163 | 5.49E-15 | 6.95E-13 |
| *STMN1* | -1.531275451 | 6.41E-15 | 8.02E-13 |
| *CKAP2* | -1.623521781 | 6.86E-15 | 8.49E-13 |
| *LINC01588* | 4.393126687 | 7.03E-15 | 8.60E-13 |
| *CCND2* | 2.672603007 | 9.91E-15 | 1.18E-12 |
| *HIST1H2AH* | -3.026290071 | 9.76E-15 | 1.18E-12 |
| *SPC25* | -2.919727288 | 9.94E-15 | 1.18E-12 |
| *IL20* | 5.258757364 | 1.38E-14 | 1.62E-12 |
| *HMMR* | -2.371144663 | 1.57E-14 | 1.82E-12 |
| *CENPN* | -1.832794405 | 1.95E-14 | 2.21E-12 |
| *STX8* | -1.093504027 | 2.63E-14 | 2.94E-12 |
| *SMYD3* | -1.549228681 | 2.84E-14 | 3.13E-12 |
| *TNFSF15* | 3.589110936 | 3.38E-14 | 3.69E-12 |
| *ASB1* | 2.159961795 | 4.14E-14 | 4.43E-12 |
| *UBE2C* | -2.536535956 | 4.14E-14 | 4.43E-12 |
| *AK1* | 1.182371618 | 4.27E-14 | 4.52E-12 |
| *AXL* | -1.33684199 | 4.66E-14 | 4.80E-12 |
| *HIST1H2BD* | 1.090504622 | 5.45E-14 | 5.56E-12 |
| *GADD45A* | 2.092068814 | 5.59E-14 | 5.66E-12 |
| *ANLN* | -2.443080815 | 5.87E-14 | 5.89E-12 |
| *PCLAF* | -1.949249326 | 7.33E-14 | 7.28E-12 |
| *RPLP0P2* | 2.330473904 | 9.14E-14 | 8.95E-12 |
| *STOM* | 1.948177251 | 9.17E-14 | 8.95E-12 |
| *PPP1R14B-AS1* | 1.58658929 | 1.07E-13 | 1.03E-11 |
| *SMC2* | 1.417008068 | 1.83E-13 | 1.74E-11 |
| *NUSAP1* | -2.467389794 | 1.92E-13 | 1.81E-11 |
| *TNIP3* | 2.356683593 | 2.25E-13 | 2.10E-11 |
| *HIST1H4C* | -1.789156738 | 2.32E-13 | 2.15E-11 |
| *SERPINB8* | 1.662473472 | 2.35E-13 | 2.16E-11 |
| *CLSPN* | -2.076740774 | 2.38E-13 | 2.17E-11 |
| *EMP1* | 2.011258617 | 2.48E-13 | 2.24E-11 |
| *NDC80* | -2.444752156 | 2.52E-13 | 2.26E-11 |
| *PLK1* | -2.090148491 | 3.05E-13 | 2.72E-11 |
| *YBX3* | 1.500428154 | 3.54E-13 | 3.12E-11 |
| *CSF3* | 3.956852519 | 4.19E-13 | 3.67E-11 |
| *CYB5A* | -1.443263489 | 5.52E-13 | 4.80E-11 |
| *HIST1H1B* | -4.263487041 | 6.12E-13 | 5.28E-11 |
| *BUB1* | -2.725048079 | 6.83E-13 | 5.85E-11 |
| *BLOC1S2* | 1.134671952 | 1.44E-12 | 1.21E-10 |
| *CDK1* | -2.256246312 | 1.63E-12 | 1.37E-10 |
| *ARL6IP1* | -1.176201838 | 2.37E-12 | 1.94E-10 |
| *FAS* | 2.088288889 | 2.41E-12 | 1.96E-10 |
| *ACTG1* | -1.135450235 | 2.54E-12 | 2.05E-10 |
| *SPDL1* | -1.849550305 | 2.62E-12 | 2.10E-10 |
| *PHYHD1* | -1.243212369 | 2.75E-12 | 2.19E-10 |
| *HMGB2* | -2.506647928 | 3.06E-12 | 2.42E-10 |
| *DUSP6* | 2.522887671 | 3.73E-12 | 2.91E-10 |
| *COL1A1* | -1.926163042 | 3.76E-12 | 2.91E-10 |
| *LAMC2* | 1.614897215 | 3.95E-12 | 3.04E-10 |
| *BTN3A2* | -1.181986606 | 4.16E-12 | 3.17E-10 |
| *C7orf69* | -3.334392112 | 4.20E-12 | 3.18E-10 |
| *EP400P1* | -2.093745818 | 4.63E-12 | 3.46E-10 |
| *NUPR1* | -1.878856196 | 4.78E-12 | 3.55E-10 |
| *DAAM1* | -1.124543184 | 5.00E-12 | 3.69E-10 |
| *LOC100288175* | 2.011130193 | 5.11E-12 | 3.74E-10 |
| *KYNU* | 2.585062393 | 8.43E-12 | 6.13E-10 |
| *H2AFZ* | -1.147083371 | 8.64E-12 | 6.25E-10 |
| *PDE1C* | -2.668352874 | 1.09E-11 | 7.79E-10 |
| *CDC20* | -3.281795503 | 1.30E-11 | 9.20E-10 |
| *MKI67* | -2.754099212 | 1.49E-11 | 1.05E-09 |
| *TP53I3* | 1.385546131 | 1.51E-11 | 1.06E-09 |
| *PRIM1* | -2.11975637 | 1.90E-11 | 1.32E-09 |
| *AMPD3* | 2.339520842 | 2.12E-11 | 1.47E-09 |
| *GINS2* | -2.464772421 | 3.50E-11 | 2.39E-09 |
| *THBS1* | -2.020823071 | 3.69E-11 | 2.51E-09 |
| *MATN2* | -1.752456944 | 3.82E-11 | 2.56E-09 |
| *SAT1* | 1.27944075 | 3.81E-11 | 2.56E-09 |
| *PIR* | -1.352902239 | 3.96E-11 | 2.64E-09 |
| *SELENBP1* | -1.389616724 | 4.09E-11 | 2.69E-09 |
| *UTRN* | -2.177773516 | 4.95E-11 | 3.23E-09 |
| *S100A3* | -1.667461465 | 5.36E-11 | 3.47E-09 |
| *CENPW* | -1.491930612 | 7.98E-11 | 5.14E-09 |
| *BDNF* | 1.129561806 | 9.19E-11 | 5.88E-09 |
| *RGS4* | -2.078065056 | 1.11E-10 | 7.08E-09 |
| *DDB2* | 1.328715506 | 1.13E-10 | 7.11E-09 |
| *KNSTRN* | -1.023686788 | 1.13E-10 | 7.11E-09 |
| *ANGPTL4* | 4.684951984 | 1.31E-10 | 8.17E-09 |
| *RAD51AP1* | -1.631991984 | 1.51E-10 | 9.35E-09 |
| *TSC22D1* | 1.441631067 | 1.71E-10 | 1.05E-08 |
| *IL1B* | 2.99721152 | 2.24E-10 | 1.36E-08 |
| *ZNF382* | 1.083912803 | 2.45E-10 | 1.48E-08 |
| *CFDP1* | -1.054789162 | 3.05E-10 | 1.82E-08 |
| *HSD17B11* | -1.558446244 | 3.59E-10 | 2.12E-08 |
| *POU2F2* | 1.584179648 | 3.60E-10 | 2.12E-08 |
| *LITAF* | -1.077989738 | 3.76E-10 | 2.21E-08 |
| *BUB1B* | -2.897530334 | 3.90E-10 | 2.26E-08 |
| *CLU* | -1.657458823 | 4.06E-10 | 2.34E-08 |
| *CEP70* | -1.784730375 | 4.27E-10 | 2.45E-08 |
| *ITGA2* | 1.995779345 | 4.54E-10 | 2.59E-08 |
| *KIF2C* | -2.295119116 | 5.34E-10 | 3.03E-08 |
| *PARPBP* | -1.965977593 | 5.35E-10 | 3.03E-08 |
| *TM4SF19* | 1.464476485 | 6.81E-10 | 3.83E-08 |
| *SPAG5* | -2.949352448 | 6.88E-10 | 3.85E-08 |
| *HIRIP3* | -1.890999413 | 7.30E-10 | 4.06E-08 |
| *SEPTIN6* | -1.337738496 | 7.67E-10 | 4.25E-08 |
| *OIP5-AS1* | -1.275166273 | 9.14E-10 | 5.03E-08 |
| *PPP1R15A* | 1.471632089 | 9.18E-10 | 5.03E-08 |
| *AOX1* | 2.604271582 | 1.02E-09 | 5.50E-08 |
| *ATP2B1* | 1.579325724 | 1.02E-09 | 5.50E-08 |
| *GBP2* | -2.293964238 | 1.02E-09 | 5.50E-08 |
| *TNFRSF10B* | 1.749265398 | 1.06E-09 | 5.67E-08 |
| *POPDC2* | 3.808094572 | 1.14E-09 | 6.07E-08 |
| *TK1* | -1.74347174 | 1.18E-09 | 6.24E-08 |
| *TMEM233* | 1.524475916 | 1.31E-09 | 6.90E-08 |
| *CAVIN2* | -1.694134876 | 1.37E-09 | 7.20E-08 |
| *SERPIND1* | 3.300665404 | 1.39E-09 | 7.26E-08 |
| *COL1A2* | -1.455840852 | 1.53E-09 | 7.97E-08 |
| *PHLDA1* | 1.44326558 | 1.76E-09 | 9.09E-08 |
| *SERPINE1* | 1.281653323 | 1.76E-09 | 9.09E-08 |
| *EIF5A2* | 1.32981647 | 1.84E-09 | 9.38E-08 |
| *GPNMB* | -1.712687332 | 1.83E-09 | 9.38E-08 |
| *ICAM1* | 1.510030549 | 1.89E-09 | 9.54E-08 |
| *TPX2* | -2.179393066 | 2.03E-09 | 1.02E-07 |
| *P4HA2* | 1.122145582 | 2.13E-09 | 1.07E-07 |
| *HIST1H1D* | -3.889665554 | 2.16E-09 | 1.08E-07 |
| *HMGB3* | -1.036928392 | 2.60E-09 | 1.28E-07 |
| *VEPH1* | 1.172089798 | 2.64E-09 | 1.30E-07 |
| *TMEM97* | -1.396064269 | 2.67E-09 | 1.30E-07 |
| *SGO2* | -1.856227963 | 3.26E-09 | 1.59E-07 |
| *CKAP2L* | -1.821733189 | 3.77E-09 | 1.80E-07 |
| *PTPRN* | 2.881834631 | 4.19E-09 | 1.98E-07 |
| *KNL1* | -2.922876132 | 4.50E-09 | 2.12E-07 |
| *NINJ1* | 1.273757292 | 4.88E-09 | 2.26E-07 |
| *FOXM1* | -2.556454709 | 5.06E-09 | 2.34E-07 |
| *PPP4R2* | 1.449242975 | 5.18E-09 | 2.38E-07 |
| *RRM1* | -1.113164249 | 5.38E-09 | 2.44E-07 |
| *PPIF* | 1.327175235 | 5.53E-09 | 2.49E-07 |
| *GTSE1* | -2.700645359 | 6.35E-09 | 2.85E-07 |
| *LOC100505622* | 1.619667503 | 7.42E-09 | 3.29E-07 |
| *SERPINF1* | -1.559207724 | 7.73E-09 | 3.40E-07 |
| *POLD3* | -1.026777453 | 8.54E-09 | 3.74E-07 |
| *LINC01224* | -2.81115939 | 8.62E-09 | 3.75E-07 |
| *MND1* | -2.288139376 | 8.61E-09 | 3.75E-07 |
| *BCHE* | -4.364928916 | 9.66E-09 | 4.18E-07 |
| *POC1A* | -2.003771225 | 1.02E-08 | 4.39E-07 |
| *NUF2* | -3.074926938 | 1.05E-08 | 4.51E-07 |
| *ZP3* | 2.454023665 | 1.06E-08 | 4.52E-07 |
| *LINC01704* | 4.557350915 | 1.07E-08 | 4.54E-07 |
| *LINC01583* | -1.989301706 | 1.12E-08 | 4.74E-07 |
| *NBEAP1* | 4.626879938 | 1.17E-08 | 4.91E-07 |
| *H2AFV* | -1.012945716 | 1.29E-08 | 5.41E-07 |
| *SLFN11* | -1.298429408 | 1.34E-08 | 5.60E-07 |
| *LINC01426* | 1.450755229 | 1.37E-08 | 5.67E-07 |
| *ESCO2* | -2.134512878 | 1.47E-08 | 6.04E-07 |
| *MPC2* | 1.004760534 | 1.53E-08 | 6.27E-07 |
| *LMO7* | -2.106961333 | 1.58E-08 | 6.44E-07 |
| *PGRMC2* | -1.47838368 | 1.69E-08 | 6.83E-07 |
| *HIST1H2BG* | 2.274267035 | 1.76E-08 | 7.08E-07 |
| *HELLS* | -2.066743069 | 1.87E-08 | 7.48E-07 |
| *RFC4* | -1.259823045 | 1.92E-08 | 7.67E-07 |
| *FEN1* | -1.340270354 | 1.93E-08 | 7.67E-07 |
| *DLGAP1-AS2* | 1.810420489 | 1.95E-08 | 7.74E-07 |
| *LOC107985911* | -1.219175111 | 2.16E-08 | 8.50E-07 |
| *PRKCA* | -2.040860924 | 2.48E-08 | 9.69E-07 |
| *HIST1H3B* | -3.274603874 | 3.04E-08 | 1.18E-06 |
| *TCERG1L-AS1* | 3.121013016 | 3.25E-08 | 1.25E-06 |
| *SLITRK4* | 2.230384249 | 3.44E-08 | 1.33E-06 |
| *HYLS1* | -1.295952379 | 3.52E-08 | 1.35E-06 |
| *TTK* | -3.333715821 | 3.55E-08 | 1.35E-06 |
| *PSG2* | 1.23621794 | 3.61E-08 | 1.37E-06 |
| *GBP1* | -1.580690872 | 3.66E-08 | 1.38E-06 |
| *GFRA1* | -2.852321916 | 3.66E-08 | 1.38E-06 |
| *BEX2* | 1.763278349 | 4.01E-08 | 1.50E-06 |
| *UBA7* | -1.680077545 | 4.16E-08 | 1.55E-06 |
| *RGS5* | -3.395231608 | 4.25E-08 | 1.58E-06 |
| *MCM7* | -1.657959336 | 4.42E-08 | 1.63E-06 |
| *ZC3H12A* | 1.762584044 | 4.60E-08 | 1.68E-06 |
| *HIST1H2AE* | 1.154938106 | 4.63E-08 | 1.69E-06 |
| *MCM3* | -1.255850646 | 4.71E-08 | 1.71E-06 |
| *MYH10* | -1.882441654 | 4.97E-08 | 1.80E-06 |
| *RRAD* | 2.285886965 | 5.17E-08 | 1.86E-06 |
| *LRR1* | -1.146394078 | 5.19E-08 | 1.86E-06 |
| *PPP2R2B* | -4.303770045 | 5.22E-08 | 1.87E-06 |
| *EDA2R* | 1.477920095 | 5.61E-08 | 2.00E-06 |
| *EXOSC9* | -1.020788708 | 5.70E-08 | 2.02E-06 |
| *NGRN* | 1.048927882 | 5.78E-08 | 2.04E-06 |
| *PHGDH* | -1.54381464 | 5.84E-08 | 2.06E-06 |
| *KIF20B* | -1.793348504 | 6.19E-08 | 2.17E-06 |
| *PI3* | 3.339294928 | 6.53E-08 | 2.27E-06 |
| *NKX3-1* | 1.648956278 | 6.88E-08 | 2.37E-06 |
| *EBP* | -1.04208889 | 7.35E-08 | 2.52E-06 |
| *WFDC3* | -1.325206381 | 7.98E-08 | 2.73E-06 |
| *GPX1* | 1.352659834 | 8.11E-08 | 2.77E-06 |
| *CDCA7L* | -1.45402461 | 8.26E-08 | 2.80E-06 |
| *LOC100506178* | 3.772653355 | 9.06E-08 | 3.06E-06 |
| *NABP1* | 1.042074403 | 9.07E-08 | 3.06E-06 |
| *FHOD1* | -1.165344941 | 9.56E-08 | 3.19E-06 |
| *AOPEP* | -1.208227598 | 1.03E-07 | 3.42E-06 |
| *CKS1B* | -1.156179047 | 1.09E-07 | 3.61E-06 |
| *PLEKHA4* | -1.666888003 | 1.11E-07 | 3.65E-06 |
| *HDAC9* | 1.575116238 | 1.14E-07 | 3.73E-06 |
| *LINC01795* | 4.801376746 | 1.14E-07 | 3.73E-06 |
| *MAP3K7CL* | 1.33647531 | 1.15E-07 | 3.75E-06 |
| *LOC101927822* | 4.081200953 | 1.20E-07 | 3.91E-06 |
| *TMSB15B* | -1.781138094 | 1.22E-07 | 3.95E-06 |
| *CHRDL1* | -1.325409822 | 1.30E-07 | 4.19E-06 |
| *DLEU2* | -3.072510899 | 1.33E-07 | 4.27E-06 |
| *CLSTN2* | -4.933333421 | 1.34E-07 | 4.29E-06 |
| *ASPM* | -3.834466515 | 1.34E-07 | 4.30E-06 |
| *MBNL2* | -1.356511044 | 1.41E-07 | 4.49E-06 |
| *PAPPA* | 2.899811647 | 1.50E-07 | 4.74E-06 |
| *ANG* | -3.184731492 | 1.58E-07 | 4.98E-06 |
| *LIF* | 2.563030437 | 1.59E-07 | 5.00E-06 |
| *UBE2T* | -1.344026533 | 1.61E-07 | 5.04E-06 |
| *DPF3* | 1.218172889 | 1.66E-07 | 5.17E-06 |
| *UPP1* | 1.313070372 | 1.67E-07 | 5.18E-06 |
| *LOC102724927* | -1.78478731 | 1.79E-07 | 5.52E-06 |
| *ALDH1A3* | 2.628834159 | 1.96E-07 | 5.98E-06 |
| *ENO3* | -1.242114555 | 1.98E-07 | 6.01E-06 |
| *PNP* | 1.070544528 | 1.98E-07 | 6.01E-06 |
| *TMEM130* | -3.70166151 | 2.00E-07 | 6.03E-06 |
| *SLC43A3* | 1.200398105 | 2.01E-07 | 6.03E-06 |
| *IL6* | 3.182193936 | 2.04E-07 | 6.13E-06 |
| *DYRK3* | 2.310069775 | 2.10E-07 | 6.28E-06 |
| *FCMR* | 3.032326531 | 2.13E-07 | 6.35E-06 |
| *LETM2* | 1.000713213 | 2.13E-07 | 6.35E-06 |
| *PLAUR* | 1.159913383 | 2.24E-07 | 6.60E-06 |
| *ANP32E* | -1.167877701 | 2.28E-07 | 6.72E-06 |
| *ODC1* | 1.493329601 | 2.34E-07 | 6.86E-06 |
| *SKA3* | -2.135121161 | 2.42E-07 | 7.09E-06 |
| *CENPE* | -2.06710657 | 2.44E-07 | 7.14E-06 |
| *GDPD1* | 2.365736485 | 2.59E-07 | 7.52E-06 |
| *RBKS* | 2.016810478 | 2.78E-07 | 8.04E-06 |
| *CCNA2* | -3.200599424 | 2.82E-07 | 8.11E-06 |
| *BDKRB2* | 2.85106797 | 2.85E-07 | 8.19E-06 |
| *HMGN3* | -1.158833213 | 3.23E-07 | 9.23E-06 |
| *TRAIP* | -1.72476723 | 3.82E-07 | 1.08E-05 |
| *SESN2* | 2.516490428 | 3.89E-07 | 1.10E-05 |
| *TNIP1* | 1.156837179 | 3.95E-07 | 1.11E-05 |
| *MELK* | -2.477679973 | 4.13E-07 | 1.16E-05 |
| *GABPB2* | 1.751815404 | 4.70E-07 | 1.29E-05 |
| *PLSCR4* | -1.551767907 | 4.69E-07 | 1.29E-05 |
| *TMEM14A* | -1.199959561 | 4.88E-07 | 1.34E-05 |
| *FANCI* | -1.939968228 | 5.06E-07 | 1.39E-05 |
| *CIDEC* | 1.898955618 | 5.33E-07 | 1.45E-05 |
| *FBXO32* | -1.751239046 | 5.36E-07 | 1.46E-05 |
| *LINC02015* | 2.277322426 | 5.47E-07 | 1.49E-05 |
| *PARP9* | -1.547028692 | 6.09E-07 | 1.63E-05 |
| *NCAPD2* | -1.632397686 | 6.48E-07 | 1.73E-05 |
| *SNHG1* | 2.393377814 | 6.62E-07 | 1.76E-05 |
| *LINP1* | -3.039136241 | 6.65E-07 | 1.76E-05 |
| *GFPT2* | 1.78805432 | 6.86E-07 | 1.80E-05 |
| *SFTA1P* | 1.4798702 | 7.00E-07 | 1.83E-05 |
| *RSPO3* | 3.688041354 | 7.24E-07 | 1.89E-05 |
| *AKR1C3* | -1.29299925 | 7.29E-07 | 1.90E-05 |
| *IFIT2* | -2.127260872 | 7.51E-07 | 1.95E-05 |
| *SCAPER* | -1.44469804 | 7.64E-07 | 1.97E-05 |
| *COL8A1* | -2.781923214 | 7.66E-07 | 1.97E-05 |
| *SLC39A10* | -1.193142769 | 8.79E-07 | 2.24E-05 |
| *GDNF* | 1.979835313 | 8.82E-07 | 2.25E-05 |
| *PDP1* | 1.089218364 | 8.95E-07 | 2.27E-05 |
| *CENPM* | -1.746634765 | 9.08E-07 | 2.30E-05 |
| *CENPK* | -1.475670668 | 9.12E-07 | 2.30E-05 |
| *TMEM106C* | -1.173369413 | 9.45E-07 | 2.38E-05 |
| *SSBP2* | -1.44376693 | 1.16E-06 | 2.89E-05 |
| *IER3* | 1.846165242 | 1.20E-06 | 2.97E-05 |
| *MPP1* | -2.812811465 | 1.42E-06 | 3.49E-05 |
| *RFC5* | -1.17326831 | 1.46E-06 | 3.57E-05 |
| *C15orf48* | 1.820964455 | 1.53E-06 | 3.71E-05 |
| *HIST1H1A* | -4.265302869 | 1.57E-06 | 3.78E-05 |
| *INKA2* | 2.393577111 | 1.56E-06 | 3.78E-05 |
| *NDUFAF8* | 1.237976389 | 1.57E-06 | 3.78E-05 |
| *HLTF* | -1.250720039 | 1.58E-06 | 3.79E-05 |
| *WDR76* | -2.052843636 | 1.59E-06 | 3.82E-05 |
| *GALNT14* | -2.737977611 | 1.59E-06 | 3.82E-05 |
| *HADH* | -1.603397018 | 1.75E-06 | 4.15E-05 |
| *NTRK3* | -1.752297031 | 1.76E-06 | 4.17E-05 |
| *NRG1* | 1.611008028 | 1.81E-06 | 4.27E-05 |
| *TES* | 1.869325031 | 1.82E-06 | 4.28E-05 |
| *RND3* | 1.069349262 | 1.82E-06 | 4.28E-05 |
| *CRIM1* | -1.000046673 | 1.86E-06 | 4.37E-05 |
| *AK5* | -2.123974617 | 2.08E-06 | 4.85E-05 |
| *NIBAN2* | 1.160226728 | 2.13E-06 | 4.94E-05 |
| *DIAPH3* | -2.50273379 | 2.14E-06 | 4.96E-05 |
| *CDK5RAP2* | -1.041272856 | 2.20E-06 | 5.09E-05 |
| *FAM111B* | -1.833322608 | 2.22E-06 | 5.12E-05 |
| *COL12A1* | -2.470127415 | 2.29E-06 | 5.26E-05 |
| *CASK* | 1.683129422 | 2.42E-06 | 5.55E-05 |
| *SMC4* | -1.371297191 | 2.52E-06 | 5.72E-05 |
| *BRCA1* | -1.8175368 | 2.58E-06 | 5.84E-05 |
| *WFDC21P* | 1.636291654 | 2.76E-06 | 6.20E-05 |
| *HMGN5* | -1.608950357 | 2.87E-06 | 6.44E-05 |
| *LOC100129034* | -2.442177528 | 2.87E-06 | 6.44E-05 |
| *SGO1* | -1.993387843 | 2.91E-06 | 6.51E-05 |
| *CEP57* | -1.516968975 | 3.11E-06 | 6.89E-05 |
| *C1orf21* | -1.478209543 | 3.14E-06 | 6.94E-05 |
| *FHL2* | 1.285956015 | 3.15E-06 | 6.95E-05 |
| *SEC11C* | 1.069995081 | 3.17E-06 | 6.99E-05 |
| *CAPSL* | -3.766337605 | 3.19E-06 | 7.03E-05 |
| *KIFC1* | -2.769637951 | 3.22E-06 | 7.07E-05 |
| *EEF1AKMT4* | 1.007152323 | 3.40E-06 | 7.40E-05 |
| *CDH4* | -4.659365647 | 3.46E-06 | 7.51E-05 |
| *SNHG15* | 1.383933097 | 3.69E-06 | 7.98E-05 |
| *SAMHD1* | -1.593635433 | 4.26E-06 | 9.11E-05 |
| *CPQ* | -3.152975634 | 4.48E-06 | 9.54E-05 |
| *PIEZO2* | -1.762213984 | 4.51E-06 | 9.59E-05 |
| *PLAU* | 1.290008298 | 4.53E-06 | 9.61E-05 |
| *PXMP4* | -1.042670687 | 4.59E-06 | 9.72E-05 |
| *NT5C3A* | -1.246337795 | 4.65E-06 | 9.82E-05 |
| *TENM2* | -2.938657056 | 4.76E-06 | 0.00010019 |
| *TPBG* | 1.652815097 | 4.84E-06 | 0.00010157 |
| *THBS3* | -1.566537144 | 5.02E-06 | 0.00010468 |
| *TNS3* | -1.087661724 | 5.05E-06 | 0.00010505 |
| *LINC01297* | -5.160421103 | 5.06E-06 | 0.00010517 |
| *SYT1* | -1.32196392 | 5.09E-06 | 0.00010527 |
| *LINC00941* | 1.853616383 | 5.12E-06 | 0.00010575 |
| *PPBP* | -5.165838914 | 5.16E-06 | 0.0001063 |
| *CLDN1* | 2.359040657 | 5.25E-06 | 0.00010773 |
| *CCDC80* | -1.261642135 | 5.57E-06 | 0.00011369 |
| *CDC6* | -1.437873767 | 5.58E-06 | 0.00011378 |
| *TRIM16L* | -1.653738404 | 5.91E-06 | 0.00011913 |
| *ANGPTL2* | -1.89912929 | 5.92E-06 | 0.00011918 |
| *RPL23AP82* | 1.048392924 | 6.13E-06 | 0.00012286 |
| *TSPAN14* | 1.398414462 | 6.16E-06 | 0.00012328 |
| *POLH* | 1.139830181 | 6.32E-06 | 0.00012629 |
| *PVT1* | 1.254320864 | 6.48E-06 | 0.00012911 |
| *ZC4H2* | -1.81816886 | 6.53E-06 | 0.0001298 |
| *DIAPH2* | -1.596492637 | 6.63E-06 | 0.00013136 |
| *PTTG1* | -1.132775139 | 6.63E-06 | 0.00013136 |
| *TNFRSF10A* | 2.050641123 | 6.77E-06 | 0.00013381 |
| *NREP* | -2.539014908 | 6.85E-06 | 0.00013511 |
| *METTL7B* | -4.233846479 | 7.07E-06 | 0.00013889 |
| *HAPLN1* | -5.828970051 | 7.27E-06 | 0.00014232 |
| *MYL9* | -1.776397785 | 7.42E-06 | 0.0001446 |
| *ALDH7A1* | -1.12657788 | 7.55E-06 | 0.00014693 |
| *VSIG1* | 4.555820476 | 7.56E-06 | 0.00014693 |
| *NEK2* | -2.273071123 | 7.67E-06 | 0.00014819 |
| *IFIT1* | -2.622183486 | 8.48E-06 | 0.00016326 |
| *TRAM2* | -1.329998231 | 8.89E-06 | 0.00017008 |
| *ACTR3C* | -1.574641049 | 8.95E-06 | 0.00017093 |
| *OR8G2P* | 2.960094889 | 9.16E-06 | 0.00017402 |
| *FMNL2* | 1.699074951 | 1.01E-05 | 0.00018967 |
| *PMAIP1* | 1.153860133 | 1.05E-05 | 0.00019778 |
| *ADGRG6* | -2.805000578 | 1.09E-05 | 0.0002046 |
| *IFITM1* | -2.229033795 | 1.11E-05 | 0.00020723 |
| *TLN2* | -1.17753628 | 1.13E-05 | 0.00021024 |
| *FANCD2* | -1.573638586 | 1.14E-05 | 0.00021303 |
| *ARL14* | 3.564825201 | 1.18E-05 | 0.00021897 |
| *CPA4* | -2.811647065 | 1.23E-05 | 0.00022817 |
| *NRM* | -1.007951643 | 1.24E-05 | 0.0002298 |
| *RFC3* | -1.720363371 | 1.25E-05 | 0.00023038 |
| *SNX8* | 1.02151657 | 1.26E-05 | 0.00023216 |
| *DYNC1H1* | 1.119170478 | 1.30E-05 | 0.00023972 |
| *MAFIP* | 1.053625807 | 1.33E-05 | 0.00024318 |
| *MAFF* | 2.312112278 | 1.38E-05 | 0.00025152 |
| *LOC105371849* | 2.958796219 | 1.38E-05 | 0.0002519 |
| *BTG1* | 1.226256076 | 1.41E-05 | 0.00025772 |
| *CXCL1* | 3.134991421 | 1.45E-05 | 0.00026328 |
| *TNFRSF12A* | 1.075604436 | 1.54E-05 | 0.00027851 |
| *CLMP* | 2.200068061 | 1.59E-05 | 0.00028622 |
| *COL5A2* | -1.752256721 | 1.66E-05 | 0.00029557 |
| *BDKRB1* | 2.218502148 | 1.71E-05 | 0.00030341 |
| *ZNF563* | 2.033927202 | 1.74E-05 | 0.00030863 |
| *HIST1H1E* | -1.991789334 | 1.82E-05 | 0.00032216 |
| *LOC105371267* | 1.734115911 | 1.83E-05 | 0.0003232 |
| *OXTR* | -2.107342966 | 1.85E-05 | 0.0003257 |
| *RPL23AP53* | 1.476546726 | 1.86E-05 | 0.00032696 |
| *DLC1* | 1.578218215 | 1.87E-05 | 0.00032814 |
| *PPL* | -1.640933606 | 1.88E-05 | 0.00032904 |
| *ECSCR* | -2.878211956 | 1.94E-05 | 0.00033824 |
| *HIST1H2AC* | 1.156358754 | 1.98E-05 | 0.00034451 |
| *C2* | -2.643865891 | 1.99E-05 | 0.00034485 |
| *FDXR* | 1.925389925 | 2.00E-05 | 0.00034522 |
| *PLTP* | 1.528345836 | 2.04E-05 | 0.00034959 |
| *TNFRSF10D* | 1.702576657 | 2.04E-05 | 0.00035077 |
| *GMNN* | -1.094688641 | 2.09E-05 | 0.00035662 |
| *APOBEC3B* | -2.288379361 | 2.21E-05 | 0.00037476 |
| *VEGFA* | 1.315939588 | 2.22E-05 | 0.00037698 |
| *TRIM16* | -1.148513589 | 2.23E-05 | 0.00037844 |
| *KLHL4* | -3.179621257 | 2.29E-05 | 0.00038566 |
| *CXCL8* | 2.522048024 | 2.30E-05 | 0.00038677 |
| *LINC01173* | 2.81710846 | 2.30E-05 | 0.00038677 |
| *HMGA2* | 3.27655309 | 2.35E-05 | 0.00039262 |
| *FLG* | -4.012052412 | 2.38E-05 | 0.00039832 |
| *CDC45* | -1.458354285 | 2.43E-05 | 0.00040476 |
| *CIT* | -3.169029132 | 2.54E-05 | 0.0004207 |
| *CEP55* | -2.714372369 | 2.56E-05 | 0.00042385 |
| *COL4A1* | -1.586295709 | 2.60E-05 | 0.00042746 |
| *CACNA2D4* | 1.627094308 | 2.63E-05 | 0.00043122 |
| *CSF2* | 2.888785984 | 2.68E-05 | 0.00043756 |
| *RNASEH2A* | -1.470771329 | 2.68E-05 | 0.00043759 |
| *CCDC148* | 1.714589387 | 2.74E-05 | 0.00044633 |
| *PCNX2* | 1.791962975 | 2.76E-05 | 0.0004492 |
| *KRT18P55* | 3.744819828 | 2.91E-05 | 0.00046989 |
| *MCM5* | -1.229402969 | 2.95E-05 | 0.00047549 |
| *SOD2* | 1.713365473 | 2.99E-05 | 0.00048129 |
| *LINC00513* | 1.924093524 | 3.00E-05 | 0.00048163 |
| *ID2* | -1.213785461 | 3.06E-05 | 0.00048998 |
| *IFI44* | -1.67811711 | 3.07E-05 | 0.00048998 |
| *PANX1* | 1.228884747 | 3.19E-05 | 0.00050651 |
| *USP53* | 1.230900575 | 3.22E-05 | 0.00051042 |
| *C1R* | -1.272026129 | 3.25E-05 | 0.00051331 |
| *RAD51B* | -2.354380718 | 3.56E-05 | 0.00055726 |
| *COL4A2* | -1.466659655 | 3.69E-05 | 0.00057543 |
| *IL7R* | -1.038749315 | 3.73E-05 | 0.00058063 |
| *ANKRD36B* | 1.461402685 | 3.84E-05 | 0.00059526 |
| *GNG4* | 1.543702182 | 3.85E-05 | 0.00059644 |
| *RTP4* | -1.48631158 | 3.90E-05 | 0.00060335 |
| *AP1M2* | -2.071653252 | 4.07E-05 | 0.00062772 |
| *AFF1-AS1* | 2.003727576 | 4.19E-05 | 0.00064262 |
| *HLA-F* | -1.36156131 | 4.37E-05 | 0.00066797 |
| *C1QTNF1* | 1.52568922 | 4.41E-05 | 0.00066946 |
| *THBS2* | -1.005452741 | 4.41E-05 | 0.00066946 |
| *C11orf91* | 1.991978615 | 4.49E-05 | 0.00068041 |
| *FKBP7* | -1.31339879 | 4.55E-05 | 0.0006875 |
| *CEP152* | -1.561081037 | 4.81E-05 | 0.00072535 |
| *CENPU* | -1.993124285 | 4.91E-05 | 0.00073957 |
| *IMMP2L* | -3.211703298 | 4.93E-05 | 0.00074142 |
| *IFIT3* | -1.686755251 | 5.00E-05 | 0.0007499 |
| *TMSB15A* | -2.924105315 | 5.27E-05 | 0.00078605 |
| *ADIPOR1* | 1.080204294 | 5.46E-05 | 0.00081323 |
| *MAGI3* | -3.935475607 | 5.61E-05 | 0.00083129 |
| *ATP2C1* | 1.283705748 | 5.63E-05 | 0.00083319 |
| *SOX4* | -1.42483825 | 5.65E-05 | 0.00083401 |
| *LIPC* | -3.744488502 | 5.74E-05 | 0.00084613 |
| *CXCL2* | 2.242045046 | 6.15E-05 | 0.00090334 |
| *KRT80* | -2.742857267 | 6.15E-05 | 0.00090334 |
| *NCEH1* | 1.035021183 | 6.14E-05 | 0.00090334 |
| *RRN3* | 1.1205331 | 6.26E-05 | 0.00091742 |
| *ETV4* | 1.645152694 | 6.31E-05 | 0.00092473 |
| *HIST1H3C* | -3.582763664 | 6.35E-05 | 0.00092852 |
| *HYI* | 1.226611468 | 6.52E-05 | 0.00095014 |
| *ANXA3* | -1.259743484 | 6.71E-05 | 0.00097574 |
| *GAS2L3* | -2.195278288 | 6.73E-05 | 0.00097704 |
| *RGS7* | -2.273341999 | 6.85E-05 | 0.00098861 |
| *SHTN1* | -1.087465056 | 6.90E-05 | 0.0009932 |
| *HLA-DRA* | -2.217439283 | 7.02E-05 | 0.0010083 |
| *SERPINB7* | 2.306300712 | 7.55E-05 | 0.0010747 |
| *CEP85* | -2.297439969 | 7.59E-05 | 0.00107752 |
| *MMAB* | -1.03214659 | 7.68E-05 | 0.0010868 |
| *ZNF66* | 1.14969329 | 7.75E-05 | 0.00109413 |
| *CES2* | 1.317095635 | 7.84E-05 | 0.00110637 |
| *MEST* | -2.984597495 | 7.90E-05 | 0.00111126 |
| *BTG2* | 2.565831833 | 7.92E-05 | 0.00111333 |
| *GINS4* | -1.21548142 | 8.00E-05 | 0.00112023 |
| *ARHGEF9* | -1.017131464 | 8.19E-05 | 0.00114424 |
| *CXXC5* | -1.603313363 | 8.41E-05 | 0.00117021 |
| *HLA-DRB1* | -1.92028259 | 8.45E-05 | 0.00117499 |
| *CDCA5* | -2.275417029 | 8.73E-05 | 0.00121024 |
| *CCN5* | -4.325441489 | 9.25E-05 | 0.00127742 |
| *IGFN1* | 1.31632911 | 9.51E-05 | 0.0013089 |
| *FBXW7* | 1.639532063 | 9.58E-05 | 0.00131666 |
| *SHCBP1* | -2.124241238 | 9.75E-05 | 0.00133763 |
| *SNCA* | -2.424138041 | 0.00010238 | 0.00140013 |
| *TNFSF18* | -2.838725216 | 0.00011488 | 0.00155766 |
| *SKA1* | -1.597607678 | 0.00011791 | 0.0015949 |
| *TMEM132A* | 1.804331086 | 0.00011826 | 0.00159601 |
| *LOC101929705* | 2.116965981 | 0.00011891 | 0.00160252 |
| *BCL2L1* | 1.230054276 | 0.00011927 | 0.00160542 |
| *CDC25C* | -2.423431744 | 0.00012183 | 0.00163394 |
| *CEP128* | -3.157056418 | 0.0001222 | 0.00163694 |
| *IL13RA2* | 2.858473104 | 0.00012444 | 0.00166216 |
| *KCNJ2* | 1.728275925 | 0.00012453 | 0.00166216 |
| *RPL23AP7* | 1.105147816 | 0.00013369 | 0.001776 |
| *LPXN* | 1.575574596 | 0.00013427 | 0.00177934 |
| *CHN1* | -2.20593702 | 0.00013634 | 0.00180039 |
| *KIF18A* | -1.453150458 | 0.00013652 | 0.00180069 |
| *C4orf46* | -1.728446579 | 0.00013867 | 0.00181821 |
| *ORC1* | -2.213606393 | 0.00014305 | 0.00186908 |
| *RAB27B* | 1.519080874 | 0.00014708 | 0.0019149 |
| *SLC7A5* | -1.239645356 | 0.0001487 | 0.00193381 |
| *GINS1* | -2.309731291 | 0.00014914 | 0.00193729 |
| *NCAPG* | -3.071646034 | 0.00015356 | 0.00198536 |
| *DOCK2* | -3.995726855 | 0.00015492 | 0.00199838 |
| *ANKRA2* | 1.438444947 | 0.00015597 | 0.00200953 |
| *IKBIP* | 1.098310643 | 0.00015858 | 0.00202941 |
| *MMP2* | -1.39673833 | 0.00015829 | 0.00202941 |
| *SKP2* | -3.286846763 | 0.0001586 | 0.00202941 |
| *ADAMTS2* | -2.614233305 | 0.00015919 | 0.00203225 |
| *MCM10* | -1.575468067 | 0.00015971 | 0.00203417 |
| *AIG1* | -1.817795416 | 0.00016117 | 0.00204575 |
| *ARHGAP11A* | -1.902763878 | 0.00016152 | 0.00204787 |
| *NFKBIZ* | 2.026454851 | 0.00016305 | 0.00206492 |
| *BARD1* | -3.849750029 | 0.00016487 | 0.00208566 |
| *RGS16* | 3.648870438 | 0.00016789 | 0.00211893 |
| *LINC01186* | 2.023336254 | 0.00016937 | 0.00213285 |
| *PLK3* | 2.428607911 | 0.00016987 | 0.00213656 |
| *CIP2A* | -1.358278801 | 0.00017176 | 0.00215319 |
| *ITGA1* | 2.672906678 | 0.00017757 | 0.00221903 |
| *LOC110091776* | 2.09353118 | 0.00017914 | 0.00223561 |
| *PAEP* | -1.112329718 | 0.00018302 | 0.00227392 |
| *ATP13A3* | 1.485789219 | 0.00018627 | 0.00230657 |
| *SPAG1* | -1.007665475 | 0.00018837 | 0.00232474 |
| *LOC541472* | 3.003316858 | 0.00019026 | 0.00234316 |
| *TRIB3* | 1.777906253 | 0.00019143 | 0.00235468 |
| *HLA-DMA* | -1.469788823 | 0.00019347 | 0.00237102 |
| *MX2* | -2.483049275 | 0.00019361 | 0.00237102 |
| *MYBL2* | -2.734563286 | 0.00019747 | 0.00241298 |
| *SPC24* | -2.573554443 | 0.00020083 | 0.00245138 |
| *ABAT* | 2.370125338 | 0.0002021 | 0.00246412 |
| *SASS6* | -2.002336877 | 0.00020752 | 0.00252472 |
| *ATF3* | 2.05893989 | 0.00020931 | 0.00254376 |
| *STMN3* | -1.517425384 | 0.00022812 | 0.0027543 |
| *TRIM59* | -1.988476086 | 0.00023247 | 0.00280376 |
| *CASP8AP2* | -1.058771318 | 0.00023595 | 0.00284265 |
| *OASL* | -1.846164784 | 0.00023714 | 0.00285393 |
| *INPP5D* | 1.782599474 | 0.00023907 | 0.00287395 |
| *LOC100128885* | -3.878028331 | 0.00024203 | 0.00290336 |
| *CMBL* | 2.389381935 | 0.0002426 | 0.00290699 |
| *TRIM47* | -1.659920546 | 0.00024803 | 0.00295934 |
| *LOC101929460* | -3.895831469 | 0.00025285 | 0.00300714 |
| *LYPLAL1-DT* | 3.098474759 | 0.00025676 | 0.00304718 |
| *MTHFD2L* | 2.432914899 | 0.00025738 | 0.0030513 |
| *SLC37A4* | -1.084624692 | 0.00026206 | 0.00310232 |
| *CXCL3* | 2.02224944 | 0.00026451 | 0.00312246 |
| *EDIL3* | -3.837323833 | 0.00026666 | 0.0031445 |
| *PHKB* | -1.07223999 | 0.00027633 | 0.00324479 |
| *TMPO* | -1.697094505 | 0.00027601 | 0.00324479 |
| *CCNF* | -2.394345887 | 0.00027713 | 0.00325075 |
| *CKMT2-AS1* | 1.355770967 | 0.00028626 | 0.00334379 |
| *FICD* | 1.08105458 | 0.00028797 | 0.0033602 |
| *HMGA1* | 1.135562059 | 0.00028915 | 0.00337048 |
| *LOC101929427* | -3.927148547 | 0.00029006 | 0.00337758 |
| *ARNT2* | -1.305021263 | 0.00029109 | 0.00338516 |
| *CRISPLD1* | -3.724661899 | 0.00029132 | 0.00338516 |
| *PSD3* | -1.851375541 | 0.00029419 | 0.00340793 |
| *SLC25A37* | 1.003942498 | 0.00029931 | 0.00346351 |
| *IFI44L* | -3.014323926 | 0.00030003 | 0.00346827 |
| *PID1* | 1.946414891 | 0.00030099 | 0.00347579 |
| *MARCHF3* | 1.619546344 | 0.00030864 | 0.00355304 |
| *NPTX1* | -1.857454866 | 0.00032628 | 0.00375223 |
| *LIPA* | -1.515426954 | 0.00032734 | 0.00376047 |
| *LOC101928303* | 2.621494671 | 0.00033687 | 0.00385812 |
| *SNX7* | -1.095423033 | 0.00034206 | 0.00391348 |
| *LNCOG* | 2.071899755 | 0.00034841 | 0.00396981 |
| *CENPQ* | -1.321488383 | 0.00035987 | 0.00408362 |
| *DEPP1* | -2.966017599 | 0.00036279 | 0.00411232 |
| *EME1* | -2.290609576 | 0.00036613 | 0.00414201 |
| *GEMIN2* | -1.058799376 | 0.00036734 | 0.00415149 |
| *DEPDC1* | -2.270358596 | 0.00037291 | 0.00419737 |
| *AR* | -2.005210586 | 0.00038219 | 0.00429757 |
| *C3* | 1.824244126 | 0.00039841 | 0.00446126 |
| *MCM6* | -1.672839515 | 0.00039876 | 0.00446126 |
| *AEBP1* | -3.505636097 | 0.00040119 | 0.00448401 |
| *SLCO4A1* | 2.212780735 | 0.00040361 | 0.00450646 |
| *APOC1* | -2.307574621 | 0.00040751 | 0.00454095 |
| *LINC02057* | -1.249420198 | 0.00040855 | 0.00454798 |
| *LINC-PINT* | 1.207193945 | 0.00043897 | 0.00485264 |
| *EPSTI1* | -3.0566381 | 0.00045085 | 0.00497408 |
| *G0S2* | 1.824765646 | 0.00045146 | 0.00497585 |
| *CLEC2B* | -1.350896397 | 0.0004626 | 0.00508857 |
| *DLG3* | -1.509237447 | 0.00046495 | 0.00510767 |
| *CCBE1* | -1.736512478 | 0.00046944 | 0.00514858 |
| *AKR1C2* | -2.668927559 | 0.00047052 | 0.00515539 |
| *IFT20* | 1.04333474 | 0.00047697 | 0.00520472 |
| *RNASE1* | -2.321183007 | 0.00047736 | 0.00520472 |
| *SLC7A4* | -2.110200614 | 0.00048295 | 0.00526052 |
| *MNDA* | -2.970776154 | 0.00048551 | 0.00528313 |
| *GALNT7* | -1.437689631 | 0.00048652 | 0.00528904 |
| *GBP4* | -2.037010668 | 0.00048704 | 0.00528947 |
| *NEK3* | -2.246063079 | 0.00049155 | 0.00533327 |
| *CDC42EP2* | 1.363894724 | 0.00049695 | 0.00537612 |
| *EHMT1* | -1.23683338 | 0.00050262 | 0.00543224 |
| *GNA15* | 2.977842285 | 0.00050331 | 0.00543443 |
| *DDO* | -3.022901252 | 0.00053747 | 0.00572003 |
| *PSTPIP2* | 1.031929477 | 0.00054039 | 0.00574559 |
| *ADAM12* | -2.217799373 | 0.00054189 | 0.00575604 |
| *OXCT1* | -1.287087302 | 0.00055857 | 0.00590502 |
| *MAPRE2* | -1.288401237 | 0.0005673 | 0.00597459 |
| *MX1* | -1.856960819 | 0.0005717 | 0.00600395 |
| *LOC652276* | 2.09448462 | 0.00057664 | 0.00605009 |
| *TCN2* | -1.464289535 | 0.00058953 | 0.00617948 |
| *MRAS* | 2.538864392 | 0.00059384 | 0.00621879 |
| *RALY-AS1* | 1.100982601 | 0.00059801 | 0.00625079 |
| *TNNC1* | -2.279436699 | 0.00060025 | 0.00626824 |
| *PTPN6* | -2.555277092 | 0.00060296 | 0.00628566 |
| *OAS3* | -1.780364868 | 0.00060674 | 0.00631826 |
| *LINC00592* | -3.601238696 | 0.00061468 | 0.00637117 |
| *TYROBP* | -2.405352597 | 0.00062705 | 0.00646167 |
| *CEMIP* | -2.312474081 | 0.00062882 | 0.00646953 |
| *TMEM30A* | 1.023773227 | 0.00063477 | 0.00652469 |
| *CRABP2* | -3.12365054 | 0.00063591 | 0.00653037 |
| *RAD54B* | -2.221698222 | 0.00064155 | 0.00658229 |
| *FGF2* | 1.325672715 | 0.00064527 | 0.00661432 |
| *RTTN* | 2.257819694 | 0.00064643 | 0.00662006 |
| *TNFRSF11B* | 1.26221353 | 0.00064891 | 0.00663938 |
| *MTFR2* | -1.316026112 | 0.00065009 | 0.00664532 |
| *DEPDC7* | 1.725905036 | 0.00065135 | 0.00665209 |
| *PHC2* | 1.221574401 | 0.00065681 | 0.00670171 |
| *NRXN3* | -1.304484982 | 0.00066454 | 0.00676201 |
| *TCF19* | -2.322704476 | 0.00067451 | 0.006851 |
| *DOP1B* | -1.921506548 | 0.00068518 | 0.0069277 |
| *UHRF1* | -2.126747047 | 0.00068507 | 0.0069277 |
| *CD52* | -1.834157357 | 0.00070758 | 0.00712781 |
| *XRCC2* | -1.65963133 | 0.00070817 | 0.00712781 |
| *ADIRF* | -1.168527067 | 0.00071043 | 0.00713182 |
| *PARP1* | -1.256816535 | 0.00070974 | 0.00713182 |
| *TTC28-AS1* | -1.352193393 | 0.00072419 | 0.00723019 |
| *SBSN* | -1.656974981 | 0.00073924 | 0.00736063 |
| *HRAT17* | 1.851528716 | 0.00074375 | 0.00739899 |
| *FGGY* | -1.788304695 | 0.00075295 | 0.00747709 |
| *SCG2* | -4.424580299 | 0.00076137 | 0.00753779 |
| *IL18BP* | -2.045457173 | 0.00076552 | 0.0075615 |
| *LINC01537* | 2.066574316 | 0.00077357 | 0.00762065 |
| *UBAC2-AS1* | 1.007503317 | 0.00079156 | 0.00775675 |
| *ODAPH* | 3.335598293 | 0.00082943 | 0.0080574 |
| *OAS1* | -1.889728623 | 0.00083024 | 0.00805772 |
| *NDRG2* | -3.129727432 | 0.00083401 | 0.00808725 |
| *ITGB4* | -3.524982233 | 0.00084389 | 0.00817593 |
| *IRAK2* | 1.710796219 | 0.00084616 | 0.00819082 |
| *GPR87* | 3.566558939 | 0.00085947 | 0.00830525 |
| *BTN3A1* | -1.232710049 | 0.00086141 | 0.00830952 |
| *ULBP2* | 2.151640964 | 0.00086992 | 0.00838435 |
| *DEPDC1B* | -3.338548897 | 0.00088054 | 0.00847205 |
| *KIF15* | -2.179005799 | 0.00089837 | 0.00863237 |
| *SLC16A6* | 2.63341554 | 0.00089875 | 0.00863237 |
| *RDH10* | 1.261471852 | 0.00091808 | 0.00880283 |
| *LOC730101* | -2.568716591 | 0.00092277 | 0.00884018 |
| *VGLL3* | -1.8614258 | 0.00092952 | 0.00889176 |
| *MYO5B* | -1.947063929 | 0.00093458 | 0.00892263 |
| *MPHOSPH9* | -1.296658278 | 0.00094703 | 0.00903375 |
| *AP1S3* | 2.310127414 | 0.00096959 | 0.00923304 |
| *DESI1* | 1.18126985 | 0.00099839 | 0.00946685 |
| *LOC105370203* | 2.860421768 | 0.00101178 | 0.00956123 |
| *MKS1* | -1.317387332 | 0.0010136 | 0.00957036 |
| *LAMA5* | -3.553925682 | 0.00103055 | 0.00971392 |
| *MAP4K3-DT* | 1.53572982 | 0.00102982 | 0.00971392 |
| *SLF1* | -1.29142561 | 0.00103378 | 0.00973611 |
| *RDM1* | -2.347175721 | 0.00105381 | 0.00990716 |
