# Supplementary material for: Use of non-small cell lung cancer multicellular tumor spheroids to study the impact of chemotherapy
Source: Respir Res. 2024 Apr 5;25:156. doi: 10.1186/s12931-024-02791-5 (PMC10998296; doi:10.1186/s12931-024-02791-5)
Supplement: Supplementary file 10 — Supplementary Material 10 [file 12931_2024_2791_MOESM10_ESM.docx]

Table S2: List of the genes regulated following CaPa treatment on ADCA117 MCTS. -1 < log2FC > 1, padj < 0.01

| **Genes** | **log2FoldChange** | **pvalue** | **padj** |
| --- | --- | --- | --- |
| *TIMP1* | 2.43706704 | 6.04E-84 | 6.43E-80 |
| *SUGCT* | 4.02673048 | 6.52E-47 | 3.48E-43 |
| *GDF15* | 3.77363961 | 1.05E-45 | 3.75E-42 |
| *CDKN1A* | 2.48922113 | 3.20E-44 | 8.51E-41 |
| *TUBA4A* | 1.90563231 | 1.52E-41 | 3.24E-38 |
| *C1QTNF1-AS1* | 3.40084269 | 3.52E-41 | 6.26E-38 |
| *CENPF* | -2.81444608 | 1.08E-35 | 1.65E-32 |
| *MMP3* | 6.63884971 | 2.77E-33 | 3.68E-30 |
| *LINC01588* | 4.2295899 | 3.45E-33 | 4.08E-30 |
| *NEK6* | -1.58930648 | 1.71E-32 | 1.82E-29 |
| *SP140* | 2.60644887 | 3.62E-30 | 3.50E-27 |
| *DDIT3* | 1.95285648 | 1.96E-29 | 1.74E-26 |
| *BIRC5* | -3.36251781 | 2.80E-29 | 2.30E-26 |
| *STC1* | 2.66662497 | 5.47E-29 | 4.16E-26 |
| *MDM2* | 2.18851377 | 8.56E-28 | 6.08E-25 |
| *TM4SF1* | 1.37999208 | 1.09E-26 | 7.26E-24 |
| *CCNB1* | -2.39428483 | 1.55E-26 | 9.69E-24 |
| *PTGES* | 4.23773113 | 4.42E-26 | 2.48E-23 |
| *CREM* | 2.1481686 | 9.36E-26 | 4.99E-23 |
| *IL24* | 5.6253699 | 3.23E-25 | 1.64E-22 |
| *PLAC8* | -2.09313768 | 3.75E-25 | 1.82E-22 |
| *CCNB2* | -2.75671419 | 3.14E-24 | 1.45E-21 |
| *TIGAR* | 1.68227061 | 7.53E-24 | 3.34E-21 |
| *RPL22L1* | -1.80724043 | 9.69E-24 | 4.13E-21 |
| *PTPRR* | 1.64893638 | 2.30E-23 | 9.41E-21 |
| *NAMPT* | 2.09830746 | 3.05E-23 | 1.20E-20 |
| *AURKA* | -2.09214753 | 3.52E-23 | 1.34E-20 |
| *TMEM158* | 2.58850432 | 1.76E-22 | 6.46E-20 |
| *SH2D4A* | -1.83736944 | 2.00E-22 | 7.10E-20 |
| *CD274* | 2.7569325 | 7.21E-22 | 2.40E-19 |
| *CDCP1* | 2.91761915 | 1.03E-21 | 3.34E-19 |
| *TOP2A* | -3.28024328 | 2.34E-21 | 7.34E-19 |
| *ABHD2* | 1.77858957 | 8.06E-21 | 2.39E-18 |
| *TFPI2* | 3.78649197 | 2.56E-20 | 7.37E-18 |
| *PRSS23* | -2.44692698 | 2.76E-20 | 7.74E-18 |
| *MAD2L1* | -2.18067587 | 3.57E-20 | 9.76E-18 |
| *CDCA3* | -2.73128251 | 6.31E-20 | 1.68E-17 |
| *HNRNPA1* | -1.15054027 | 9.69E-20 | 2.52E-17 |
| *PTMA* | -1.16686245 | 3.91E-19 | 9.91E-17 |
| *NUSAP1* | -2.60405766 | 4.51E-19 | 1.12E-16 |
| *CDKN3* | -2.60961799 | 5.24E-19 | 1.27E-16 |
| *ANKRD20A12P* | 1.65917148 | 5.55E-19 | 1.31E-16 |
| *KPNA2* | -1.04235267 | 1.27E-18 | 2.95E-16 |
| *AEN* | 1.3503635 | 1.86E-18 | 4.17E-16 |
| *EVI2A* | -1.81487427 | 1.88E-18 | 4.17E-16 |
| *AURKB* | -2.4480585 | 2.27E-18 | 4.93E-16 |
| *PBK* | -2.94272108 | 2.31E-18 | 4.93E-16 |
| *NECTIN4* | 4.35842577 | 2.38E-18 | 4.98E-16 |
| *HMMR* | -2.71016605 | 8.20E-18 | 1.68E-15 |
| *CDCA8* | -3.02302076 | 9.79E-18 | 1.97E-15 |
| *PINCR* | 3.63548307 | 1.19E-17 | 2.34E-15 |
| *KRT8* | -1.48702084 | 1.48E-17 | 2.86E-15 |
| *HCFC1R1* | -1.21180913 | 3.76E-17 | 7.16E-15 |
| *SLC12A8* | -1.85616718 | 5.63E-17 | 1.05E-14 |
| *STMN1* | -1.63678224 | 5.71E-17 | 1.05E-14 |
| *FAS* | 2.3413017 | 6.22E-17 | 1.12E-14 |
| *HJURP* | -3.18644523 | 6.28E-17 | 1.12E-14 |
| *CDK1* | -2.51471688 | 2.09E-16 | 3.65E-14 |
| *TNIP3* | 2.58701028 | 3.32E-16 | 5.70E-14 |
| *KIF23* | -2.6655686 | 4.16E-16 | 7.03E-14 |
| *IL1A* | 3.23679044 | 6.89E-16 | 1.15E-13 |
| *EMP1* | 2.11265306 | 1.14E-15 | 1.86E-13 |
| *TNFSF15* | 4.04250246 | 1.30E-15 | 2.07E-13 |
| *MRPL15* | 1.11056155 | 2.01E-15 | 3.02E-13 |
| *NDC80* | -3.01588101 | 2.17E-15 | 3.21E-13 |
| *HMGB2* | -2.80402953 | 3.46E-15 | 5.06E-13 |
| *TROAP* | -3.64066154 | 4.87E-15 | 7.01E-13 |
| *ATP2B1* | 1.89983491 | 5.42E-15 | 7.69E-13 |
| *LOC100288175* | 2.25534134 | 7.03E-15 | 9.86E-13 |
| *IL20* | 5.11758011 | 1.41E-14 | 1.95E-12 |
| *LINC01638* | -2.72256405 | 1.50E-14 | 2.05E-12 |
| *ARL6IP1* | -1.26145054 | 1.84E-14 | 2.49E-12 |
| *LOC102723769* | 1.54094354 | 1.89E-14 | 2.51E-12 |
| *CENPN* | -1.77342606 | 2.98E-14 | 3.92E-12 |
| *TK1* | -2.07599035 | 3.77E-14 | 4.90E-12 |
| *PCLAF* | -2.15322812 | 4.60E-14 | 5.90E-12 |
| *KYNU* | 2.80998591 | 6.50E-14 | 8.24E-12 |
| *SLC22A4* | 3.03221814 | 8.76E-14 | 1.10E-11 |
| *DLGAP5* | -3.34504867 | 9.23E-14 | 1.14E-11 |
| *LGALS3* | -1.21445842 | 9.55E-14 | 1.17E-11 |
| *KIF20A* | -4.40751694 | 1.05E-13 | 1.27E-11 |
| *TMSB4X* | -1.22672025 | 1.13E-13 | 1.35E-11 |
| *ANLN* | -2.58638055 | 1.58E-13 | 1.88E-11 |
| *RACGAP1* | -2.23933474 | 2.05E-13 | 2.36E-11 |
| *UBE2C* | -2.61184265 | 2.06E-13 | 2.36E-11 |
| *COL1A1* | -2.09385856 | 2.29E-13 | 2.59E-11 |
| *NCAPH* | -3.13640064 | 2.37E-13 | 2.66E-11 |
| *GACAT2* | -2.26130308 | 2.50E-13 | 2.78E-11 |
| *CCND2* | 2.70739458 | 2.72E-13 | 2.99E-11 |
| *SMYD3* | -1.44545303 | 2.99E-13 | 3.25E-11 |
| *ACTG1* | -1.15763483 | 3.18E-13 | 3.42E-11 |
| *CUL4B* | -1.0774153 | 3.82E-13 | 4.03E-11 |
| *BTN3A2* | -1.28493461 | 4.27E-13 | 4.41E-11 |
| *GABARAPL2* | 1.04580545 | 5.13E-13 | 5.26E-11 |
| *SBF2-AS1* | -3.3708062 | 8.51E-13 | 8.63E-11 |
| *STEAP1* | 3.48297158 | 8.61E-13 | 8.65E-11 |
| *HTR2B* | -2.66292993 | 9.28E-13 | 9.24E-11 |
| *KIF4A* | -3.10003901 | 1.00E-12 | 9.87E-11 |
| *RARRES2* | -3.08213005 | 1.09E-12 | 1.07E-10 |
| *CSF3* | 3.9454394 | 1.17E-12 | 1.14E-10 |
| *SELENBP1* | -1.5215022 | 1.19E-12 | 1.14E-10 |
| *ICAM1* | 1.73321901 | 1.26E-12 | 1.17E-10 |
| *PRR11* | -1.6773424 | 1.25E-12 | 1.17E-10 |
| *SULT1A1* | -1.12316372 | 1.24E-12 | 1.17E-10 |
| *SAT1* | 1.31883513 | 1.50E-12 | 1.39E-10 |
| *DHRS7* | 1.15169349 | 1.67E-12 | 1.52E-10 |
| *KIF2C* | -3.38970499 | 1.67E-12 | 1.52E-10 |
| *PRC1* | -2.65735745 | 2.57E-12 | 2.30E-10 |
| *RAD51AP1* | -1.85959927 | 2.97E-12 | 2.62E-10 |
| *SPC25* | -2.53742746 | 2.97E-12 | 2.62E-10 |
| *AK1* | 1.09451472 | 3.03E-12 | 2.65E-10 |
| *ZWINT* | -1.58782628 | 4.46E-12 | 3.83E-10 |
| *SERPINB8* | 1.51861405 | 4.67E-12 | 3.98E-10 |
| *LAMC2* | 1.65302143 | 5.39E-12 | 4.56E-10 |
| *HBEGF* | 3.42300874 | 5.67E-12 | 4.76E-10 |
| *HPRT1* | -1.15160658 | 1.10E-11 | 8.99E-10 |
| *CENPW* | -1.52828037 | 1.16E-11 | 9.41E-10 |
| *PTTG1* | -1.48356125 | 1.26E-11 | 1.01E-09 |
| *MTHFD1* | -1.12618234 | 1.33E-11 | 1.06E-09 |
| *CKAP2* | -1.4147303 | 1.50E-11 | 1.16E-09 |
| *MKI67* | -3.76510828 | 1.49E-11 | 1.16E-09 |
| *CIDECP1* | 1.28561495 | 1.59E-11 | 1.22E-09 |
| *AMPD3* | 2.33628662 | 1.69E-11 | 1.29E-09 |
| *GADD45A* | 1.74639311 | 1.83E-11 | 1.37E-09 |
| *MATN2* | -1.84094736 | 1.99E-11 | 1.47E-09 |
| *SMC2* | 1.27583137 | 1.99E-11 | 1.47E-09 |
| *CEP250* | -1.33250502 | 2.48E-11 | 1.81E-09 |
| *CKAP2L* | -1.98503658 | 3.37E-11 | 2.38E-09 |
| *VEPH1* | 1.47276218 | 3.85E-11 | 2.70E-09 |
| *IER3* | 2.3689586 | 5.15E-11 | 3.56E-09 |
| *DUSP6* | 2.36837339 | 5.68E-11 | 3.88E-09 |
| *YBX3* | 1.28161191 | 5.67E-11 | 3.88E-09 |
| *CYB5A* | -1.38003201 | 6.42E-11 | 4.30E-09 |
| *PSG2* | 1.50718243 | 6.54E-11 | 4.33E-09 |
| *BUB1* | -2.75922781 | 8.02E-11 | 5.18E-09 |
| *TM4SF19* | 1.5596914 | 8.36E-11 | 5.37E-09 |
| *PRIM1* | -2.29639407 | 9.03E-11 | 5.73E-09 |
| *IL1B* | 3.04589787 | 9.88E-11 | 6.23E-09 |
| *TMEM233* | 1.6036334 | 1.06E-10 | 6.61E-09 |
| *EP400P1* | -1.92360501 | 1.15E-10 | 7.05E-09 |
| *EHF* | 4.55696486 | 1.36E-10 | 8.26E-09 |
| *HMGN2* | -1.06048404 | 1.56E-10 | 9.42E-09 |
| *NABP1* | 1.14132357 | 1.56E-10 | 9.42E-09 |
| *LINC01704* | 5.13005309 | 1.90E-10 | 1.13E-08 |
| *PHYHD1* | -1.13091598 | 1.98E-10 | 1.17E-08 |
| *H2AFZ* | -1.08520441 | 2.03E-10 | 1.19E-08 |
| *AXL* | -1.20849059 | 2.43E-10 | 1.41E-08 |
| *CFDP1* | -1.14048326 | 2.52E-10 | 1.45E-08 |
| *TPX2* | -2.53171769 | 2.53E-10 | 1.45E-08 |
| *ASB1* | 1.9101945 | 3.45E-10 | 1.96E-08 |
| *NINJ1* | 1.40005132 | 3.91E-10 | 2.22E-08 |
| *PI3* | 3.75364853 | 3.95E-10 | 2.23E-08 |
| *THY1* | -1.03039018 | 4.03E-10 | 2.26E-08 |
| *RHNO1* | -1.14763376 | 4.10E-10 | 2.28E-08 |
| *CLSPN* | -1.84186631 | 4.76E-10 | 2.61E-08 |
| *TSC22D1* | 1.5419517 | 4.82E-10 | 2.62E-08 |
| *PPIF* | 1.41370263 | 4.94E-10 | 2.67E-08 |
| *LOC107985911* | -1.35861751 | 5.11E-10 | 2.75E-08 |
| *SGO2* | -2.25166477 | 5.23E-10 | 2.80E-08 |
| *SPDL1* | -1.56596683 | 5.53E-10 | 2.94E-08 |
| *MAP3K7CL* | 1.60048001 | 6.18E-10 | 3.28E-08 |
| *HMGB3* | -1.06899559 | 7.36E-10 | 3.86E-08 |
| *ZNF382* | 1.14640834 | 7.52E-10 | 3.93E-08 |
| *RGS4* | -1.92820483 | 8.42E-10 | 4.38E-08 |
| *ANGPTL4* | 4.76684816 | 8.58E-10 | 4.44E-08 |
| *H2AFV* | -1.08206244 | 9.19E-10 | 4.73E-08 |
| *TNIP1* | 1.36938834 | 9.51E-10 | 4.85E-08 |
| *S100A10* | -1.22033243 | 1.37E-09 | 6.90E-08 |
| *POU2F2* | 1.61990626 | 1.66E-09 | 8.31E-08 |
| *SLC39A10* | -1.38131504 | 1.69E-09 | 8.36E-08 |
| *STOM* | 1.69901587 | 1.75E-09 | 8.56E-08 |
| *CDC20* | -2.64539533 | 1.77E-09 | 8.63E-08 |
| *DPF3* | 1.41080976 | 1.85E-09 | 8.96E-08 |
| *CAVIN2* | -1.79062124 | 2.11E-09 | 1.01E-07 |
| *C7orf69* | -2.7019361 | 2.30E-09 | 1.09E-07 |
| *LINC01583* | -2.1551208 | 2.32E-09 | 1.10E-07 |
| *SERPIND1* | 3.2991231 | 2.61E-09 | 1.23E-07 |
| *RPLP0P2* | 1.99712576 | 2.67E-09 | 1.25E-07 |
| *PMP22* | -1.33151242 | 3.24E-09 | 1.50E-07 |
| *PNP* | 1.05607054 | 3.63E-09 | 1.67E-07 |
| *HIST1H2AH* | -2.28800055 | 3.68E-09 | 1.68E-07 |
| *MYH10* | -2.1896522 | 4.02E-09 | 1.83E-07 |
| *PDE1C* | -2.30108036 | 4.08E-09 | 1.85E-07 |
| *AOX1* | 2.65560672 | 5.14E-09 | 2.29E-07 |
| *ALDH7A1* | -1.45266492 | 5.44E-09 | 2.41E-07 |
| *ODC1* | 1.54276884 | 5.91E-09 | 2.58E-07 |
| *PIEZO2* | -2.98580303 | 6.21E-09 | 2.70E-07 |
| *LOC100505622* | 1.59741787 | 7.19E-09 | 3.11E-07 |
| *PARPBP* | -1.95963932 | 7.70E-09 | 3.31E-07 |
| *COL1A2* | -1.44231626 | 9.40E-09 | 4.00E-07 |
| *SEPTIN6* | -1.25602054 | 9.43E-09 | 4.00E-07 |
| *SLFN11* | -1.37567201 | 9.47E-09 | 4.01E-07 |
| *BUB1B* | -2.7444807 | 1.09E-08 | 4.57E-07 |
| *BEX2* | 1.74377766 | 1.11E-08 | 4.63E-07 |
| *SPAG5* | -2.89504923 | 1.13E-08 | 4.70E-07 |
| *RRM2B* | 1.30577952 | 1.15E-08 | 4.73E-07 |
| *FOXM1* | -3.08025538 | 1.18E-08 | 4.81E-07 |
| *PPP1R14B-AS1* | 1.16760626 | 1.19E-08 | 4.84E-07 |
| *PLAUR* | 1.23449711 | 1.21E-08 | 4.89E-07 |
| *ESCO2* | -2.33155574 | 1.23E-08 | 4.96E-07 |
| *NRM* | -1.44851595 | 1.33E-08 | 5.36E-07 |
| *GPR87* | 4.59767423 | 1.41E-08 | 5.65E-07 |
| *NBEAP1* | 4.67948412 | 1.62E-08 | 6.44E-07 |
| *RRM1* | -1.0260976 | 1.79E-08 | 7.08E-07 |
| *TNS3* | -1.27777191 | 2.04E-08 | 8.04E-07 |
| *LINC01426* | 1.44898358 | 2.20E-08 | 8.55E-07 |
| *NREP* | -3.05588608 | 2.32E-08 | 8.97E-07 |
| *MCM3* | -1.30473529 | 2.35E-08 | 9.06E-07 |
| *NCAPD2* | -2.14719053 | 2.64E-08 | 1.00E-06 |
| *S100A3* | -1.56091388 | 2.94E-08 | 1.11E-06 |
| *PPP1R15A* | 1.2559011 | 3.05E-08 | 1.15E-06 |
| *LETM2* | 1.08459655 | 3.29E-08 | 1.22E-06 |
| *FMNL2* | 1.94849618 | 3.48E-08 | 1.29E-06 |
| *FHOD1* | -1.16037317 | 3.88E-08 | 1.42E-06 |
| *LOC100506178* | 3.86620344 | 3.87E-08 | 1.42E-06 |
| *CEP70* | -1.66712592 | 4.04E-08 | 1.48E-06 |
| *LINC02015* | 2.61762823 | 4.28E-08 | 1.56E-06 |
| *SEC11C* | 1.27478267 | 5.21E-08 | 1.88E-06 |
| *LINC01224* | -3.11780895 | 5.27E-08 | 1.89E-06 |
| *IL6* | 3.31927145 | 5.56E-08 | 1.98E-06 |
| *CKS1B* | -1.26238361 | 6.12E-08 | 2.17E-06 |
| *GTSE1* | -2.74123846 | 6.18E-08 | 2.19E-06 |
| *UBA7* | -1.8003549 | 6.79E-08 | 2.36E-06 |
| *EIF5A2* | 1.22170319 | 6.96E-08 | 2.41E-06 |
| *NRG1* | 1.83063943 | 7.29E-08 | 2.51E-06 |
| *HIST1H4C* | -1.7129779 | 7.54E-08 | 2.59E-06 |
| *MPC2* | 1.03478733 | 7.57E-08 | 2.59E-06 |
| *PLEKHA4* | -1.88753421 | 7.71E-08 | 2.63E-06 |
| *ARID5B* | 1.16049799 | 7.86E-08 | 2.67E-06 |
| *ENO3* | -1.36181343 | 8.54E-08 | 2.88E-06 |
| *WFDC21P* | 1.9604139 | 8.71E-08 | 2.92E-06 |
| *PHLDA1* | 1.34459053 | 9.40E-08 | 3.12E-06 |
| *C1R* | -1.54296174 | 9.82E-08 | 3.24E-06 |
| *TSPAN14* | 1.69768159 | 9.95E-08 | 3.27E-06 |
| *SMC4* | -1.56416005 | 1.15E-07 | 3.76E-06 |
| *CHRDL1* | -1.51538166 | 1.16E-07 | 3.78E-06 |
| *MT2A* | 1.00949446 | 1.19E-07 | 3.85E-06 |
| *SPAG1* | -1.64936765 | 1.20E-07 | 3.87E-06 |
| *CDCA7L* | -1.45407968 | 1.25E-07 | 4.02E-06 |
| *KIF20B* | -1.78286861 | 1.32E-07 | 4.25E-06 |
| *NUF2* | -3.14570595 | 1.36E-07 | 4.33E-06 |
| *NDUFAF8* | 1.09937747 | 1.41E-07 | 4.46E-06 |
| *RSPO3* | 3.88312387 | 1.51E-07 | 4.75E-06 |
| *FEN1* | -1.21637225 | 1.54E-07 | 4.82E-06 |
| *ODAPH* | 4.18683679 | 1.55E-07 | 4.85E-06 |
| *FANCD2* | -2.57925659 | 1.57E-07 | 4.88E-06 |
| *OIP5-AS1* | -1.1243876 | 1.57E-07 | 4.89E-06 |
| *TNFRSF10B* | 1.59997138 | 1.71E-07 | 5.22E-06 |
| *UBE2T* | -1.42756611 | 1.86E-07 | 5.65E-06 |
| *HLTF* | -1.43457374 | 1.87E-07 | 5.66E-06 |
| *RFC5* | -1.24052145 | 2.05E-07 | 6.16E-06 |
| *PIR* | -1.13819088 | 2.14E-07 | 6.41E-06 |
| *DYRK3* | 2.26829362 | 2.16E-07 | 6.44E-06 |
| *RFC4* | -1.28821646 | 2.18E-07 | 6.48E-06 |
| *SMC1A* | -1.00355734 | 2.24E-07 | 6.66E-06 |
| *WDR76* | -2.63771239 | 2.61E-07 | 7.67E-06 |
| *IL13RA2* | 2.99111027 | 2.75E-07 | 8.03E-06 |
| *PLK1* | -1.39351637 | 2.94E-07 | 8.52E-06 |
| *SFTA1P* | 1.46569107 | 3.01E-07 | 8.68E-06 |
| *CENPE* | -2.15217101 | 3.24E-07 | 9.29E-06 |
| *TP53I3* | 1.15060731 | 3.32E-07 | 9.48E-06 |
| *PPP4R2* | 1.25000209 | 3.81E-07 | 1.08E-05 |
| *RBKS* | 1.99799171 | 3.90E-07 | 1.10E-05 |
| *TTK* | -3.18939967 | 4.08E-07 | 1.15E-05 |
| *THBS1* | -1.51031821 | 4.77E-07 | 1.34E-05 |
| *BDKRB2* | 2.81383131 | 5.37E-07 | 1.49E-05 |
| *HERC4* | 1.18578452 | 5.51E-07 | 1.53E-05 |
| *C15orf48* | 1.89746683 | 5.79E-07 | 1.59E-05 |
| *POPDC2* | 3.56596519 | 5.80E-07 | 1.59E-05 |
| *HMGN3* | -1.17386349 | 6.05E-07 | 1.65E-05 |
| *ZP3* | 2.25062323 | 6.05E-07 | 1.65E-05 |
| *HIST1H3B* | -3.26122813 | 6.12E-07 | 1.66E-05 |
| *SPHK1* | 1.05968575 | 6.21E-07 | 1.68E-05 |
| *TCERG1L-AS1* | 2.51646693 | 6.22E-07 | 1.68E-05 |
| *EMP2* | -2.30201999 | 6.55E-07 | 1.76E-05 |
| *GABARAPL1* | 1.61489128 | 6.59E-07 | 1.76E-05 |
| *PPP2R2B* | -3.5029246 | 6.58E-07 | 1.76E-05 |
| *HIST1H3C* | -4.96120116 | 6.74E-07 | 1.80E-05 |
| *SKA3* | -2.10975257 | 7.34E-07 | 1.95E-05 |
| *TNFRSF12A* | 1.27254828 | 7.35E-07 | 1.95E-05 |
| *LOC101927822* | 3.75314977 | 8.06E-07 | 2.12E-05 |
| *SLC11A2* | 1.13817065 | 8.26E-07 | 2.16E-05 |
| *MMP2* | -2.0562456 | 8.78E-07 | 2.29E-05 |
| *EXOC4* | -1.34087256 | 8.83E-07 | 2.30E-05 |
| *AKR1B1* | 1.22646253 | 1.08E-06 | 2.77E-05 |
| *G0S2* | 2.55496066 | 1.10E-06 | 2.81E-05 |
| *BCHE* | -2.32793638 | 1.11E-06 | 2.84E-05 |
| *SLC43A3* | 1.14085291 | 1.18E-06 | 2.98E-05 |
| *IAH1* | -1.0622439 | 1.19E-06 | 3.00E-05 |
| *SERPINF1* | -1.38224846 | 1.21E-06 | 3.05E-05 |
| *CENPM* | -1.82711319 | 1.30E-06 | 3.26E-05 |
| *GFRA1* | -2.52110768 | 1.36E-06 | 3.38E-05 |
| *KPNA1* | 1.12233191 | 1.39E-06 | 3.46E-05 |
| *MND1* | -2.03223388 | 1.41E-06 | 3.50E-05 |
| *DLEU2* | -2.81505713 | 1.42E-06 | 3.50E-05 |
| *AOPEP* | -1.14400311 | 1.44E-06 | 3.54E-05 |
| *PAPPA* | 2.69260464 | 1.44E-06 | 3.56E-05 |
| *ASPM* | -3.1967763 | 1.49E-06 | 3.67E-05 |
| *GMNN* | -1.18409312 | 1.50E-06 | 3.67E-05 |
| *HIRIP3* | -1.51864889 | 1.62E-06 | 3.91E-05 |
| *CCDC80* | -1.30862706 | 1.68E-06 | 4.03E-05 |
| *KNL1* | -2.07075095 | 1.72E-06 | 4.10E-05 |
| *ST3GAL1* | 1.14931953 | 1.73E-06 | 4.11E-05 |
| *GINS2* | -1.95225865 | 1.82E-06 | 4.31E-05 |
| *CXCL8* | 2.83755745 | 1.93E-06 | 4.56E-05 |
| *TMEM130* | -3.7129658 | 2.12E-06 | 4.98E-05 |
| *ALDH1A3* | 2.5702581 | 2.13E-06 | 5.01E-05 |
| *SGO1* | -2.20725491 | 2.25E-06 | 5.25E-05 |
| *FANCI* | -1.83584742 | 2.30E-06 | 5.37E-05 |
| *PRNP* | 1.22714388 | 2.31E-06 | 5.38E-05 |
| *CLSTN2* | -3.39694763 | 2.35E-06 | 5.45E-05 |
| *ANP32E* | -1.11337536 | 2.36E-06 | 5.46E-05 |
| *HIST1H2BG* | 2.08404761 | 2.42E-06 | 5.57E-05 |
| *COL5A2* | -2.21363083 | 2.43E-06 | 5.60E-05 |
| *NUPR1* | -1.38362773 | 2.45E-06 | 5.62E-05 |
| *FCMR* | 2.88246871 | 2.53E-06 | 5.80E-05 |
| *TPBG* | 1.71389081 | 2.58E-06 | 5.89E-05 |
| *CCNA2* | -4.08596749 | 2.65E-06 | 6.03E-05 |
| *UTRN* | -1.47884388 | 2.69E-06 | 6.11E-05 |
| *NTRK3* | -1.86545974 | 2.76E-06 | 6.23E-05 |
| *SERPINB2* | 1.78969164 | 2.81E-06 | 6.34E-05 |
| *HIST1H1B* | -2.71292537 | 2.87E-06 | 6.46E-05 |
| *LRR1* | -1.0167434 | 2.97E-06 | 6.68E-05 |
| *TRAIP* | -1.67556544 | 3.08E-06 | 6.87E-05 |
| *BTN2A1* | 1.15210662 | 3.13E-06 | 6.95E-05 |
| *VRK1* | -1.04869039 | 3.15E-06 | 6.97E-05 |
| *PLPP3* | 1.82921765 | 3.20E-06 | 7.06E-05 |
| *EDA2R* | 1.32241019 | 3.22E-06 | 7.08E-05 |
| *GDPD1* | 2.16507716 | 3.54E-06 | 7.74E-05 |
| *MCM7* | -1.35676326 | 3.55E-06 | 7.75E-05 |
| *DLGAP1-AS2* | 1.60216566 | 3.71E-06 | 8.07E-05 |
| *NKX3-1* | 1.52347944 | 3.71E-06 | 8.07E-05 |
| *NEK2* | -2.74799143 | 3.73E-06 | 8.10E-05 |
| *NRXN3* | -1.86243771 | 4.12E-06 | 8.84E-05 |
| *CCDC148* | 1.87829052 | 4.22E-06 | 9.02E-05 |
| *TMCC3* | 3.91675149 | 4.69E-06 | 9.95E-05 |
| *NASP* | -1.01454688 | 4.72E-06 | 9.99E-05 |
| *SLC25A37* | 1.32121903 | 4.74E-06 | 0.00010016 |
| *SCAPER* | -1.45177501 | 4.82E-06 | 0.00010171 |
| *EEF1AKMT4* | 1.04358735 | 4.92E-06 | 0.00010348 |
| *GFPT2* | 1.72631398 | 5.21E-06 | 0.00010924 |
| *SOD2* | 1.86660353 | 5.50E-06 | 0.00011494 |
| *PLAU* | 1.37012277 | 6.09E-06 | 0.00012678 |
| *INO80C* | 1.05603692 | 6.36E-06 | 0.00013199 |
| *MBNL2* | -1.14906607 | 6.97E-06 | 0.0001439 |
| *BRCA1* | -1.91302747 | 7.25E-06 | 0.00014913 |
| *OXTR* | -2.11552078 | 7.64E-06 | 0.00015614 |
| *C11orf91* | 2.03146934 | 7.72E-06 | 0.00015749 |
| *HIST1H1D* | -2.89704639 | 8.19E-06 | 0.00016555 |
| *ZC4H2* | -1.94708995 | 8.23E-06 | 0.00016598 |
| *GNG4* | 1.74527251 | 8.35E-06 | 0.00016792 |
| *CXCL1* | 3.22042465 | 8.49E-06 | 0.0001703 |
| *SLITRK4* | 1.81025994 | 8.98E-06 | 0.0001794 |
| *ADIPOR1* | 1.04359198 | 9.00E-06 | 0.00017959 |
| *CXCL3* | 2.44043665 | 9.35E-06 | 0.00018572 |
| *RND3* | 1.00869371 | 9.94E-06 | 0.00019536 |
| *SOX4* | -1.55784178 | 1.00E-05 | 0.00019647 |
| *DYNC1H1* | 1.05526829 | 1.01E-05 | 0.00019685 |
| *TENM2* | -3.20332504 | 1.03E-05 | 0.00020084 |
| *PHGDH* | -1.15571595 | 1.03E-05 | 0.00020131 |
| *SNX8* | 1.02306256 | 1.04E-05 | 0.00020273 |
| *HSD17B11* | -1.05770365 | 1.05E-05 | 0.00020375 |
| *SSBP2* | -1.50011464 | 1.12E-05 | 0.00021624 |
| *VEGFA* | 1.33478112 | 1.13E-05 | 0.00021685 |
| *RTN1* | 4.45463675 | 1.27E-05 | 0.0002419 |
| *ANGPTL2* | -1.97504405 | 1.28E-05 | 0.00024304 |
| *DIAPH2* | -1.4251672 | 1.32E-05 | 0.00024849 |
| *FAM111B* | -1.83370309 | 1.32E-05 | 0.00024969 |
| *SNHG15* | 1.27643072 | 1.38E-05 | 0.00026019 |
| *MELK* | -2.16741747 | 1.39E-05 | 0.00026166 |
| *ERLIN1* | -1.29603165 | 1.41E-05 | 0.00026342 |
| *SNHG1* | 1.65956189 | 1.44E-05 | 0.00026668 |
| *SKA1* | -2.35400147 | 1.45E-05 | 0.00026824 |
| *TAF13* | 1.33633863 | 1.45E-05 | 0.00026824 |
| *SYT1* | -1.28915768 | 1.50E-05 | 0.00027568 |
| *ETV4* | 1.83816902 | 1.50E-05 | 0.00027687 |
| *FARS2* | -1.18602439 | 1.53E-05 | 0.00028179 |
| *TMEM237* | -1.15927915 | 1.56E-05 | 0.00028528 |
| *ISCU* | 1.3857718 | 1.58E-05 | 0.00028866 |
| *CDC25C* | -2.98665336 | 1.63E-05 | 0.00029514 |
| *TMEM106C* | -1.07858776 | 1.70E-05 | 0.00030717 |
| *HELLS* | -1.56714429 | 1.71E-05 | 0.0003081 |
| *PVT1* | 1.19586566 | 1.75E-05 | 0.00031212 |
| *TMEM14A* | -1.04944343 | 1.75E-05 | 0.00031212 |
| *DGLUCY* | -1.09912883 | 1.81E-05 | 0.00031944 |
| *STX1A* | 1.58431985 | 1.91E-05 | 0.00033531 |
| *USP53* | 1.37626291 | 1.94E-05 | 0.00033965 |
| *INPP5D* | 1.96403382 | 1.95E-05 | 0.00034173 |
| *KLHL4* | -3.57652406 | 2.19E-05 | 0.00037841 |
| *PRKCA* | -1.55710598 | 2.28E-05 | 0.00039175 |
| *ITGA2* | 1.55316892 | 2.32E-05 | 0.00039769 |
| *PMAIP1* | 1.07922841 | 2.35E-05 | 0.00040192 |
| *ASTN2* | 1.90211804 | 2.36E-05 | 0.00040391 |
| *TCAF1* | -1.29160086 | 2.46E-05 | 0.00041777 |
| *LOC100419583* | -1.15583429 | 2.48E-05 | 0.00041974 |
| *GPNMB* | -1.30030572 | 2.52E-05 | 0.00042502 |
| *LINC01356* | 2.02769404 | 2.56E-05 | 0.00043109 |
| *PLAAT4* | -1.36314387 | 2.57E-05 | 0.00043257 |
| *NFYB* | -1.15534321 | 2.69E-05 | 0.00045259 |
| *PLSCR4* | -1.33431958 | 2.76E-05 | 0.00046183 |
| *THBS3* | -1.44035014 | 2.96E-05 | 0.00049013 |
| *CCRL2* | 2.27477183 | 2.99E-05 | 0.00049534 |
| *MCM4* | -1.27237857 | 3.07E-05 | 0.00050718 |
| *RAD51B* | -3.03802487 | 3.20E-05 | 0.00052621 |
| *CXCL2* | 2.30752471 | 3.25E-05 | 0.00053246 |
| *BTG2* | 2.50358769 | 3.35E-05 | 0.00054748 |
| *CENPK* | -1.33358488 | 3.43E-05 | 0.00055776 |
| *CSF2* | 2.82906493 | 3.53E-05 | 0.00057139 |
| *SERPINB4* | 4.58509985 | 3.54E-05 | 0.00057289 |
| *BTG1* | 1.11756964 | 3.59E-05 | 0.00057732 |
| *CLU* | -1.3499437 | 3.59E-05 | 0.00057732 |
| *LINC00632* | 1.03805759 | 3.59E-05 | 0.00057732 |
| *LIF* | 2.14138758 | 3.67E-05 | 0.00058753 |
| *C1orf21* | -1.32380632 | 3.80E-05 | 0.00060442 |
| *HYLS1* | -1.0142324 | 4.07E-05 | 0.0006418 |
| *UPP1* | 1.03709566 | 4.18E-05 | 0.00065852 |
| *HIST1H1A* | -3.37329591 | 4.46E-05 | 0.00069151 |
| *ARHGAP11A* | -2.59430928 | 4.50E-05 | 0.000695 |
| *CAPSL* | -2.85741792 | 4.49E-05 | 0.000695 |
| *CIP2A* | -1.62220456 | 4.50E-05 | 0.000695 |
| *HMGN5* | -1.3666093 | 4.71E-05 | 0.00072531 |
| *FLG* | -4.38051255 | 4.95E-05 | 0.00075854 |
| *RNASEH2A* | -1.5780959 | 5.32E-05 | 0.00081374 |
| *LAMB1* | -2.02639264 | 6.04E-05 | 0.0009149 |
| *SESN2* | 2.04790965 | 6.49E-05 | 0.00097936 |
| *OR8G2P* | 2.88923185 | 6.68E-05 | 0.00100326 |
| *PSAT1* | -1.01750192 | 6.67E-05 | 0.00100326 |
| *INHBA* | 1.68286527 | 6.76E-05 | 0.00101364 |
| *PLXDC2* | -3.97095053 | 6.76E-05 | 0.00101364 |
| *LAMB3* | 1.68260732 | 6.79E-05 | 0.00101581 |
| *MCM10* | -1.79675905 | 6.92E-05 | 0.0010311 |
| *IFITM1* | -1.9116526 | 7.02E-05 | 0.00104394 |
| *ATP1B1* | 1.03003751 | 7.20E-05 | 0.00106644 |
| *CIDEC* | 1.51460412 | 7.24E-05 | 0.00107043 |
| *CDC45* | -1.41323973 | 7.47E-05 | 0.00109879 |
| *TLN2* | -1.21026665 | 7.57E-05 | 0.00110874 |
| *CDKAL1* | -1.62716327 | 7.59E-05 | 0.00110897 |
| *MTHFD2L* | 2.60362187 | 7.58E-05 | 0.00110897 |
| *SHCBP1* | -2.81495329 | 7.71E-05 | 0.001125 |
| *GPX1* | 1.04211567 | 7.79E-05 | 0.00113209 |
| *TLCD5* | 1.27169884 | 8.17E-05 | 0.00117771 |
| *HHIPL2* | -4.07693653 | 8.21E-05 | 0.00118055 |
| *PTPRN* | 2.10014735 | 8.50E-05 | 0.00121294 |
| *POC1A* | -1.39206746 | 8.92E-05 | 0.00126211 |
| *TGM2* | 1.07143944 | 8.91E-05 | 0.00126211 |
| *TMSB15B* | -1.33974975 | 9.02E-05 | 0.00127301 |
| *UBR7* | -1.55276022 | 9.08E-05 | 0.00127943 |
| *LINC01795* | 4.21658272 | 0.00010369 | 0.00144403 |
| *GDNF* | 1.74850832 | 0.000105 | 0.00145856 |
| *MYL9* | -1.49742977 | 0.0001082 | 0.00149517 |
| *PID1* | 2.04292324 | 0.0001091 | 0.00150368 |
| *PARP9* | -1.1922663 | 0.00011407 | 0.00156209 |
| *TMEM38B* | 1.16615265 | 0.00011506 | 0.00157159 |
| *CCL20* | 1.96263376 | 0.00011785 | 0.00160153 |
| *PCNX2* | 1.69749307 | 0.00011905 | 0.00161578 |
| *PSTPIP2* | 1.14081283 | 0.00012196 | 0.00165099 |
| *ORC1* | -2.77874295 | 0.00012364 | 0.00167015 |
| *ATP2C1* | 1.20549667 | 0.00012667 | 0.00170831 |
| *LURAP1L* | 1.24284048 | 0.00012789 | 0.00171817 |
| *SLC39A11* | -1.10793312 | 0.00012911 | 0.0017303 |
| *CHEK1* | -1.27075414 | 0.00013223 | 0.00176918 |
| *NAV1* | -1.21414094 | 0.0001328 | 0.00177299 |
| *FMN1* | 2.27141213 | 0.00013536 | 0.00180492 |
| *COL4A2* | -1.38561957 | 0.0001363 | 0.00181481 |
| *IFI44* | -1.4913958 | 0.00013644 | 0.00181481 |
| *LOC110091776* | 2.06159735 | 0.00013852 | 0.00184017 |
| *CHN1* | -2.72344552 | 0.00013986 | 0.00185568 |
| *ATAD2* | -1.82648468 | 0.00014037 | 0.00186006 |
| *LMO7* | -1.34678935 | 0.00014222 | 0.00188232 |
| *IMMP2L* | -3.4023167 | 0.00014282 | 0.00188788 |
| *WDHD1* | -2.35539652 | 0.00014436 | 0.00190584 |
| *MTFR2* | -1.71592382 | 0.00014463 | 0.00190707 |
| *ADGRF4* | 3.33489855 | 0.00014528 | 0.00191089 |
| *CDC25B* | -1.67031912 | 0.00014512 | 0.00191089 |
| *DIAPH3* | -1.90843977 | 0.00015463 | 0.00201893 |
| *KIFC1* | -2.04010575 | 0.00015515 | 0.00202323 |
| *MCM5* | -1.20092215 | 0.00016083 | 0.00207949 |
| *COL12A1* | -1.93814187 | 0.00016267 | 0.00209814 |
| *SLC16A6* | 2.98822213 | 0.00016297 | 0.00209895 |
| *SNX7* | -1.28874463 | 0.0001634 | 0.00209991 |
| *LYPLAL1-DT* | 3.2648425 | 0.00017333 | 0.00222222 |
| *ATP13A3* | 1.85145332 | 0.00017536 | 0.00224252 |
| *TNNC1* | -3.68367377 | 0.00017555 | 0.00224252 |
| *HADH* | -1.17843029 | 0.00018088 | 0.00230235 |
| *ZNF766* | -1.11435803 | 0.00018482 | 0.00234408 |
| *CEBPB* | 1.31795312 | 0.00018545 | 0.00234934 |
| *FBXO32* | -1.32729385 | 0.00019522 | 0.00246156 |
| *LINC01128* | 1.00665798 | 0.00019523 | 0.00246156 |
| *CCDC14* | -1.22143879 | 0.00019846 | 0.00249877 |
| *RCAN1* | 1.01733251 | 0.00020274 | 0.00253732 |
| *GALNT14* | -1.76065627 | 0.00020324 | 0.00253848 |
| *ATF3* | 2.02404295 | 0.00020442 | 0.00255022 |
| *TMSB15A* | -2.59090879 | 0.00021317 | 0.00264701 |
| *RDM1* | -3.56467118 | 0.00021497 | 0.00266308 |
| *MPHOSPH9* | -1.662162 | 0.0002181 | 0.00269566 |
| *LINC00592* | -3.91262825 | 0.00021876 | 0.00269944 |
| *LIPC* | -3.45642646 | 0.00022099 | 0.00271872 |
| *TMEM30A* | 1.15859305 | 0.00022324 | 0.00274012 |
| *LOC105378047* | 1.40595976 | 0.00023974 | 0.00291909 |
| *KIF11* | -3.79766283 | 0.00024552 | 0.00297927 |
| *KIF18A* | -1.51936398 | 0.00024779 | 0.00299916 |
| *CDC6* | -1.17139529 | 0.00025552 | 0.00308304 |
| *ANKRD37* | -4.26590265 | 0.00025613 | 0.0030869 |
| *LINC01173* | 2.54247792 | 0.00025903 | 0.00311827 |
| *CPQ* | -2.04189754 | 0.00026964 | 0.00323505 |
| *PPL* | -1.41662417 | 0.00027078 | 0.00324512 |
| *LINC02454* | 1.56566957 | 0.00027822 | 0.00332307 |
| *CDK17* | 1.27227076 | 0.00028506 | 0.00339364 |
| *TES* | 1.48048501 | 0.00028509 | 0.00339364 |
| *LINC-PINT* | 1.22671481 | 0.00029239 | 0.00346505 |
| *LINC01537* | 2.04248032 | 0.00029375 | 0.00347434 |
| *PLK4* | -3.82925007 | 0.00029545 | 0.00348843 |
| *RPL23AP7* | 1.07577271 | 0.00029567 | 0.00348843 |
| *HAPLN1* | -4.46866436 | 0.00030081 | 0.00354124 |
| *GALNT7* | -1.55997973 | 0.00030233 | 0.0035552 |
| *CIT* | -2.37727586 | 0.00030593 | 0.00358962 |
| *JADE1* | -2.58276451 | 0.00030587 | 0.00358962 |
| *PCK2* | -1.15750525 | 0.00030873 | 0.00361844 |
| *PLK3* | 2.30175224 | 0.00030947 | 0.0036232 |
| *SLC37A4* | -1.08226368 | 0.00032153 | 0.00375611 |
| *CHAF1A* | -4.01309187 | 0.00033665 | 0.00392413 |
| *VNN1* | 1.99070491 | 0.00033947 | 0.0039527 |
| *TRIM8* | 1.22563948 | 0.00034577 | 0.0040129 |
| *ALDH3B1* | -1.05715374 | 0.0003469 | 0.00402168 |
| *CAB39* | 1.05130054 | 0.0003489 | 0.00404038 |
| *NFKBIA* | 1.51097094 | 0.0003495 | 0.00404292 |
| *CCNF* | -2.87931505 | 0.00035012 | 0.00404573 |
| *ACTR3C* | -1.24412018 | 0.00035406 | 0.00408687 |
| *DRAM1* | 1.0340295 | 0.00036468 | 0.00418623 |
| *PTX3* | 1.10178051 | 0.00036503 | 0.00418623 |
| *APOBEC3B* | -1.94548121 | 0.00037058 | 0.00424077 |
| *RGS5* | -1.84543244 | 0.00037279 | 0.00426146 |
| *TRABD2A* | -1.1318301 | 0.00037946 | 0.00431921 |
| *GINS4* | -1.2364103 | 0.0003869 | 0.00438976 |
| *DEPDC7* | 1.76651811 | 0.00038791 | 0.00439655 |
| *CNTRL* | -3.59804838 | 0.00039412 | 0.00446224 |
| *CACNA2D4* | 1.44481479 | 0.00039489 | 0.00446616 |
| *DEPDC1B* | -3.65478591 | 0.00039752 | 0.00448632 |
| *HLA-F* | -1.14624086 | 0.00039793 | 0.00448632 |
| *HMGA2* | 2.78968698 | 0.00040083 | 0.00451016 |
| *ZC3H12A* | 1.335588 | 0.00040089 | 0.00451016 |
| *MAFF* | 1.9673358 | 0.00040856 | 0.00456963 |
| *POLE2* | -3.55482426 | 0.00040875 | 0.00456963 |
| *RFC3* | -1.36379844 | 0.00041122 | 0.00459238 |
| *FOLR1* | -3.75339715 | 0.0004202 | 0.00467798 |
| *COL5A1* | -2.23813134 | 0.00043859 | 0.00483225 |
| *CLDN1* | 2.02428375 | 0.00045069 | 0.00493475 |
| *ID2* | -1.16086685 | 0.00045114 | 0.00493475 |
| *MRGPRX3* | 3.27811178 | 0.00045694 | 0.00498661 |
| *RDH10* | 1.35631179 | 0.00046025 | 0.00501378 |
| *ZNF79* | 1.92203578 | 0.00046185 | 0.00502607 |
| *SBF2* | -2.28674904 | 0.00046242 | 0.00502718 |
| *FKBP7* | -1.1790338 | 0.00048804 | 0.00525466 |
| *NCAPG2* | -1.70578845 | 0.00048727 | 0.00525466 |
| *ZNF93* | -1.31776228 | 0.00048888 | 0.00525466 |
| *KIF15* | -3.11642078 | 0.00049828 | 0.00532462 |
| *LOC102724927* | -1.11306318 | 0.00049824 | 0.00532462 |
| *SYNE2* | -3.40515032 | 0.00049951 | 0.00533249 |
| *SCLT1* | -2.06395207 | 0.00050521 | 0.0053718 |
| *MPP4* | 1.07131426 | 0.00050669 | 0.00537296 |
| *RAB27B* | 1.53514981 | 0.00051337 | 0.00543393 |
| *GINS1* | -2.20293424 | 0.00051535 | 0.0054416 |
| *LINC02535* | 1.85505226 | 0.00051964 | 0.0054814 |
| *SLC7A7* | 1.54078224 | 0.00052134 | 0.0054939 |
| *AFF1-AS1* | 1.77978352 | 0.00052293 | 0.0055052 |
| *EFHC1* | -1.01299359 | 0.00052587 | 0.00553074 |
| *ARHGEF2* | 1.32383272 | 0.00053311 | 0.00559581 |
| *UBA5* | 1.35387656 | 0.00054036 | 0.00564407 |
| *LOC101927151* | 1.40388514 | 0.00055221 | 0.00574535 |
| *GEMIN2* | -1.0443336 | 0.00056228 | 0.00583306 |
| *DCBLD2* | 1.04230018 | 0.00058412 | 0.00604111 |
| *RAD54B* | -2.62971135 | 0.00058461 | 0.00604111 |
| *TRIM23* | 1.30748949 | 0.00058371 | 0.00604111 |
| *FGF2* | 1.4165798 | 0.00058646 | 0.00605442 |
| *CEP152* | -1.35290582 | 0.00060836 | 0.00625624 |
| *COL8A1* | -1.78914952 | 0.00061656 | 0.00632836 |
| *MARCHF3* | 1.57442617 | 0.0006254 | 0.00639444 |
| *ECSCR* | -2.21674168 | 0.0006505 | 0.00662561 |
| *DCUN1D3* | 1.19187117 | 0.00065546 | 0.0066643 |
| *GDNF-AS1* | 2.06207324 | 0.00065555 | 0.0066643 |
| *DCDC2* | -3.37848844 | 0.00066014 | 0.00669216 |
| *LOC541472* | 2.86888505 | 0.00066123 | 0.00669653 |
| *BLM* | -2.87594425 | 0.00067189 | 0.00679806 |
| *KRT80* | -2.19718749 | 0.00067484 | 0.00682064 |
| *AR* | -2.16848615 | 0.00067861 | 0.00683358 |
| *RRAD* | 1.60295277 | 0.0006785 | 0.00683358 |
| *NOP14-AS1* | 1.71746543 | 0.00067925 | 0.00683359 |
| *ID1* | -3.73855453 | 0.0006807 | 0.00683522 |
| *LACC1* | 1.49841948 | 0.00069217 | 0.00691778 |
| *HIST1H4E* | -1.94217564 | 0.00070259 | 0.0069957 |
| *CCN3* | -3.50049301 | 0.00071236 | 0.00706656 |
| *LINC00342* | 2.13598673 | 0.00071177 | 0.00706656 |
| *SKP2* | -2.99030276 | 0.00071161 | 0.00706656 |
| *PHKB* | -1.03081182 | 0.00072173 | 0.00714622 |
| *TMPO* | -1.6374757 | 0.00072241 | 0.00714627 |
| *PPP1R9A* | -3.62748075 | 0.00073474 | 0.00725477 |
| *DEPDC1* | -2.44234015 | 0.00073988 | 0.00728663 |
| *MYO1B* | 1.16139583 | 0.00075468 | 0.00740368 |
| *ANXA3* | -1.11536988 | 0.00075582 | 0.00740553 |
| *EDN1* | 2.54075817 | 0.00077072 | 0.00752176 |
| *CXCL14* | -3.61785218 | 0.0007796 | 0.0075783 |
| *LOC105369201* | 2.84720118 | 0.0007794 | 0.0075783 |
| *ADIRF* | -1.18117026 | 0.00079559 | 0.00771966 |
| *PTPRM* | -1.33883425 | 0.00080466 | 0.00780059 |
| *YRDC* | 1.59382534 | 0.00080575 | 0.00780408 |
| *UHRF1* | -2.52156148 | 0.00082151 | 0.00793502 |
| *MRC2* | -1.34311572 | 0.00082569 | 0.00795746 |
| *MAPRE2* | -1.37827711 | 0.00082971 | 0.00798531 |
| *SLC27A5* | -1.12258895 | 0.00085073 | 0.00817287 |
| *C3* | 1.48254536 | 0.00085306 | 0.00818049 |
| *POLA1* | -1.3568341 | 0.0008528 | 0.00818049 |
| *AIG1* | -1.60595618 | 0.00085418 | 0.00818387 |
| *LINC01508* | -3.5054695 | 0.0008581 | 0.00821401 |
| *SLCO4A1* | 2.22703945 | 0.00086498 | 0.00826462 |
| *TNFSF4* | -1.03698796 | 0.00089078 | 0.00848112 |
| *OXCT1* | -1.33270016 | 0.0008958 | 0.0085174 |
| *USP1* | -1.70966196 | 0.00089976 | 0.0085361 |
| *INKA2* | 1.83382604 | 0.00090738 | 0.00857889 |
| *HIST1H1E* | -1.44024534 | 0.00091387 | 0.0086239 |
| *ANG* | -1.35796659 | 0.00091998 | 0.00866621 |
| *ATP6V0A1* | 1.05394465 | 0.0009241 | 0.008682 |
| *ASB9* | -1.11747001 | 0.00093529 | 0.00877161 |
| *MEST* | -2.38800482 | 0.00093734 | 0.00878312 |
| *TRO* | -1.32142382 | 0.00098046 | 0.00913896 |
| *ADGRG6* | -2.05018651 | 0.00098524 | 0.00916248 |
| *CPA4* | -2.03865542 | 0.00098556 | 0.00916248 |
| *EBI3* | 1.45021859 | 0.00098725 | 0.00917017 |
| *PHF19* | -1.25016133 | 0.00099491 | 0.00922526 |
| *LOXL1* | -1.80685916 | 0.00099937 | 0.00925847 |
| *CCDC152* | -1.81320479 | 0.00102302 | 0.0094612 |
| *CBR3* | -2.78134393 | 0.00102397 | 0.00946172 |
| *KIF14* | -1.971432 | 0.00105799 | 0.00975074 |
| *SLC16A7* | -1.96150112 | 0.00106537 | 0.00981028 |
| *ARNT2* | -1.21455022 | 0.00107292 | 0.00985421 |
